# Unexpected impairment of *I*_Na_ underpins reentrant arrhythmias in a knock-in swine model of Timothy syndrome

**DOI:** 10.1038/s44161-023-00393-w

**Published:** 2023-12-11

**Authors:** Andreu Porta-Sánchez, Andrea Mazzanti, Carmen Tarifa, Deni Kukavica, Alessandro Trancuccio, Muhammad Mohsin, Elisa Zanfrini, Andrea Perota, Roberto Duchi, Kevin Hernandez-Lopez, Miguel Eduardo Jáuregui-Abularach, Valerio Pergola, Eugenio Fernandez, Rossana Bongianino, Elisa Tavazzani, Patrick Gambelli, Mirella Memmi, Simone Scacchi, Luca F. Pavarino, Piero Colli Franzone, Giovanni Lentini, David Filgueiras-Rama, Cesare Galli, Demetrio Julián Santiago, Silvia G. Priori

**Affiliations:** 1 Novel Arrhythmogenic Mechanism Program, Centro Nacional de Investigaciones Cardiovasculares (CNIC), Madrid, Spain; 2 Molecular Cardiology, IRCCS Istituti Clinici Scientifici Maugeri, Pavia, Italy; 3 Department of Molecular Medicine, University of Pavia, Pavia, Italy; 4 AVANTEA, Cremona, Italy; 5 Department of Advanced Biomedical Sciences, University of Naples Federico II, Naples, Italy; 6 Department of Mathematics, University of Milan, Milano, Italy; 7 Department of Mathematics, University of Pavia, Pavia, Italy; 8 Department of Pharmacology, University of Bari, Bari, Italy; 9 Cardiovascular Institute, Instituto de Investigación Sanitaria del Hospital Clínico San Carlos (IdISSC), Madrid, Spain; 10 Centro de Investigación Biomédica en Red de Enfermedades Cardiovasculares, Madrid, Spain

**Keywords:** Cardiovascular genetics, Arrhythmias

## Abstract

Timothy syndrome 1 (TS1) is a multi-organ form of long QT syndrome associated with life-threatening cardiac arrhythmias, the organ-level dynamics of which remain unclear. In this study, we developed and characterized a novel porcine model of TS1 carrying the causative p.Gly406Arg mutation in *CACNA1C*, known to impair Ca_V_1.2 channel inactivation. Our model fully recapitulated the human disease with prolonged QT interval and arrhythmic mortality. Electroanatomical mapping revealed the presence of a functional substrate vulnerable to reentry, stemming from an unforeseen constitutional slowing of cardiac activation. This signature substrate of TS1 was reliably identified using the reentry vulnerability index, which, we further demonstrate, can be used as a benchmark for assessing treatment efficacy, as shown by testing of multiple clinical and preclinical anti-arrhythmic compounds. Notably, in vitro experiments showed that TS1 cardiomyocytes display Ca^2+^ overload and decreased peak I_Na_ current, providing a rationale for the arrhythmogenic slowing of impulse propagation in vivo.

## Abstract (web summary)

Porta-Sanchez et al. developed a pig model for Timothy syndrome (TS), which mirrors the severe heart condition seen in children with TS and also uncovers novel triggers of these life-threatening cardiac arrhythmias, illustrating the suitability of the new TS animal model for exploring new therapies.

## Main

Timothy syndrome (TS), also called long QT syndrome type 8, is one of the most lethal variants of long QT syndrome (LQTS). Unlike most forms of the disease, TS is a syndromic, multi-organ disease associated with an extreme QT interval prolongation and a high rate of lethal ventricular arrhythmias, often resulting in death during infancy^1^. Most affected individuals harbor an identical gain-of-function mutation (p.Gly406Arg) in the alternatively spliced exon 8A of the *CACNA1C* gene (Timothy syndrome type 1 (TS1))^1^, which encodes for the α subunit of the Ca_V_1.2 Ca^2+^ channel.

Patch-clamp studies demonstrated that the p.Gly406Arg mutation selectively impairs the voltage-dependent inactivation (VDI) of the Ca_v_1.2 α subunit, thus prolonging the ventricular action potential duration (APD)^1^. Remarkable advances have been made in deciphering the pathophysiology of TS1 at the cellular level using experimental models in heterologous expression systems^1–3^, pharmacologically treated wedge preparations^4,5^, transgenic rodents^6–10^ and human induced pluripotent stem cell (iPSC)–derived cardiomyocytes^11^^,12–14^. Despite insights deriving from simulations studies^15,16^, direct experimental investigation of arrhythmogenic mechanisms at the organ level and the understanding of their clinical relevance are currently limited by the lack of large-size animal models, which permit the in-depth study of the arrhythmogenic substrate using clinical-grade equipment.

Here we present the development and characterization of a knock-in swine model of TS1, which represents, to our knowledge, the first gene-edited large-size mammal model of LQTS. We show that electroanatomical mapping (EAM) of the heart of TS1 pigs discloses unexpected insights into the electrophysiological substrate that might be useful when applied to the clinical setting. Our hypothesis in designing this study was to use EAM searching for an electrical signature of the arrhythmogenic substrate that could become the benchmark to assess the arrhythmic risk and to test the efficacy of therapeutic molecules. More broadly, we envision that identifying arrhythmogenic mechanisms underlying the development of life-threatening arrhythmias in vivo in TS1 might improve the clinical management of the disease. If confirmed, this approach might also be applied to other genetic variants of LQTS.

## Results

### Engineering and phenotyping a knock-in TS1 swine model

**Extended Data Fig. 1:**
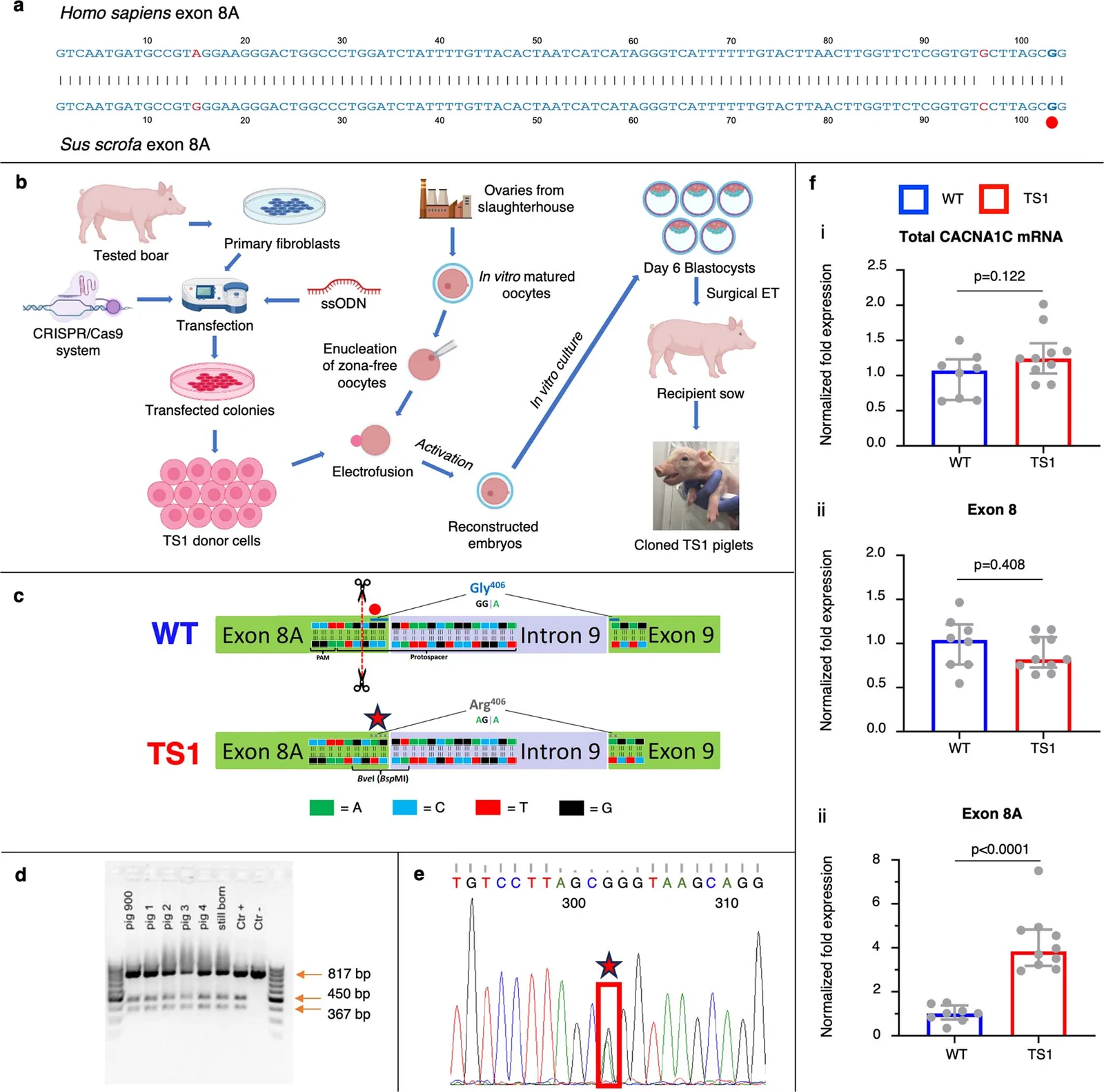
CRISPR/Cas9-mediated generation, genetic characterization, and expression profiling of TS1 pigs. **a**, Exon 8 A homology. Sequences of the exon 8 A of the *CACNA1C* in humans (above) and pig (below) shows a 98% homology (102/104 [98%] homologous bases, shown in green), with only 2 non-identical bases (shown in red). The base of interest is shown in bold. **b**, Generation of TS1 clones. A primary fibroblast cell line was established from a skin biopsy of a tested boar. Cells were co-transfected using the CRISPR/Cas9 system, specific for the *CACNA1C*-exon8, and a ssODN carrying the TS1 mutation. Colonies positive to the RFLP-PCR and Sanger sequencing analyses were used as nuclear donors to generate cloned TS1 piglets. For the SCNT experiments, oocytes were aspirated from ovaries and were matured in vitro for 42 hours. The zona pellucida was removed, matured oocytes were enucleated and electrofused to TS1 cells. The resulting constructs were activated and in vitro cultured for six days. The resulting cloned TS1 blastocysts were finally surgically transferred into a synchronized recipient sow. Farrowing was induced in sows to deliver the TS1 cloned piglets. Figure was created with Biorender.com. **c**, Genome editing design. Graphical representation of the *CACNA1C* region (Exon8A-Intron9-Exon9) selected for the genome editing procedure. On the antisense strand of the wild-type allele is identified the PAM sequence (5’-AGG-3’) following the gRNA targeting sequence (20 bp; 5’- TCGGTCCTGCTTACCCGCTA-3’). The red dotted line identifies the Cas9 cutting site, located 1 bp before the first nucleotide (red dot) of the Glycine codon (GGA), spanning the exon 8 A/exon 9 junction. In the edited allele (TS1-p.Gly406Arg) the CRISPR/Cas9-induced nucleotide substitution c. 1216 G > A (triangle) introduces a new BveI restriction site, useful for detection (RFLP-PCR) of TS1 edited colonies. The ssODN donor sequence (5’-TTTGTACTTAACTTGGTTCTCGGTGTCCTTA**GCAGgt**aagcaggaccgaggaaaaaggtcttgatttttccatttatctcatttactctttctgctcttcctggctttattcttttctga-3’) is described as follows: the uppercase characters identify the exon 8 A sequence; the lowercase characters correspond to the intron 9 sequence; the underlined character is the c.1216 G > A point mutation; the bold characters correspond to the BveI restriction site. **d**, RFLP-PCR genotyping of cloned TS1 piglets. This image presents the genotyping analysis (RFLP-PCR) performed on the N = 6 newborn (5 liveborn = Pig900, Pig1, Pig2, Pig3, Pig4; 1 stillborn) cloned TS1 piglets (N = 1 technical replicate). As expected, all the analyzed animals present an undigested WT allele (817 bp; Ctr-) and 2 additional bands (450 bp and 367 bp) resulting from the BveI digestion of the edited allele, as it happened in the nuclear donor colony (Ctr + ). Ctr- is the 8171 wild-type boar line. **e**, Sanger sequencing results of cloned TS1 piglets. This image is an example of Sanger sequencing analyses performed on TS1 clones. In this case Pig900 was PCR analyzed and the resulting amplicon was sequenced using the *CACNA1C*cr1 FW oligo. The desired monoallelic mutation is identified by a single double peak (star mark; G and A) into the Sanger chromatogram, confirming the presence of the WT and the TS1 alleles presence. **f**, Expression of mutually exclusive and constitutive exons analyzed in LV of WT and TS1 pigs. i, Total *CACNA1C* mRNA values were similar (p = 0.122) between WT (n = 8; blue) and TS1 pigs (n = 10; red) (upper panel). ii, No significant difference (p = 0.408) was found in exon 8 expression between WT and TS1 pigs (middle panel). iii, Exon 8 A expression was increased in TS1 pigs’ heart transcripts compared to WT (lower panel; p < 0.0001). Data were normalized to control gene *TBP* (TATA-box Binding Protein). Each value is represented as median and IQR. Statistical analysis was conducted using two-tailed Mann-Whitney U-test. Source data

We generated a Large White pig model of TS1 (*CACNA1C*^Gly406Arg/WT^) using CRISPR–Cas9 and somatic cell nuclear transfer (SCNT). We applied CRISPR–Cas9-induced homology-directed repair supported by single-stranded oligodeoxynucleotide in primary porcine fibroblasts to introduce the p.Gly406Arg mutation (c.1216G>A, NM_001129843) into the exon 8A of the porcine *CACNA1C* gene (Extended Data Fig. 1a and Supplementary Table 1). Viable TS1 fibroblasts were used as nuclear donors for nine SCNT experiments (Extended Data Fig. 1b) that generated 37 TS1 cloned piglets (*CACNA1C*^Gly406Arg/WT^).

The introduction of the targeted mutation (Extended Data Fig. 1c) and the absence of other variations on the coding sequence of the *CACNA1C* were confirmed at the genomic level (Extended Data Fig. 1d,e). Moreover, no unintended mutations on potential off-target sites were found. Total *CACNA1C* mRNA expression was similar between wild-type (WT) and TS1 cardiomyocytes, whereas the exon 8A expression was increased four-fold in TS1 pig heart transcripts (Extended Data Fig. 1f).

We performed electrocardiographic phenotyping using 12-lead surface electrocardiogram (ECG) and long-term ECG recordings to assess whether our animal model replicates the cardinal features observed in patients with TS1: remarkable prolongation of the Bazett’s corrected QT interval (QTc) and spontaneous occurrence of ventricular fibrillation (VF) and sudden cardiac death (SCD).

**Fig. 1:**
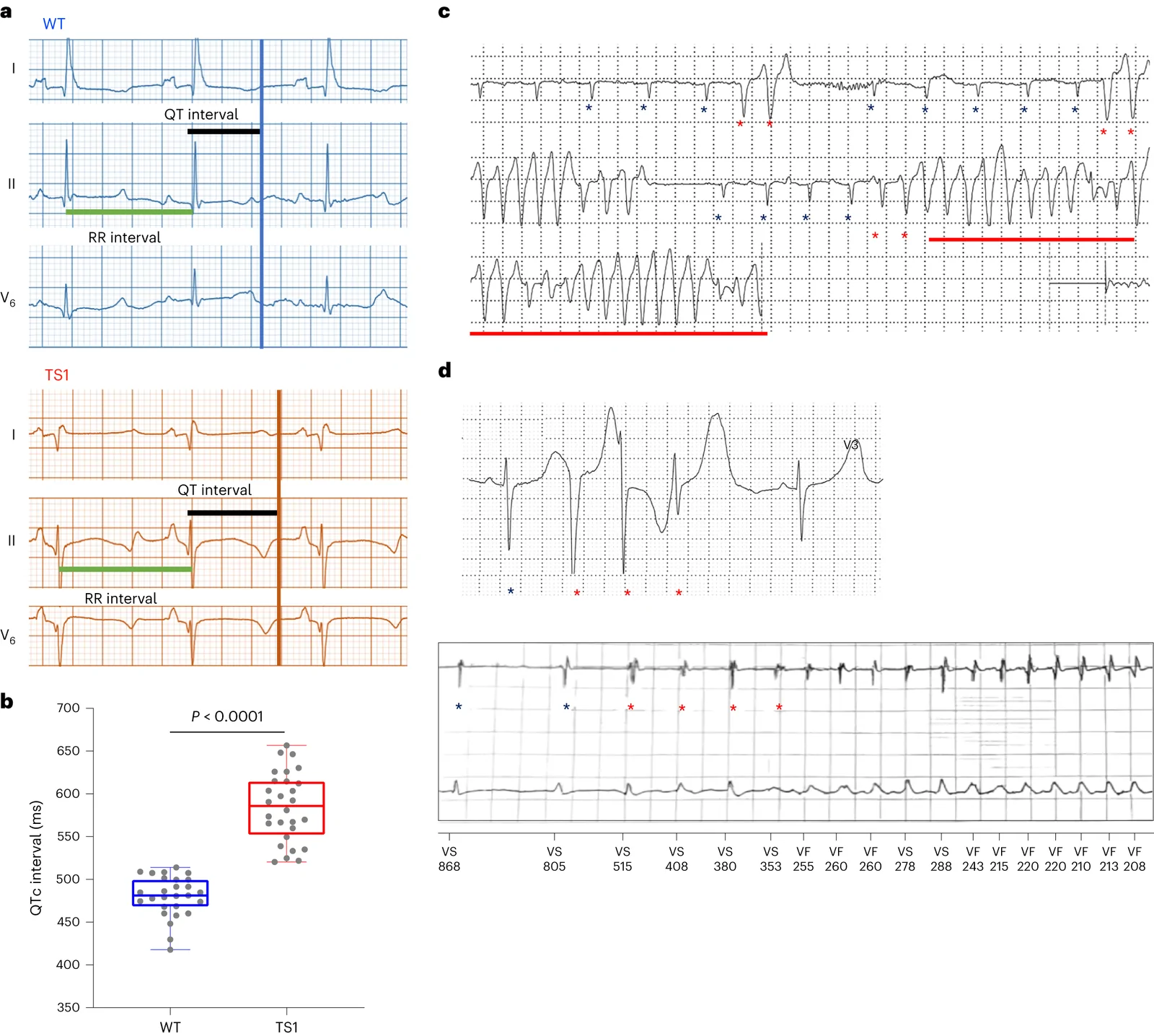
TS1 pigs recapitulate cardiac human phenotype. **a**, Representative ECG traces of WT (top, in blue) and TS1 (bottom, in red) pigs showing marked QTc interval prolongation in TS1. **b**, Box plot shows the distribution of QTc interval in *n* = 27 WT animals (minimum 419 ms, median 482 ms, IQR: 469–500 ms, maximum 514 ms) and *n* = 28 TS1 animals (minimum 521 ms, median 586 ms, IQR: 552–615 ms, maximum 657 ms). Statistical analysis was conducted using the two-tailed Mann–Whitney *U*-test (*P* < 0.0001). **c**, Example of lethal ventricular arrhythmias in TS1 pigs, triggered by PVCs (red asterisks). **d**, Documentation of a sequence of three PVCs on the 24-h Holter ECG (above) and a PVC-triggered episode of VF recorded by implantable cardioverter defibrillator (below) in a young patient with TS1. Source data

**Extended Data Fig. 2:**
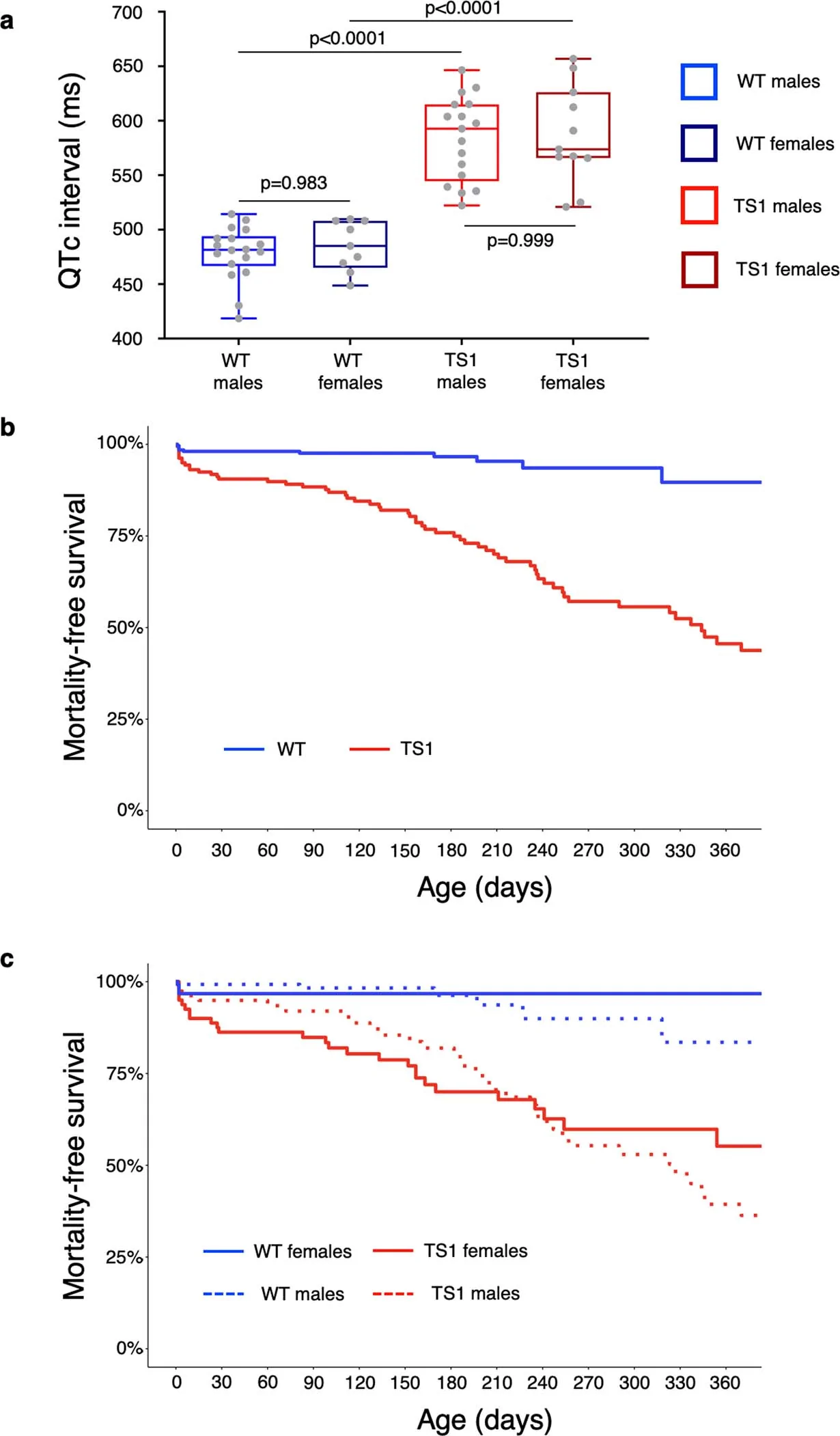
Examining gender-related variations in QTc interval and survival rates between TS1 and wild-type pigs. **a**, Gender differences in the QTc interval. No gender differences in the QTc interval were observed neither among TS1 pigs (in red tones; N = 28; p = 0.999) nor in WT pigs (in blue; N = 27; p = 0.983), but both TS1 males (red; minimum 522 ms, median 593, IQR: 545-615 ms, maximum 646 ms) and TS1 females (dark red; minimum 521 ms, median 574, IQR: 566-626 ms, maximum 657 ms) showed a significantly longer QTc interval as compared to their age- matched counterparts (in blue tones; males: minimum 419 ms, median 481, IQR: 467-494 ms, maximum 514 ms; females: minimum 449 ms, median 485, IQR: 465-508 ms, maximum 510 ms; p < 0.0001 for both). Each value is represented as median and IQR. Statistical analysis was conducted using one-way ANOVA with Šidák post-tests. **b-c**, Reduced survival in TS1 pigs. Statistical analysis was conducted using log rank test. b, Kaplan-Meier estimate of cumulative survival free from death shows reduced survival in TS1 pigs (red) as compared to WT (blue) pigs (p < 0.0001). **c**, Kaplan-Meier estimate of cumulative survival free from death stratified by gender showed no significant gender differences within genotypes (WT males [dotted blue line] vs WT females [full blue line] p = 0.610; TS1 males [dotted red line] vs. TS1 females [full red line] p = 0.270). Source data

The resting 12-lead ECG demonstrated significant QTc interval prolongation in TS1 pigs compared to age-matched and sex-matched WT controls (Fig. 1a,b, Supplementary Table 2 and Extended Data Fig. 2a). Notably, TS1 pigs displayed a high burden of arrhythmic mortality after birth, consistent with the lethality of the disease observed in humans^1,17^ (Extended Data Fig. 2b,c), but the QTc interval did not predict the occurrence of SCD in TS1 pigs. Subcutaneous implantable loop recorders (ILRs), which allow for continuous ECG monitoring, were implanted in *n* = 30 TS1 animals and documented the spontaneous occurrence of VF and SCD in 11 of 30 animals (37%) (Fig. 1c). Interestingly, we documented that VF episodes were preceded by premature ventricular contractions (PVCs), akin to what we observe in patients with TS1 (Fig. 1d).

**Extended Data Fig. 3:**
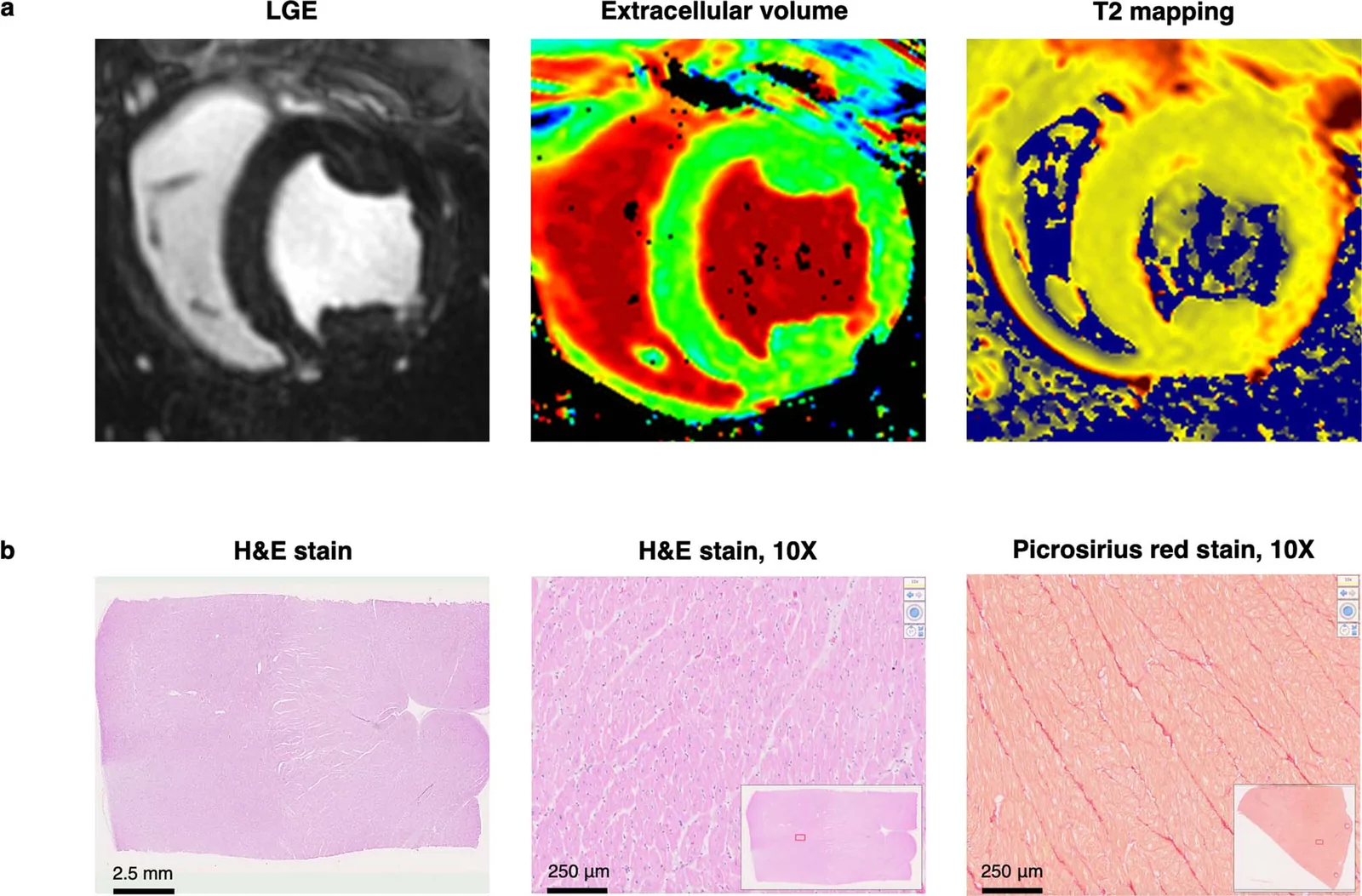
Applying advanced imaging and histological techniques for comprehensive cardiac tissue analysis in TS1 pigs. **a**, Tissue characterization with late gadolinium sequences (left panel), extracellular volume (central panel) and T2 mapping (right panel) did not show any signs suggestive of cardiac fibrosis or inflammation in N = 9 TS1 pigs. **b**, Histological samples from N = 12 TS1 pigs confirmed the absence of inflammatory infiltrates and absence of fibrosis.

Cardiac magnetic resonance (CMR) was performed to investigate for the potential presence of structural alterations of the myocardium (*n* = 10 WT and *n* = 9 TS1), demonstrating the presence of functionally and structurally normal hearts both in TS1 and WT animals (Extended Data Fig. 3a). Finally, the absence of inflammatory infiltrate and fibrosis was confirmed by histology (Extended Data Fig. 3b).

### In vivo electrophysiology study with EAM

After the demonstration that our TS1 model recapitulates the human disease’s cardinal features and is devoid of structural abnormalities that may promote arrhythmogenesis, we carried out in vivo EAM using two complementary clinical-grade EAM systems to investigate the arrhythmogenic substrate underlying life-threatening arrhythmias in TS1.

In the first part of the study, we used the NavX EnSite Velocity system to record and anatomically reconstruct the signals from the endocardial surfaces of both ventricles at 84 sites (64 in the left ventricle (LV) and 20 in the right ventricle (RV)) simultaneously, thus mapping in real time the electrical activity of both chambers in a single heartbeat (biventricular simultaneous EAM).

Biventricular simultaneous EAM during baseline conditions (that is, atrial pacing at a stable heart rate) allowed us to observe that, when TS1 pigs are compared to WT pigs, the time necessary for the propagation of cardiac activation (measured as local activation time (LAT)) was increased, which was an unexpected finding in TS1 (Supplementary Table 3). Departing from the usual clinical practice, we also investigated cardiac repolarization (measured as local recovery time (LRT)). Expectedly, the ventricular repolarization was significantly longer in TS1 pigs as compared to their WT counterparts. Relevantly, in baseline conditions, we did not document substantial differences between TS1 and WT in the dispersion of cardiac repolarization (measured as LRT range) (Supplementary Table 3).

**Fig. 2:**
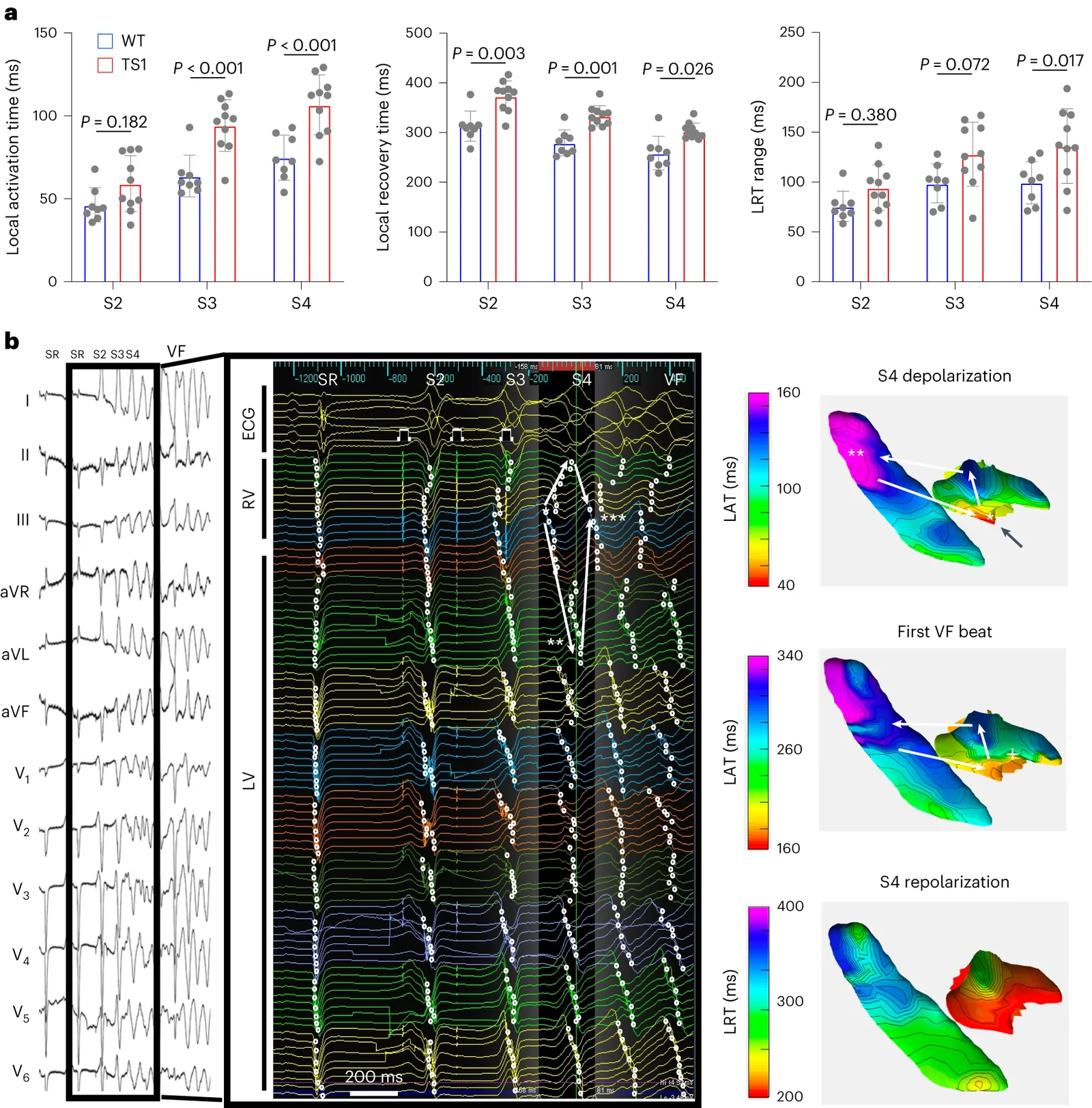
Biventricular simultaneous mapping and ventricular fibrillation induction. **a**, Graphs show the quantification of LAT, LRT and LRT range of the S2, S3 and S4 as close as possible to ventricular refractoriness during spontaneous sinus rhythm between WT (blue, *n* = 8) and TS1 (red, *n* = 10) animals. Each value is represented as mean ± s.d. Statistical analysis was conducted using two-way ANOVA with Šidák post tests. **b**, Surface ECG (left), unipolar electrograms (central) and electroanatomical maps (right) documenting ventricular fibrillation induction in a TS1 pig after three extrastimuli on sinus rhythm, a situation that never occurred in WT pigs. The first activation during S4 corresponded to the pacing site in the RV (*). The latest activated area was found in the LV (**), whereas the first spontaneously reactivating region was found in the proximal site in the RV (+). Notably, this early reactivated area (reentrant activity) corresponded to the area with the shortest LRT registered during the closest S4 that did not induce ventricular fibrillation. The black arrow indicates the pacing site in the RV endocardium. Source data

Prompted by the evidence from both patients and TS1 animals that one or more PVCs preceded the onset of VF (Fig. 1c,d), we used biventricular simultaneous EAM during administration of up to three premature ventricular extrastimuli (S2, S3 and S4, respectively) delivered on sinus rhythm to comprehend, in a controlled setting, the sequence of events culminating in the development of life-threatening arrhythmias. This approach highlighted that, in TS1 animals, as compared to WT counterparts, the delivery of successive premature ventricular extrastimuli with a progressively shorter coupling interval resulted in further exacerbation of delay of cardiac activation. The maximum impairment of impulse propagation was reached upon the delivery of the third premature ventricular extra stimulus (S4) (Fig. 2a and Supplementary Table 4). In parallel with the worsening delay of cardiac activation, we observed a gradual development of dispersion of repolarization, becoming evident after S4 (Fig. 2a and Supplementary Table 4).

Taken together, these results point out that the sequential introduction of three extrastimuli at progressively shorter coupling intervals significantly perturbates both activation and repolarization in TS1 hearts: the sequence of events suggests that the delivery of three extrastimuli very close to the effective refractory period leads to a significant delay in activation that is followed by an increase in the dispersion of repolarization, favoring an arrhythmogenic substrate prone to reentrant arrhythmias. This observation is at variance with previous evidence from pharmacological models of LQT3 (ref. ^18^), in which the functional conduction block was secondary to the infringement of the activation wavefront on the preexisting spatial dispersion of repolarization.

**Extended Data Fig. 4:**
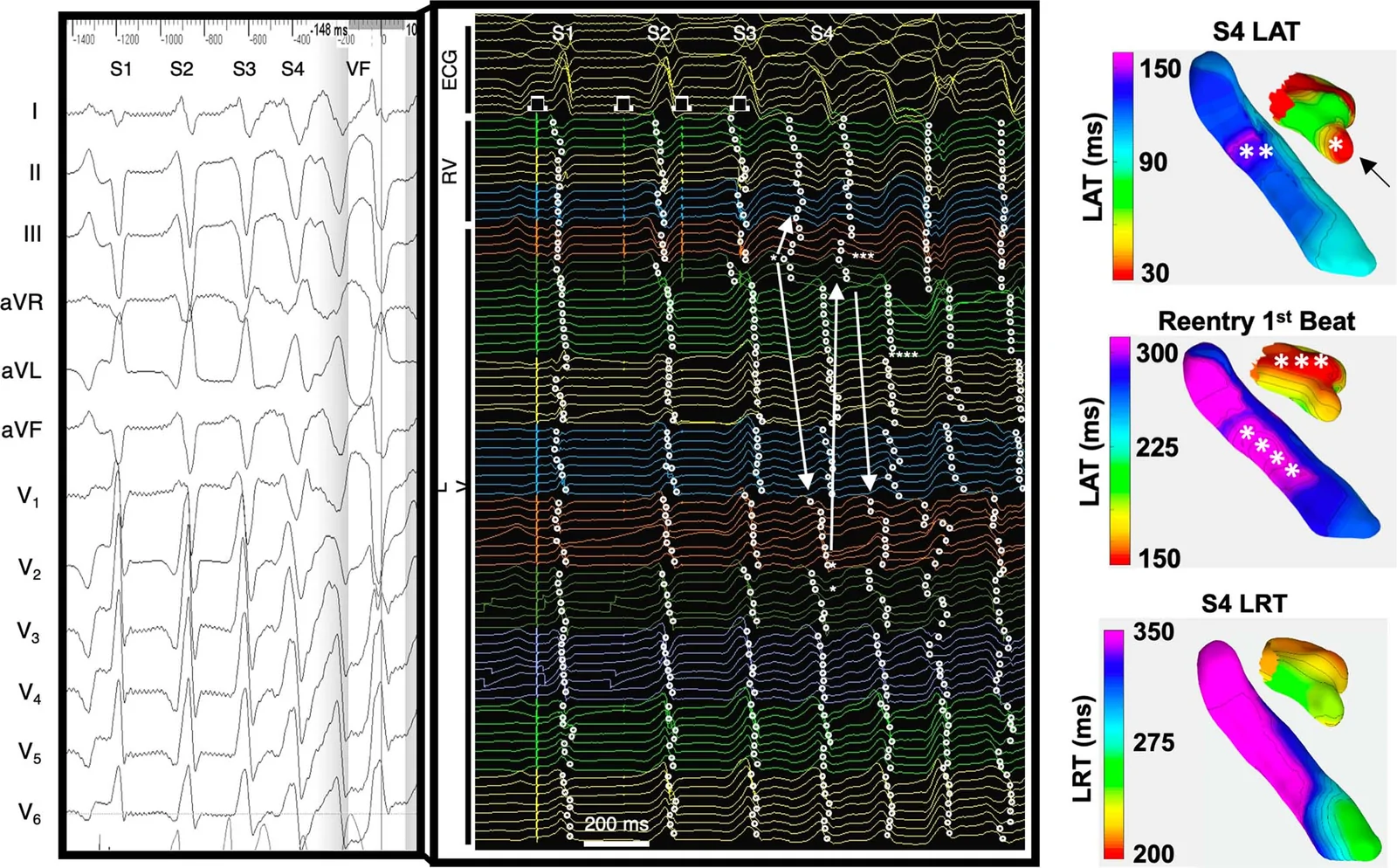
Second example of simultaneous biventricular mapping and ventricular fibrillation (VF) induction. Surface ECG (left), unipolar electrograms with local activation time depicted with white circles (central) and electroanatomical maps (right) documenting polymorphic VT induction in a TS1 pig after 3 extrastimuli. In analogy with Fig. 5 (see main text), the first activation during S4 corresponds to the pacing site in the right ventricle (*). Latest activated area was found in the left ventricle (**), while the first spontaneously reactivating region was found in the proximal site in the right ventricle (***), which corresponded to the area with the shortest LRT of the closest S4 that did not induce VF and was followed again by left ventricle activation (****). The black arrow indicates the pacing site in the RV endocardium.

To test the susceptibility to reentrant arrhythmias in our TS1 pigs, we performed programmed electrical stimulation that induced reentrant life-threatening arrhythmias in two of 10 TS1 pigs (Fig. 2b and Extended Data Fig. 4) and in none of the 10 WT animals. Interestingly, in the two inducible TS1 animals, life-threatening ventricular arrhythmias developed after the third premature ventricular extra stimulus (S4). The analysis of electrograms suggested the existence of a reentrant activity, a finding supported by the localization of the first reactivating region in the RV in correspondence with the area with the shortest repolarization time (Fig. 2b and Extended Data Fig. 4).

Considering the limited spatial resolution of simultaneous EAM, we decided to investigate more precisely the electrophysiologic substrate at its greatest extent (that is, during delivery of S4 on sinus rhythm) using the RHYTHMIA HDx Mapping System (Boston Scientific) that allows the reconstruction of anatomically accurate, high-density maps obtained with the sequential sampling of numerous endocardial and epicardial sites with 64-pole catheter (high-density sequential EAM). We exploited the high density of signals collected to calculate advanced metrics, such as conduction velocity (CV), repolarization gradients (LRT gradients) that express the spatial dispersion of repolarization and the reentry vulnerability index (RVI), a clinically validated parameter defined as the interval between the repolarization time at a site proximal to a line of conduction block and the activation time at an adjacent site^19^. This spatiotemporal parameter quantifies the propensity of the electrical substrate to reentrant arrhythmias: the lower the RVI, the more vulnerable the heart becomes to the development of reentrant arrhythmias^19^. To offer a clinically relevant parameter that permits direct comparisons between different subjects, we also calculated the global RVI (RVI_G,D_), a cardiac cycle length-corrected index that identifies the greatest predisposition to reentrant arrhythmias^20^.

**Fig. 3:**
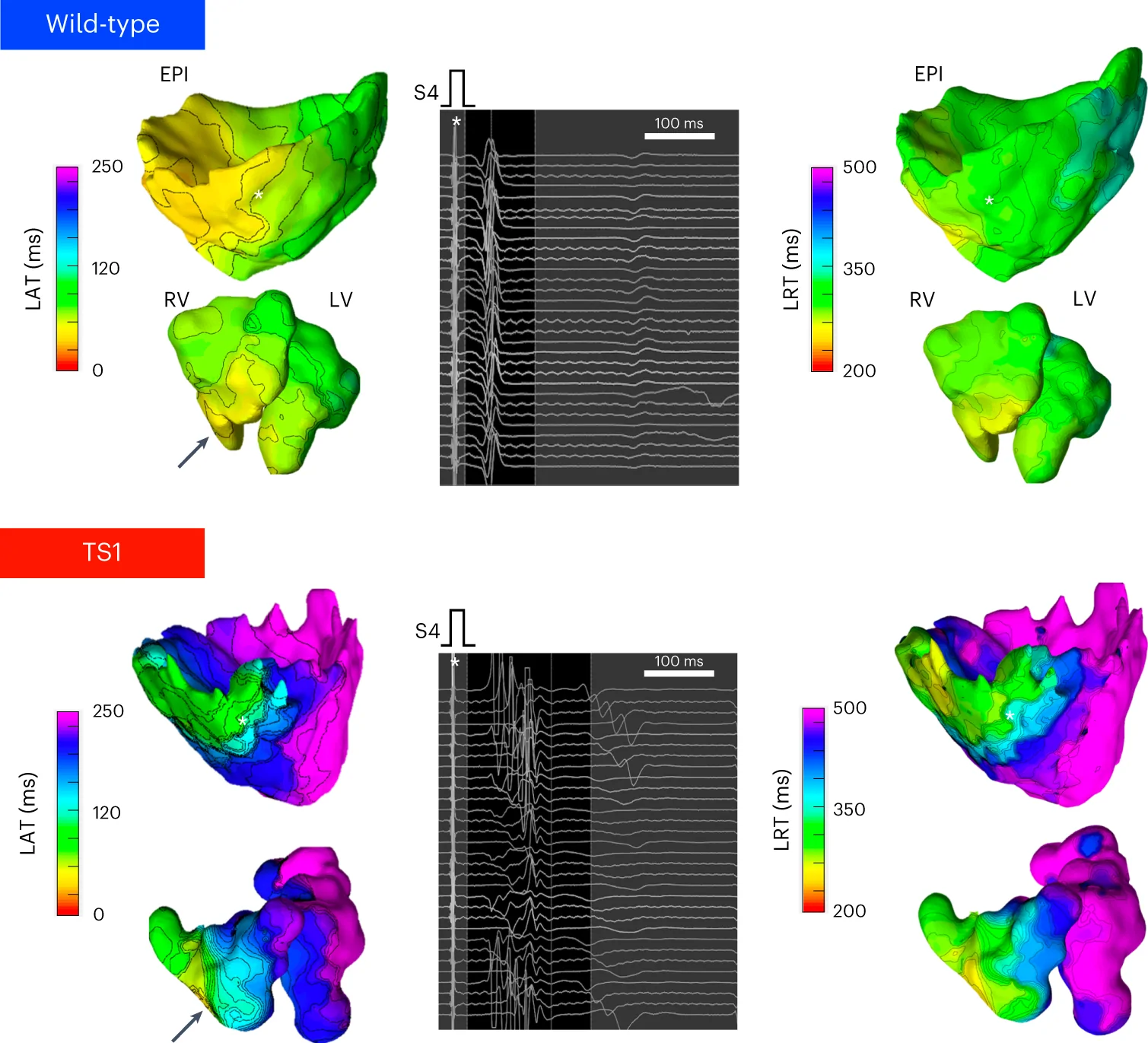
Ultra-high-density sequential EAM discloses the pathological functional substrate in the TS1 swine model. Electroanatomical endo-epicardial maps and exemplificative unipolar electrograms (*) of a WT animal (top) and a TS1 animal (bottom) are represented. Compared to the WT, three premature stimuli caused a marked activation delay in TS1 (bottom left), whereas the LRT abbreviated heterogeneously, creating an increased dispersion of repolarization, as compared to WT (top right). Black arrows indicate the pacing site in the RV endocardium.

**Fig. 4:**
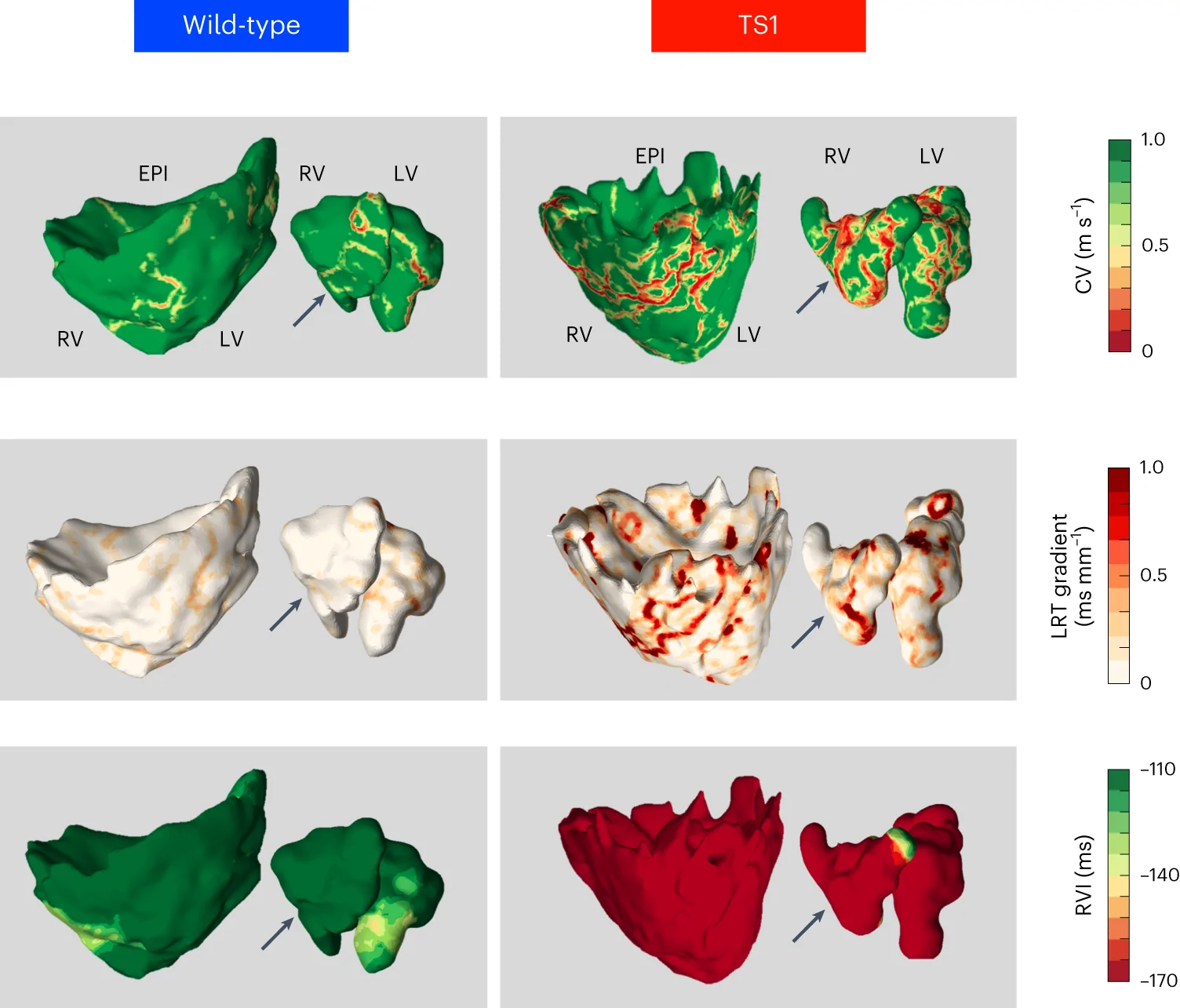
Advanced mapping metrics obtained from ultra-high-density sequential EAM confirmed the presence of a substrate vulnerable to reentry in the TS1 swine model. Compared to WT (upper left), three premature stimuli caused the appearance of areas of slow conduction (CV <0.2 m s^−1^) in TS1 (upper right). In correspondence with such areas, the LRT abbreviates heterogeneously, as demonstrated by steep LRT gradients (central right), creating a substrate vulnerable to the development of functional reentry, as documented by extremely low RVI (lower right). Black arrows indicate the pacing site in the RV endocardium.

The high-density sequential EAM during S4 confirmed that the cardiac activation was twice as long in TS1 animals as compared to WT littermates (Fig. 3 and Supplementary Table 5) and permitted the documentation of distinct areas of conduction block (defined as local CV < 0.2 m s^−1^ (ref. ^21^)) that were six times larger in TS1 pigs (Fig. 4 and Supplementary Table 5). In line with what we documented using biventricular simultaneous EAM, we demonstrated that, in TS1 animals, the repolarization was not only pathologically prolonged but was also significantly dispersed with steep gradients (Fig. 4 and Supplementary Table 5). Considering that neither the areas of conduction block nor the areas of dispersed repolarization, elicited by S4, were confined to a specific anatomical area, it is likely that the co-development of the aforementioned components of arrhythmogenesis predisposed to the occurrence of functional reentry, which we previously documented.

Because establishing a reentrant arrhythmia depends on the dynamic, spatiotemporal interplay of activation delay and dispersion of repolarization, we created RVI maps (Fig. 4), and we used global RVI to quantify the vulnerability to reentrant arrhythmias. Our data showed that, in TS1 pigs, numerous anatomically unrelated sites were permissive to reentry (Fig. 4), and, overall, TS1 pigs had a greater susceptibility to reentrant arrhythmias than WT, as confirmed by a 76% lower global RVI (Supplementary Table 5).

Overall, EAM of knock-in TS1 swine showed that the propagation of cardiac activation in mutant animals is slower than in WT animals despite the absence of pathological structural abnormalities, such as cardiac fibrosis, suggesting the presence of a constitutional, functional and often latent arrhythmogenic substrate. These data lead to the interpretation that the occurrence of PVCs, which act as an arrhythmogenic trigger, exacerbate the defect of cardiac activation, generating the arrhythmic milieu permissive for the occurrence functional reentrant circuits that initiate ventricular arrhythmias rapidly degenerating into VF.

### EAM as a tool for the assessment of antiarrhythmic efficacy

In the three most common forms of LQTS, called LQT1, LQT2 and LQT3, the greater the prolongation of the QTc interval, the higher the arrhythmic risk^22–24^. However, in TS1, to our knowledge, the duration of QTc interval has not been demonstrated to be a predictor of arrhythmic risk. Data collected in the present study suggest that parameters that can be acquired using endocardial and epicardial mapping may provide additional information about the arrhythmic substrate, such as the global RVI^20^. Consequently, we speculated that high-density EAM in TS1 may provide a fingerprint of the individual’s arrhythmic substrate and that, potentially, it might help in the assessment of efficacy of anti-arrhythmic therapies.

To test this hypothesis, we leveraged on the availability of our TS1 model, which represents a unique tool for drug testing, and we used several different compounds. First, we examined compounds that have been tested in patients with TS1: the sodium channel blockers mexiletine^4^ and ranolazine^25^ and the calcium channel blocker verapamil, administered in isolation or combined with the selective beta blocker metoprolol^26^. We also tested two preclinically investigated compounds: dextromethorphan, a sigma non-opioid intracellular receptor 1 agonist shown to restore the inactivation of Ca_V_1.2 channel in iPSC-derived cardiomyocytes from a patient with TS1 (ref. ^14^), and 3-nitro-N-(4-phenoxyphenyl)benzamide (ICA-105574)^27^, an hERG channel agonist shown to increase IKr current, thus abbreviating the ventricular repolarization^28–30^.

**Fig. 5:**
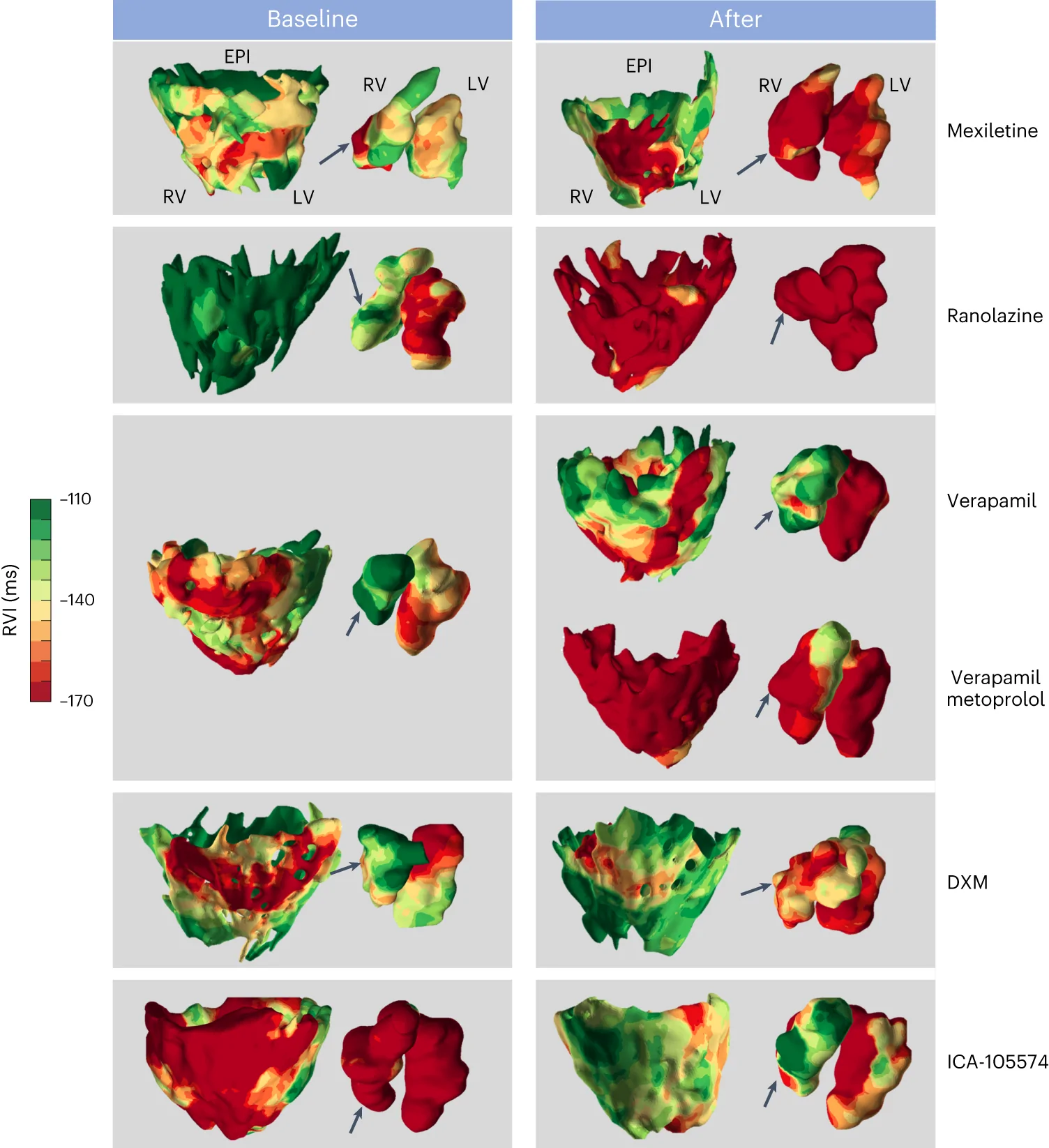
RVI assessment in a TS1 model after administration of various pharmacological compounds, visualized using high-density EAM. This figure presents representative RVI maps for the same TS1 pig before (left panels) and after (right panels) pharmacological interventions. The color-coded regions, green and red, represent areas with a lower and a higher likelihood of reentrant arrhythmia, respectively, as per the RVI evaluation. Administration of sodium channel blockers mexiletine and ranolazine exacerbated the arrhythmic substrate, as indicated by a worsening of the global RVI. Conversely, verapamil administration enhanced the uniformity of cardiac activation and repolarization, leading to a slight improvement in the global RVI. However, the verapamil-induced benefits were reversed by the concomitant administration of metoprolol, a selective beta blocker. Administration of dextromethorphan (DXM) resulted in more homogeneous cardiac repolarization, but the effects on cardiac activation were minimal, thus resulting in only minor changes in the arrhythmic substrate, as evidenced by negligible changes in RVI. Finally, administration of ICA-105574 resulted in a significant reduction in activation delay compared to baseline. In conjunction with homogenization of the spatial dispersion of repolarization, this resulted in a positive effect evidenced by improved RVI (lower-right panel), suggesting a substrate less prone to reentry after ICA-105574 administration. Black arrows indicate the pacing site in the RV endocardium. Comprehensive pooled data on RVI and other electrophysiological parameters are provided in Supplementary Table 6.

Interestingly, although both ranolazine and mexiletine shortened the duration of the QTc interval, EAM revealed that they both caused a further worsening in the delay in cardiac activation and a further dispersion of repolarization as compared to baseline, translating into a net worsening of the arrhythmic substrate as testified by global RVI worsening (Fig. 5 and Supplementary Table 6). Of relevance, during one of the experiments with ranolazine, we documented the induction of VF during EAM on S4, which was refractory to defibrillation and resuscitation maneuvers.

The calcium channel blocker verapamil induced a marked shortening of the QTc interval but differently from sodium channel blockers. This translated into an amelioration of the cardiac activation delay and homogenization of repolarization, resulting in an overall improvement of global RVI (Fig. 5 and Supplementary Table 6). However, when verapamil was combined with the selective beta blocker metoprolol, the verapamil-induced benefits were lost, such that, as compared to baseline, there were no substantial differences, corroborated by the nearly unchanged global RVI (Fig. 5 and Supplementary Table 6).

We then focused on the two preclinical compounds, dextromethorphan and ICA-105574. Consistent with published data from the mouse model of TS1 published by Song et al.^14^, we found that administering dextromethorphan to our TS1 pigs led to a significant shortening of the QTc interval. Although the use of dextromethorphan was not associated with substantial modification of the cardiac activation, it resulted in homogenization of repolarization (Fig. 5 and Supplementary Table 6). On the other hand, the administration of ICA-105574, besides causing a prominent shortening of the QTc interval, resulted in a reduction of the activation delay as compared to baseline (Fig. 5 and Supplementary Table 6). This, coupled with the homogenization of the spatial dispersion of repolarization, had a net positive effect, as evidenced by an amelioration by 30% of global RVI (Supplementary Table 6).

Our data suggest that endo-epicardial EAM performed during introduction of premature beats may be useful for the understanding of arrhythmic substrate and that the repetition of EAM during perturbation of sinus rhythm with premature beats after initiation of drug treatment may provide additional information about the efficacy of therapies that, besides shortening the QT interval, might exert complex and unpredictable effects on the arrhythmogenic substrate of the disease.

### Phenotypic signature of TS1 pigs

After the dissection of the organ-level mechanisms of arrhythmogenesis, we turned to cellular investigations performed in isolated ventricular cardiomyocytes to elucidate the molecular (mal)adaptive mechanisms that underlie the arrhythmogenesis in our porcine TS1 model.

**Fig. 6:**
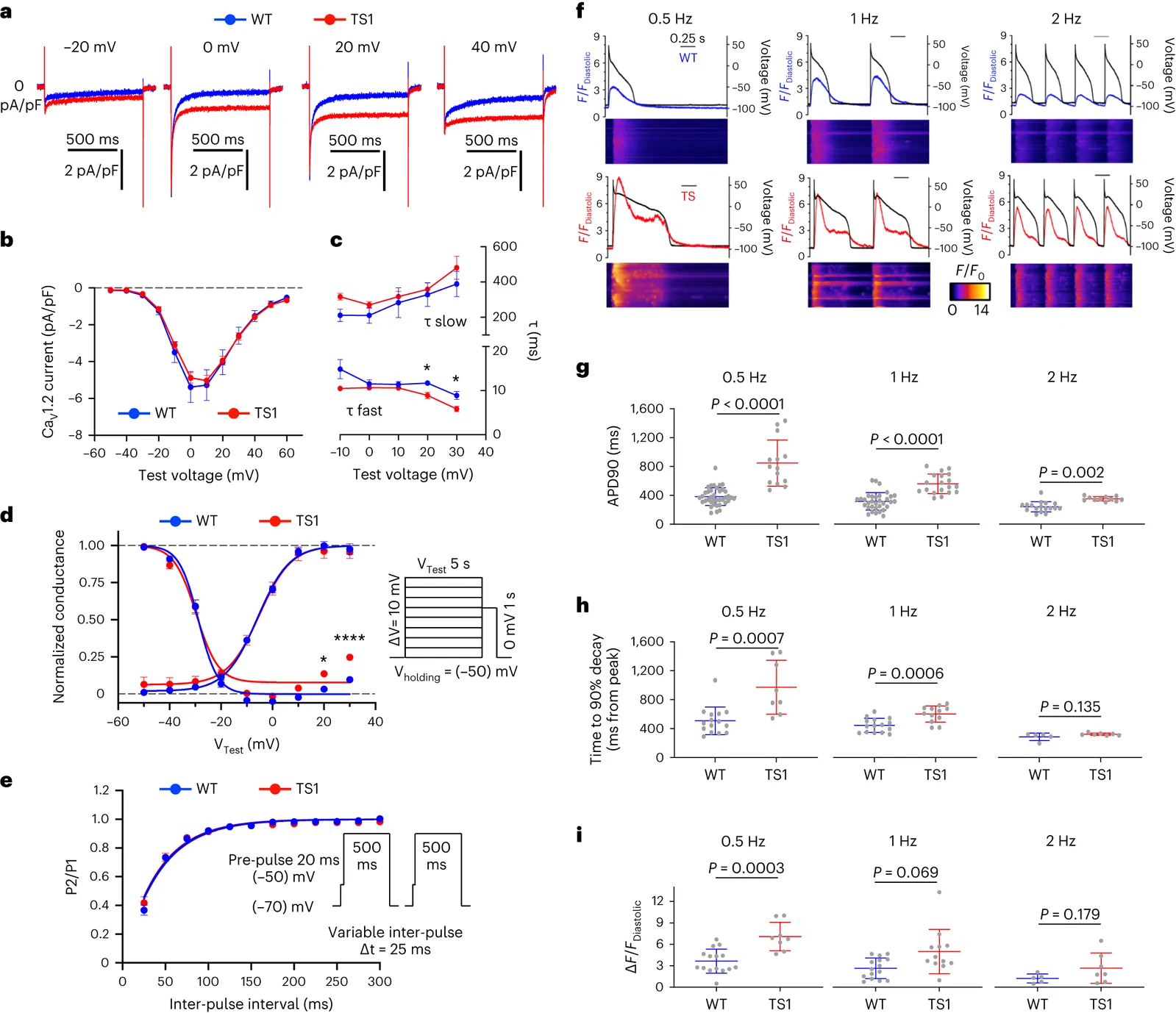
Cellular phenotype of TS1 cardiomyocytes. **a**, Representative traces (20-mV increments from −20 mV) of I_Ca_ in WT (blue) and TS1 (red) cardiomyocytes. From those measurements, peak I-V curve and speed of inactivation were calculated. **b**, Peak I_Ca_ I-V relationships for WT (blue, *n* = 22 cells from *N* = 5 animals) and TS1 (red, *n* = 15 cells from *N* = 3 animals) (*P* = 0.825). **c**, Analysis of I_Ca_ inactivation rates in WT (blue, *n* = 21 cells from *N* = 5 animals) and TS1 (red, *n* = 15 cells from *N* = 3 animals). For τ fast: *P* = 0.020 and *P* = 0.047 at +20 mV and +30 mV, respectively. **d**, Activation and availability curves of I_Ca_ in WT (blue, *n* = 14 cells from *N* = 3 animals) and TS1 (red, *n* = 20 cells from *N* = 3 animals) (*P* = 0.010 and *P* < 0.0001 at +20 mV and +30 mV, respectively). **e**, Recovery from inactivation of I_Ca_ for WT (blue, *n* = 15 cells from *N* = 3 animals) and TS1 (red, *n* = 18 cells from *N* = 3 animals) (*P* = 0.300). **f**, Representative traces of AP and simultaneous Ca^2+^ transient recordings at 0.5 Hz, 1 Hz and 2 Hz in WT (top) and TS1 (bottom) isolated cells. **g**, Quantification of APD90 at 0.5 Hz, 1 Hz and 2 Hz in WT (*n* = 38/33/17 cells from *N* = 14/14/10 animals, respectively) and TS1 (*n* = 14/19/11 cells from *N* = 6/7/5 animals, respectively). **h**, Quantification of the time to 90% decay (from peak amplitude) of Ca^2+^ transient at 0.5 Hz, 1 Hz and 2 Hz in WT (*n* = 15/15/5 cells from *N* = 5/8/3 animals, respectively) and TS1 (*n* = 8/12/7 cells from *N* = 4/6/5 animals, respectively). **i**, Quantification of Ca^2+^ transient amplitude at 0.5 Hz, 1 Hz and 2 Hz in WT (*n* = 15/15/5 cells from *N* = 5/8/3 animals) and TS1 (*n* = 8/12/7 cells from *N* = 4/6/5 animals). Each value is represented as mean ± s.e.m. (**b**–**e**) or mean ± s.d. (**g**–**i**). Statistical analyses were conducted using two-way ANOVA with Šidák post test (**b**–**e**) and two-tailed nested *t*-test (**g**–**i**). NS, not significant, *P < 0.05, **P < 0.01, ***P < 0.001 and ****P < 0.0001. Source data

**Extended Data Fig. 5:**
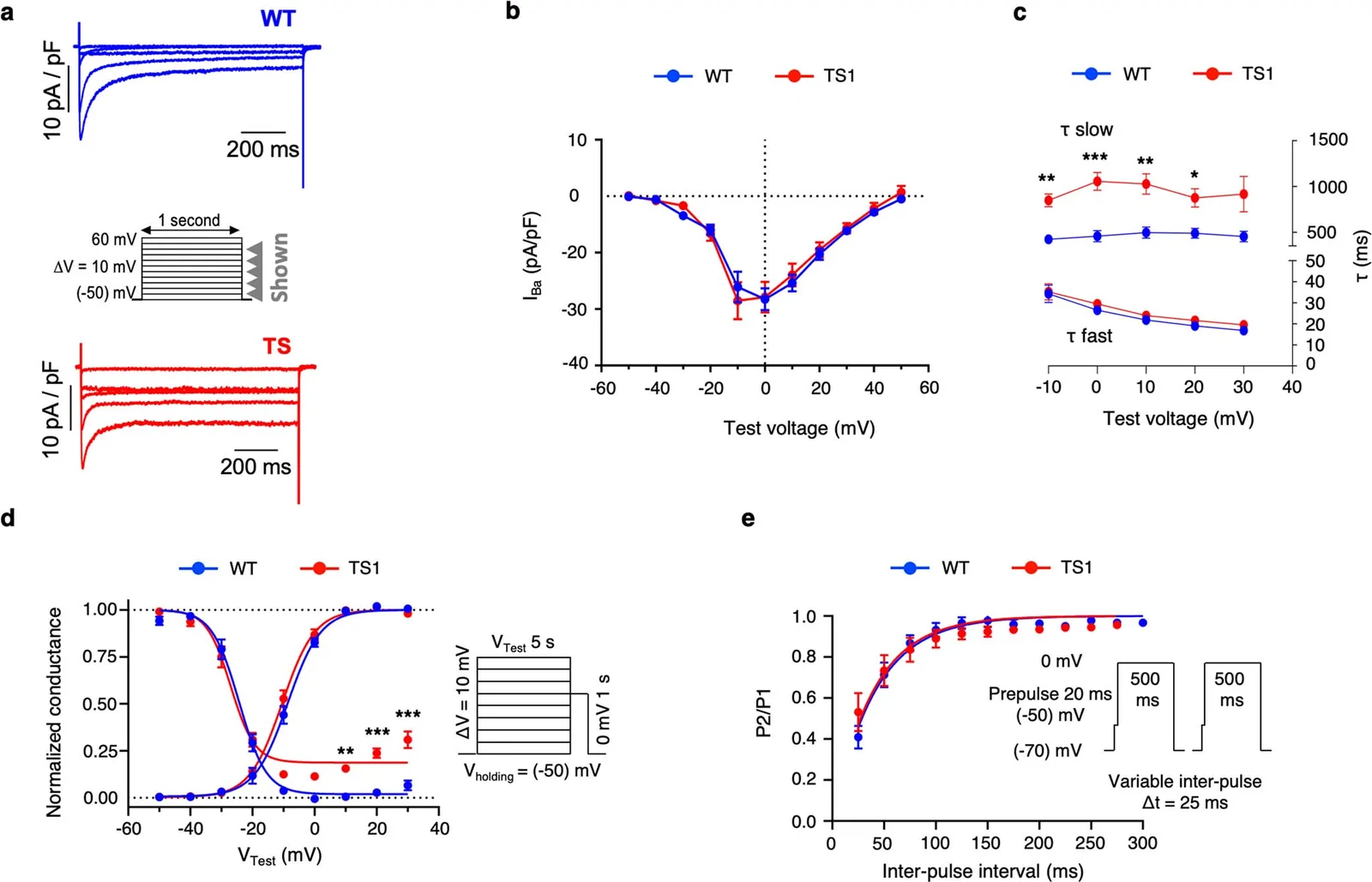
I_Ba_ measurements in TS1 and wild-type ventricular myocytes. **a**, Representative I_Ba_ traces in WT (blue) and TS1 (red) cardiomyocytes at the indicated test voltages (left section; shown each 20 mV from -40 mV, gray arrows). From those measurements, the peak I-V curve and the speed of inactivation were calculated. **b**, Peak I_Ba_ I-V relationships for WT (blue, *n* = 9 cells from N = 5 animals) and TS1 (red, *n* = 8 cells from N = 4 animals) (p = 0.817). **c**, Analysis of I_Ba_ inactivation rates in WT (blue, *n* = 10 cells from N = 5 animals) and TS1 (red, *n* = 9 cells from N = 4 animals). For τ slow: p = 0.001, p = 0.001, p = 0.006 and p = 0.030 at -10 mV, 0 mV, +10 mV, and +20 mV, respectively. **d**, Activation and availability curves of I_Ba_ in WT (blue, *n* = 11 cells from 3 animals) and TS1 (red, *n* = 9 cells from 4 animals). p = 0.004, p < 0.0001 and p < 0.0001 at +10 mV, +20 mV and +30 mV, respectively. **e**, Recovery from inactivation of I_Ba_ for WT (blue, *n* = 5 cells from 2 animals) and TS1 (red, *n* = 2 cells from 1 animal) (p = 0.630). Each value is represented as mean ± s.e.m. (**b-e**). Statistical analyses were conducted using two-way ANOVA with Šidák post-test (**b-e**). N.S. not significant, *p < 0.05, **p < 0.01, ***p < 0.001, ****p < 0.0001. Source data

As a first step of in vitro phenotyping, we characterized I_Ca_, the current primarily affected by the p.Gly406Arg mutation in TS1 ventricular cardiomyocytes (Fig. 6a). Our data showed that peak Ca^2+^ currents were similar between phenotypes (Fig. 6b), whereas the fast time constant of inactivation was smaller in TS1 cardiomyocytes (Fig. 6c), suggesting a larger local sarcoplasmic reticulum (SR) Ca^2+^ release with subsequent Ca^2+^-dependent inactivation, as previously published^7^. Also, we confirmed the impairment of Ca_V_1.2 inactivation in TS1 (ref. ^1^), manifested as increased steady-state availability (Fig. 6d). Additional experiments using 5 mM Ba^2+^ as charge carrier were used to isolate VDI, confirming its impairment in TS1, which is manifested as a slower time constant of the slow component of inactivation^7^ (Extended Data Fig. 5). No differences in activation (Fig. 6d) or in recovery from inactivation (Fig. 6e) were found among phenotypes, neither for I_Ca_ nor for I_Ba_ (Extended Data Fig. 5).

**Fig. 7:**
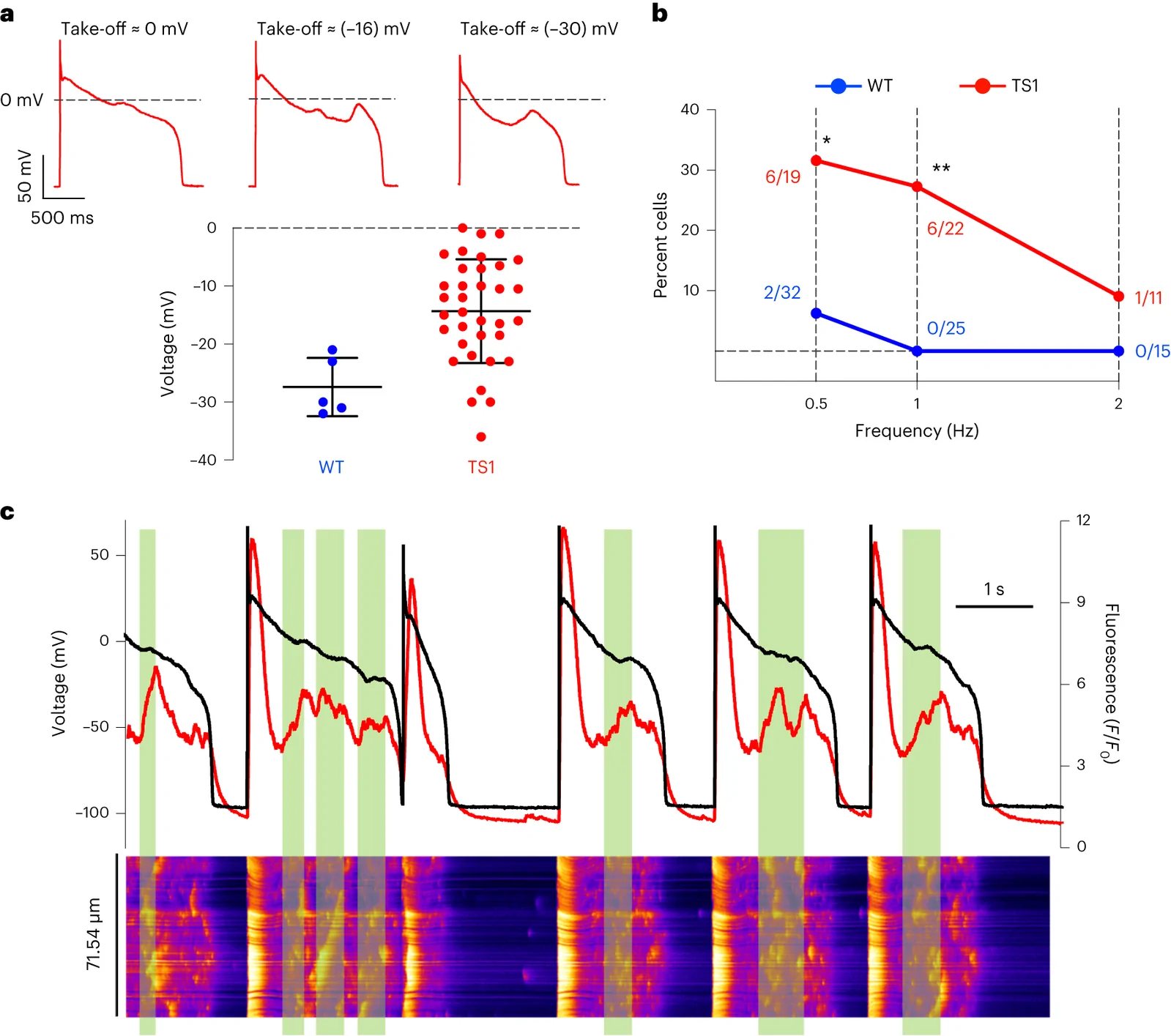
EADs in TS1 and potential contribution of late-systolic Ca^2+^ waves/ripples to EAD genesis and maintenance. **a**–**c**, EADs in TS1. **a**, Representative examples of EADs with different take-off potentials in TS1 cardiomyocytes and quantification of take-off potentials of EADs in WT (blue, *n* = 5 EADs from *n* = 2 cells from *N* = 2 animals) and TS1 (red, *n* = 35 EADs from *n* = 11 cells from *N* = 9 animals) cells paced at 0.5 Hz, 1 Hz or 2 Hz (*P* = 0.159). Each value is represented as mean ± s.d. Statistical analysis was conducted using two-tailed nested *t*-test. **b**, Quantification of the incidence of EADs in WT (blue, *n* = 32/25/15 cells from *N* = 14/14/10 animals) and TS1 (red, *n* = 19/22/11 cells from *N* = 6/7/5 animals) cells paced at 0.5 Hz, 1 Hz or 2 Hz (*P* = 0.040, *P* = 0.007 and *P* = 0.423 at 0.5 Hz, 1 Hz and 2 Hz, respectively). Each dot in the graph represents the percentage of cells with EADs. Statistical analyses were conducted using two-tailed chi-square with Fisher exact test. NS, not significant, *P < 0.05, **P < 0.01, ***P < 0.001 and ****P < 0.0001. **c**, Simultaneous voltage (black line) and global Ca^2+^ recordings (red line, confocal image) from a TS1 cell presenting EADs at 0.5 Hz. The figure highlights (green fringes) time periods during which the AP experiences an oscillation and during which fusion of late-systolic sparks leads to a global rise in Ca^2+^. Source data

**Extended Data Fig. 6:**
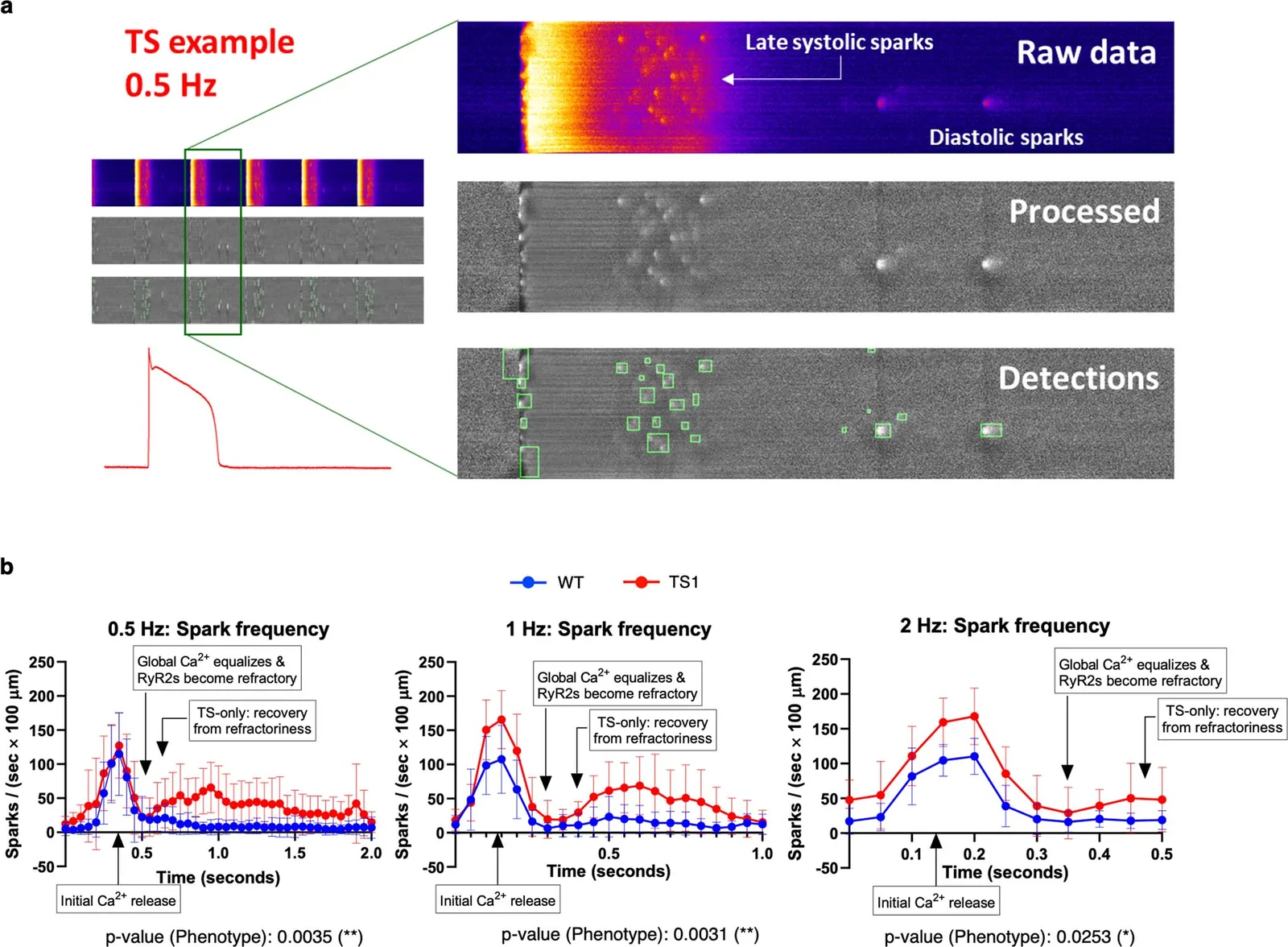
Late-systolic sparks (during the AP) and diastolic Ca^2+^ sparks in TS1 cells. **a**, Raw confocal images were pre-processed to correct for the effects of background Ca^2+^ and inhomogeneities in the Rhod 2 distribution. These pre-processed images were subjected to an in-home spark detection algorithm (detected events are shown as green rectangles). Also shown (bottom-left) is an action potential corresponding to the beat zoomed in a green square. The sharp membrane repolarization ends the occurrence of late-systolic sparks. **b**, Temporal evolution of the spark frequency for all cells studied in WT (blue, *n* = 14/13/5 cells from N = 6/8/3 animals, at 0.5, 1, and 2 Hz, respectively) and TS1 (red, *n* = 8/12/7 cells from N = 4/6/6 animals at 0.5, 1, and 2 Hz, respectively). Complete analysis procedure for an individual cell: For each cell analyzed, (1) sparks were detected as described in panel a, (2) spark frequency was binned in 50 ms intervals, (3) spark frequency bins were aligned at the beginning of each beat (0.5, 1 or 2 Hz), and (4) bins were averaged, thus yielding the temporal evolution of the spark frequency on an individual cell basis. Statistical analyses were conducted using two-way ANOVA. N.S. not significant, *p < 0.05, **p < 0.01, ***p < 0.001, ****p < 0.0001. Source data

**Extended Data Fig. 7:**
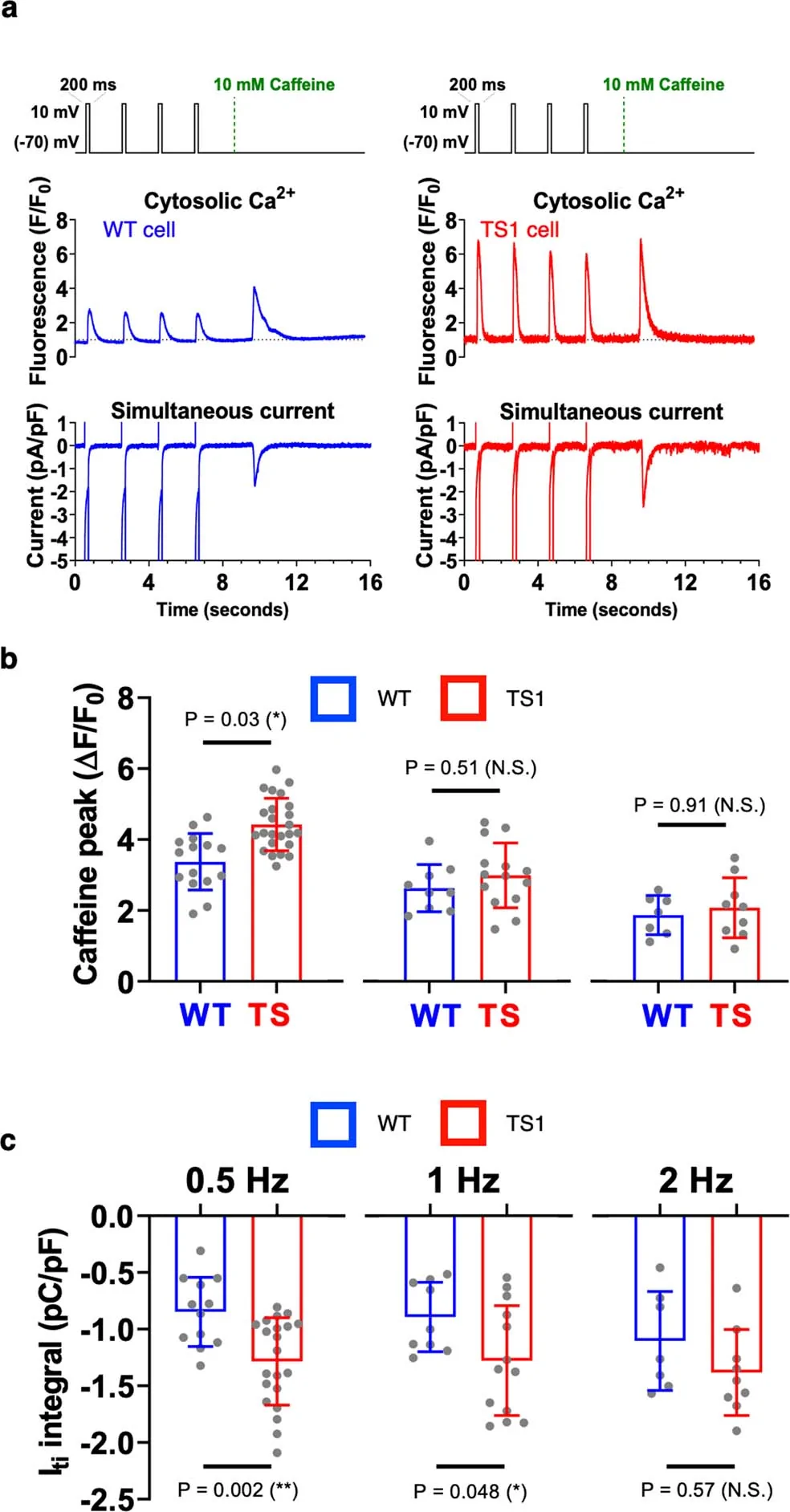
Increased SR Ca^2+^ content in TS1 ventricular myocytes. **a-b**, SR Ca^2+^ content measured by two simultaneous methods: caffeine-induced Ca^2+^ release and its concomitant Transient Inward current, a reliable indicator of the efflux of the released Ca^2+^ by the Na^+^/Ca^2+^ exchanger. Both methods demonstrate that the SR Ca^2+^ content is higher in TS1 (red) as compared to WT (blue) cardiomyocytes. **a**, SR Ca^2+^ content measurements (shown 1 Hz). **b**, Pooled data of caffeine peak in WT (blue, *n* = 15/9/7 cells from N = 3/3/3 animals, at 0.5, 1, and 2 Hz, respectively) and TS1 cardiomyocytes (red, *n* = 24/14/9 cells from N = 4/4/3 animals at 0.5, 1, and 2 Hz, respectively). **c**, Pooled data of Transient Inward current time-integral in WT (blue, *n* = 12/9/7 cells from N = 3/3/3 animals, at 0.5, 1, and 2 Hz, respectively) and TS1 cardiomyocytes (red, *n* = 21/13/9 cells from N = 4/4/3 animals at 0.5, 1, and 2 Hz, respectively). Each value is represented as mean ± s.e.m. Statistical analyses were conducted using two-tailed nested *t*-test. N.S. not significant, *p < 0.05, **p < 0.01, ***p < 0.001, ****p < 0.0001. Source data

This impairment of VDI of the Ca_V_1.2 channel corresponded to a significant prolongation of action potential (AP) (Fig. 6f,g and Supplementary Table 7) and an increase in the duration and amplitude of the Ca^2+^ transients (Fig. 6f,h,i and Supplementary Table 7). Interestingly, TS1 cells also presented a late plateau of continuous SR Ca^2+^ release in the form of increased frequency of late systolic sparks (Extended Data Fig. 6). In some cells, late systolic sparks coalesced into systolic Ca^2+^ ripples/waves, which correlated in time with the occurrence of early after-depolarizations (EADs; Fig. 7a–c). Our observation, in the context of TS1, is consistent with previous reports^31,32^ linking late systolic Ca^2+^ waves to EAD generation and maintenance, via I_ti_ current (Extended Data Fig. 7). These data support the hypothesis that the reentry-generating PVCs are induced by EADs.

**Extended Data Fig. 8:**
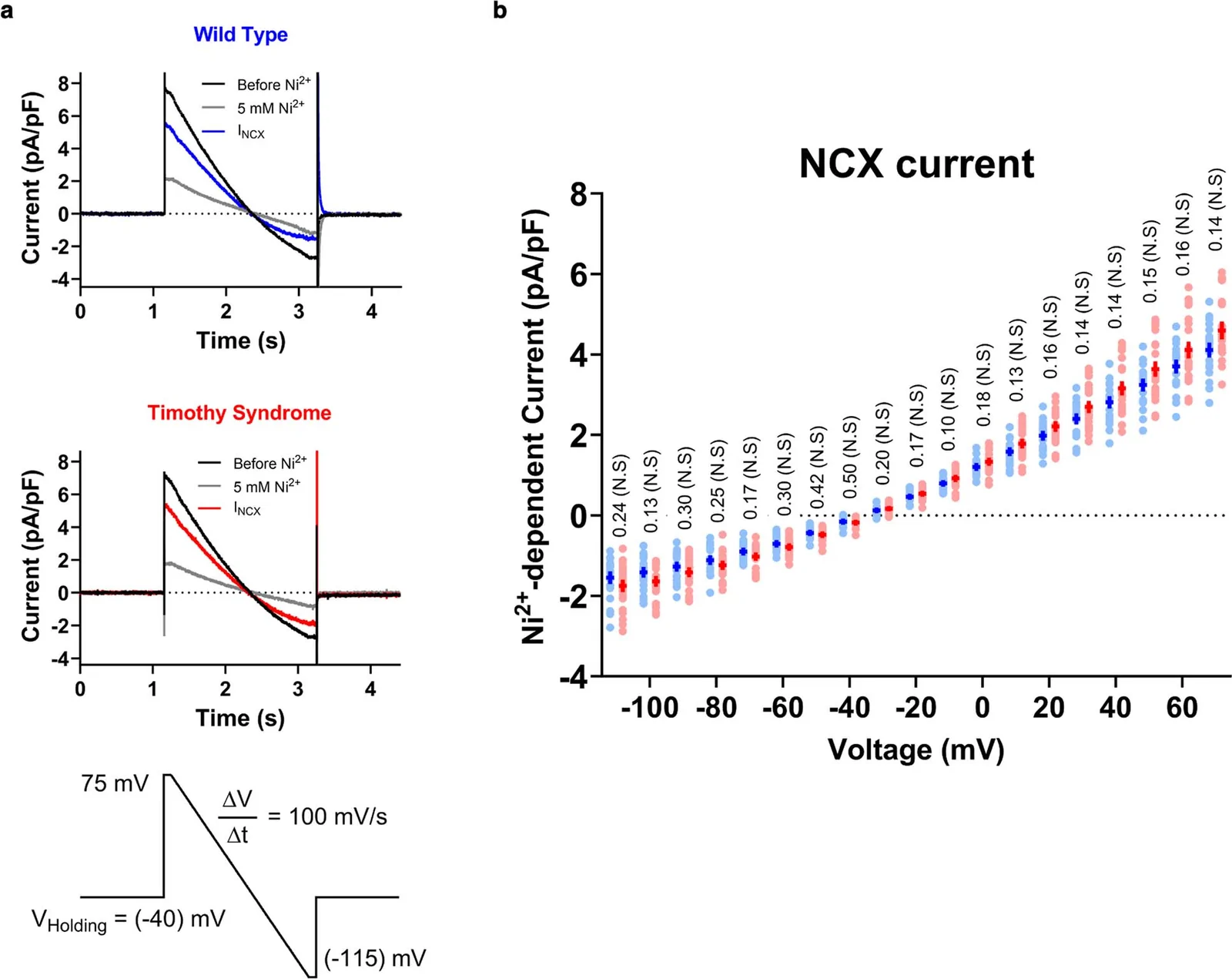
NCX current comparison in cardiomyocytes from wild-type and TS1 pigs. **a**, NCX currents were recorded in isolated WT (top-left; blue) and TS1 (center-left; red) ventricular cardiomyocytes, in response to the voltage protocol shown below (bottom-left). Currents were first recorded in the absence (black traces) and then in the presence (grey traces) of 5 mM Nickel, a NCX inhibitor. Thus, NCX currents were obtained by subtraction (blue trace at the top-left panel and red trace at the center-left panel). **b**, I-V curve of the pooled data of WT (top-left; blue, *n* = 21 cells from N = 5 animals) and TS1 (center-left; red, *n* = 25 cells from N = 5 animals) cardiomyocytes, represented at 10 mV intervals. Each value is represented as mean ± s.e.m. Statistical analyses were conducted using two-tailed nested *t*-test (p values shown in the figure for each 10 mV interval). N.S. not significant, *p < 0.05, **p < 0.01, ***p < 0.001, ****p < 0.0001. Source data

We then sought to investigate if the larger amplitude of Ca^2+^ transients and the increased frequency of late systolic Ca^2+^ sparks documented in TS1 were secondary to an increased SR Ca^2+^ content, as previously reported in mouse models of TS1 (ref. ^7^). To this end, we investigated the SR Ca^2+^ content using caffeine-induced Ca^2+^ release and assessed the concomitant transient inward current (I_ti_), a reliable indicator of the efflux of the released Ca^2+^ by the Na^+^/Ca^2+^ exchanger (NCX)^33^. Both approaches rendered that the SR Ca^2+^ content was indeed higher in TS1 (Extended Data Fig. 7), but the NCX current density was not affected (Extended Data Fig. 8).

Thus, it would appear that the slowed voltage-dependent inactivation caused by the p.Gly406Arg mutation and the secondary increase in SR Ca^2+^ content, which would drive fusion of late systolic sparks into ripples/waves (thus enhancing depolarizing NCX), may contribute both to EAD generation and/or maintenance in TS1. Of relevance, despite Ca^2+^ overload, we did not document the occurrence of delayed after-depolarizations (DADs) in our TS1 model.

**Extended Data Fig. 9:**
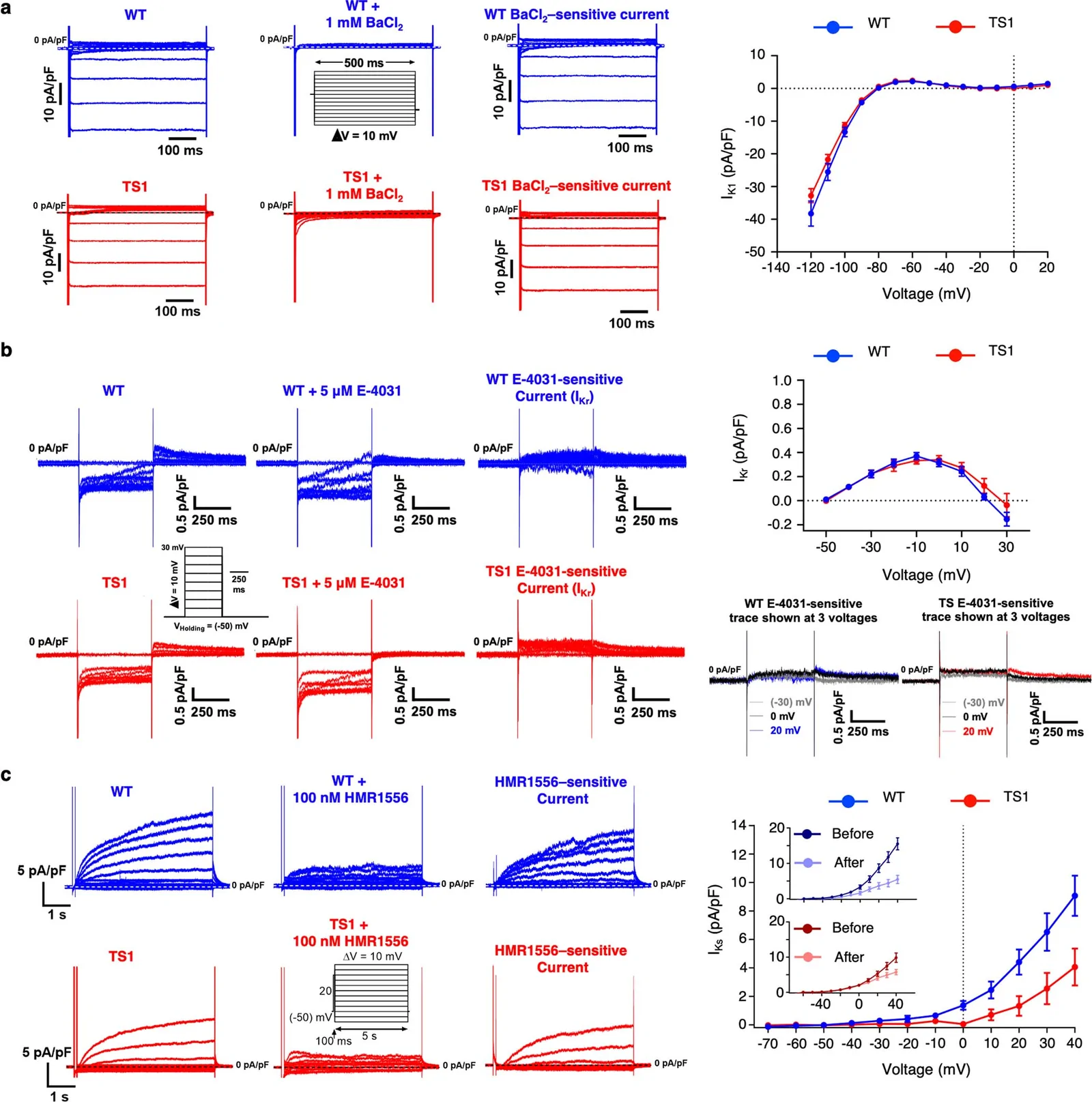
Comparison of potassium currents between TS1 and wild-type cardiomyocytes. **a-c**, The K^+^ currents I_K1_, I_Kr_ and I_Ks_ were measured as, respectively, barium-, E-4031- and HMR-1556-sensitive currents, at the indicated concentrations. **a**, Pooled data of I_K1_ current in WT (blue, *n* = 12 cells from N = 3 animals) and TS1 cardiomyocytes (red, *n* = 13 cells from N = 3 animals) showed no significant differences (p = 0.275). **b**, Pooled data of I_Kr_ current in WT (blue, *n* = 12 cells from N = 3 animals) and TS1 cardiomyocytes (red, *n* = 13 cells from N = 3 animals) showed no significant differences (p = 0.555). The insets shown below the I_Kr_ I-V highlight 3 voltages in the WT and TS E-4031-sensitive traces. **c**, Pooled data of I_Ks_ current in WT (blue, *n* = 10 cells from N = 2 animals) and TS1 cardiomyocytes (red, *n* = 11 cells from N = 2 animals) showed a significant reduction in current in TS1 cardiomyocytes (p = 0.008). The insets shown within the I_Ks_ I-V (before-after drug) show the loss of outward current in response to HMR-1556 treatment. Each value is represented as mean ± s.e.m. (**a-c**). Statistical analyses were conducted using two-way ANOVA with Šidák post-test (**a-c**). N.S. not significant, *p < 0.05, **p < 0.01, ***p < 0.001, ****p < 0.0001. Source data

**Extended Data Fig. 10:**
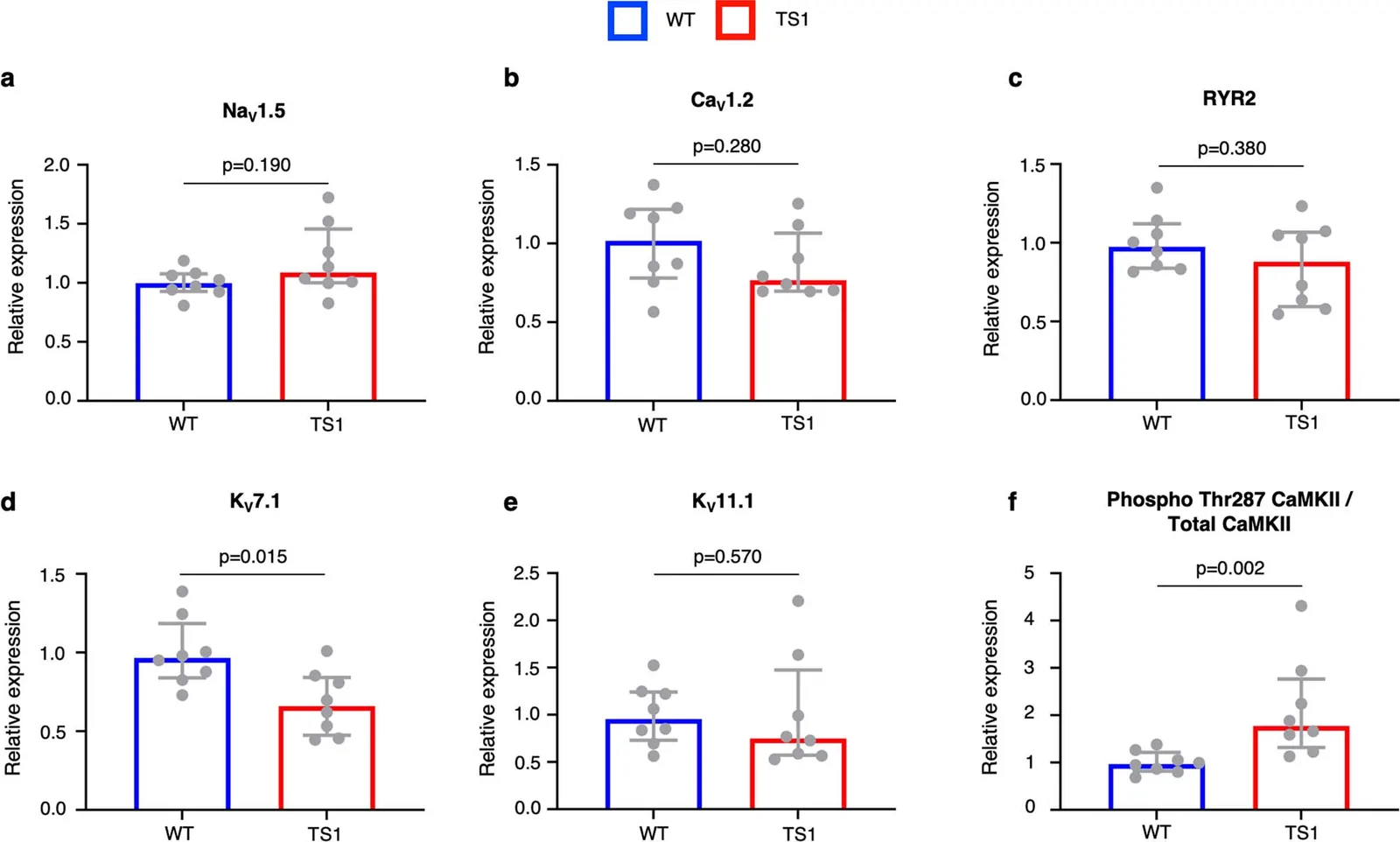
Protein expression evaluation. **a-f**, Pooled data of immunoblots of different proteins from heart homogenates from WT (blue) and TS1 (red) pigs. Each value is represented as median and IQR. Statistical analysis was conducted using two-tailed Mann-Whitney U-test. **a**, Na_V_1.5 relative expression in heart homogenates from *n* = 8 WT animals (minimum 0.81, median 1.00, IQR: 0.93-1.08, maximum 1.19) and *n* = 8 TS1 animals (minimum 0.83, median 1.09, IQR: 1.00-1.46, maximum 1.72), p = 0.190. **b**, Ca_V_1.2 relative expression in heart homogenates from *n* = 8 WT animals (minimum 0.57, median 1.02, IQR: 0.78-1.22, maximum 1.37) and *n* = 8 TS1 animals (minimum 0.70, median 0.77, IQR: 0.70-1.07, maximum 1.25), p = 0.280. **c**, RYR2 relative expression in heart homogenates from *n* = 8 WT animals (minimum 0.82, median 0.97, IQR: 0.84-1.12, maximum 1.35) and *n* = 8 TS1 animals (minimum 0.55, median 0.88, IQR: 0.59-1.07, maximum 1.23), p = 0.380. **d**, K_V_7.1 relative expression in heart homogenates from *n* = 8 WT animals (minimum 0.73, median 0.97, IQR: 0.84-1.18, maximum 1.39) and *n* = 8 TS1 animals (minimum 0.45, median 0.66, IQR: 0.47-0.84, maximum 1.01), p = 0.015. **e**, K_V_11.1 (hERG) relative expression in heart homogenates from *n* = 8 WT animals (minimum 0.56, median 0.96, IQR: 0.73-1.24, maximum 1.53) and *n* = 8 TS1 animals (minimum 0.53, median 0.75, IQR: 0.57-1.47, maximum 2.20), p = 0.570. **f**, Phospho Thr287 CaMKII / total CaMKII relative expression in heart homogenates from *n* = 8 WT animals (minimum 0.69, median 0.97, IQR: 0.82-1.21, maximum 1.38) and *n* = 8 TS1 animals (minimum 1.13, median 1.77, IQR: 1.32-2.77, maximum 4.31), p = 0.002. Panels **a** to **e** refer to Source Data Figs. 1 to 5, respectively; Panel **f** corresponds to Source Data Figs. 6 and 7. Source data

As EADs can arise from an increase in depolarizing currents or a decrease in repolarizing currents, we completed the evaluation of the major ionic currents by studying the potassium currents. Our data show that I_K1_ and I_Kr_ were not different between WT and TS1, whereas we observed a significant reduction in I_Ks_ current in TS1 cardiomyocytes (Extended Data Fig. 9). Interestingly, I_Ks_ reduction was associated with reduction of the amount of protein in the presence of normal transcript levels, in line with evidence from a murine model of TS1 by Song et al.^14^ (Extended Data Fig. 10).

### Dissecting the mechanisms causing the slowing of conduction

Up to this point, EAM studies in TS1 pigs have disclosed a progressive and significant slowing of cardiac activation with the successive addition of premature ventricular extrastimuli, and both CMR and histology showed absence of fibrosis. In the light of the aforementioned and combined with data from the in vitro studies, which showed a physiologically relevant Ca^2+^ overload secondary to the impairment of Ca_V_1.2 inactivation in TS1, we formulated the hypothesis, based on the published evidence of augmented CaMKII activity secondary to increased intracellular Ca^2+^ in a rat model of TS1 (ref. ^8^), that CaMKII-mediated mechanism could also alter peak I_Na_ in our model^34^, thus providing a cellular mechanism underlying the slowing of conduction that we observed in vivo.

**Fig. 8:**
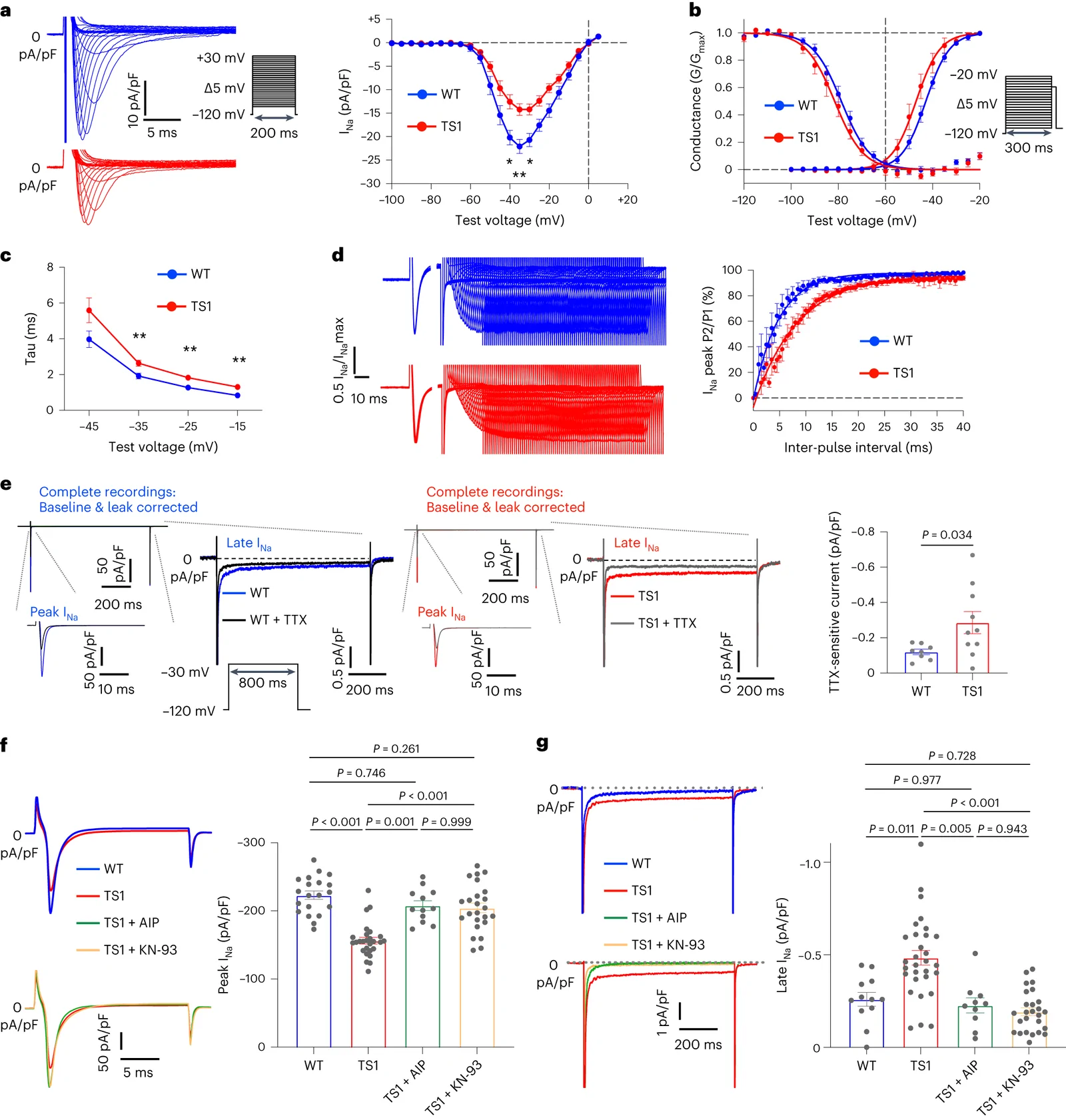
I_Na_ alterations in TS1: regulation by Ca^2+^ and CaMKII. **a**–**d**, Peak I_Na_ in the absence of intracellular Ca^2+^. **a**, Traces of I_Na_ in WT (top, in blue) and TS1 (bottom, in red) cardiomyocytes. Graph shows peak I_Na_ I-V relationships for WT (blue, *n* = 17 cells and *N* = 4 animals) and TS1 (red, *n* = 23 cells and *N* = 4 animals) (*P* = 0.025 at −40 mV, *P* = 0.006 at −35 mV and *P* = 0.025 at −30 mV; two-way ANOVA with Šidák post test). **b**, I_Na_ activation and availability curves for WT (blue, *n* = 14 cells and *N* = 4 animals) and TS1 (red, *n* = 13 cells and *N* = 5 animals) (*P* = 0.025 for availability and *P* = 0.091 for activation; two-tailed nested *t*-test). **c**, I_Na_ inactivation rates WT (blue, *n* = 17 cells and *N* = 4 animals) and TS1 (red, *n* = 23 cells and *N* = 4 animals) (two-way ANOVA *P* = 0.005; *P* = 0.009 at −35 mV, *P* = 0.003 at −25 mV and *P* = 0.003 at −15 mV; two-way ANOVA with Šidák post test). **d**, Recovery from inactivation of I_Na_ for WT (blue, *n* = 12 cells and *N* = 5 animals) and TS1 (red, *n* = 20 cells and *N* = 6 animals) (*P* < 0.0001; two-tailed *F*-test). **e**, Late I_Na_ in the absence of intracellular Ca^2+^. Left, representative recordings of WT (blue) and TS1 (red) cardiomyocytes. Late I_Na_ current was calculated by subtracting traces without and with TTX (10 µM). Right, graph shows the quantification of late I_Na_ for WT (*n* = 8 cells and *N* = 3 animals) and TS1 (red, *n* = 10 cells and *N* = 3 animals) (*P* = 0.034; two-tailed nested *t*-test). **f**, Representative traces (left) and quantification (right) of peak I_Na_ in physiological Ca^2+^ for WT (*n* = 20 cells and *N* = 9 animals), TS1 (*n* = 27 cells and *N* = 7 animals), TS1 + AIP (100 nM) (*n* = 12 cells and *N* = 3 animals) and TS1 + KN93 (1 µM) (*n* = 24 cells and *N* = 7 animals). **g**, Representative traces (left) and quantification (right) of late I_Na_ in physiological Ca^2+^ for WT (*n* = 12 cells and *N* = 5 animals), TS1 (*n* = 30 cells and *N* = 5 animals), TS1 + AIP (100 nM) (*n* = 10 cells and *N* = 3 animals) and TS1 + KN93 (1 µM) (*n* = 26 cells and *N* = 5 animals). Nested one-way ANOVA with Šidák post test (**f**,**g**). Each value is represented as mean ± s.e.m. NS, not significant, *P < 0.05, **P < 0.01 and ***P < 0.001. Source data

Indeed, immunoblotting demonstrated a greater degree of CaMKII autophosphorylation in TS1 hearts (Extended Data Fig. 10). We then characterized I_Na_ in the absence of intracellular calcium (and reduced levels of extracellular Na^+^, which provide optimal voltage control), demonstrating in TS1 a significant 30% reduction of peak I_Na_ (Fig. 8a), not associated with reduced protein levels (Extended Data Fig. 10). Voltage-dependent activation of I_Na_ did not differ among phenotypes, whereas a 5-mV hyperpolarizing shift was found in the V_50_ of steady-state availability in TS1 cardiomyocytes (Fig. 8b). Regarding inactivation kinetics, peak I_Na_ decay (Fig. 8c) and recovery from inactivation (Fig. 8d) were both slower in TS1 cardiomyocytes. We also characterized the late I_Na_ as a tetrodotoxin (TTX)-sensitive current demonstrating a two-fold increase in the current (Fig. 8e).

We then moved to the setting of physiological intracellular Ca^2+^, seeking to confirm the findings in more physiological conditions. To this end, we recorded I_Na_ in pre-paced cardiomyocytes, demonstrating that the relative degree of peak I_Na_ reduction (~30%; Fig. 8f) and late I_Na_ augmentation (approximately two-fold; Fig. 8g) were similar to the values identified in experiments performed in the absence of intracellular Ca^2+^. Finally, to offer conclusive evidence of CaMKII-mediated alteration of both peak and late I_Na_ in our model, we demonstrated that two different CaMKII inhibitors (AIP at 100 nM and KN-93 at 1 µM) were able to restore peak and late I_Na_ to the values observed in WT cardiomyocytes, without differences between AIP and KN-93 (Fig. 8f,g).

Taken together, our findings were consistent with the known multifaceted effects of sodium channel regulation by CaMKII (ref. ^34^), which may be mediated, in part, by the autophosphorylated CaMKII pool. Specifically, these data suggest that, in our model of TS1, a CaMKII-mediated reduction of peak I_Na_ may play an important role in the slowing of impulse propagation.

## Discussion

We developed and characterized a novel swine model of TS1—the first, to our knowledge, knock-in large mammal model of LQTS that fully recapitulates human disease. Combining a variety of in vivo and in vitro techniques, we discovered that constitutional slowing of cardiac activation, caused by the reduction of Na^+^ current density, creates favorable circumstances for the onset and maintenance of reentrant ventricular arrhythmias. These findings offer unexpected insights into the substrate for life-threatening arrhythmias in TS1. We demonstrate that the arrhythmogenic substrate can be studied with clinically available EAM systems and that it may be quantifiable using advanced metrics, such as the RVI, which offers a clinically validated^19^ benchmark to test the efficacy of anti-arrhythmic therapies.

Since the seminal work by Splawski et al.^1^ demonstrated that the p.Gly406Arg mutation in the exon 8A of *CACNA1C* impairs Ca_V_1.2 voltage-dependent inactivation, thus substantially prolonging AP duration in cardiomyocytes, notable advances have been made in the understanding of cellular mechanisms of arrhythmogenesis. Insights from rodent models of TS1 have been pivotal in disclosing that defective Ca_V_1.2 inactivation is the upstream event profoundly upsetting intracellular Ca^2+^ homeostasis and Ca^2+^-mediated signaling, including secondary increased CaMKII activity^7,8^. Despite these remarkable insights at the cellular level, understanding of organ-level mechanisms of arrhythmogenesis in TS1 is currently lacking, to our knowledge.

We, thus, developed a porcine knock-in model of TS1 to study the arrhythmogenesis at the organ level. Porcine models are characterized by a high translational potential, with remarkable similarities to humans in terms of AP shape and duration, ion channel profile, intracellular Ca^2+^-handling dynamics, cardiac anatomy and hemodynamics^35^, best corroborated by a recent groundbreaking porcine-to-human cardiac xenotransplantation^36^. The question of comparable cardiac anatomy and, specifically, cardiac volume assumes a particularly relevant role in the study of organ-level arrhythmogenesis, as both the establishment and maintenance of complex ventricular arrhythmias^37^, as well as the in vivo study of these phenomena using human equipment, are unfeasible in small animal models.

Hitherto, organ-level studies of arrhythmogenesis in LQTS have been performed in canine models of drug-induced QT prolongation, using dofetilide to simulate LQTS type 2 (ref. ^38^) and anthopleurin to mimic LQTS type 3 (ref. ^39^). The results of these seminal works converged on the most credited hypothesis that an increase in depolarizing currents (LQT3) or a decrease in repolarizing currents (LQT2) prolonged APD and promoted the dispersion of repolarization, generating the substrate for the maintenance of reentrant arrhythmias. Under these premises, APD prolongation could cause the onset of EADs, which could generate PVCs and propagate whenever they infringe on the refractory tissue initiating reentrant arrhythmias^18^. However, recent in silico data^16^ showed that the same principles might not necessarily govern *CACNA1C*-related forms of LQTS as other forms of LQTS, and, even more critical, the arrhythmogenic mechanisms of individual *CACNA1C* DNA variants may be mutation specific^16^.

In this context, our work aimed to exploit the novel, large animal model of the disease to provide organ-level as well as cellular insights into the arrhythmogenic trigger and the arrhythmic substrate of TS1.

Regarding the arrhythmogenic trigger, the ECG monitoring with implanted recordings demonstrated, in a third of the animals, the occurrence of lethal ventricular arrhythmias triggered by PVCs, paralleling the onset of human arrhythmias in patients with TS1 (Fig. 1d). Our patch-clamp data in isolated myocytes from TS1 pigs showed that the defective Ca_V_1.2 VDI creates a Ca^2+^ overload state^7^. We then confirmed that the enhanced SR Ca^2+^ content should lead to a greater propensity of late systolic sparks in our TS1 swine model. When fused into Ca^2+^ ripples/waves, such sparks foster the development of EADs in failing ventricular myocytes^32^. Here, we documented the co-occurrence of phase 2 EADs and late Ca^2+^ ripples/waves in TS1. Interestingly, in the voltage range where EADs arose (−36 mV to 0 mV), which is consistent with phase 2 EADs, we documented the coexistence of several abnormally enhanced inward currents that could explain the onset of EADs: the slow-inactivating I_Ca_ (ref. ^40^), the I_ti_ (ref. ^41^) and the late I_Na_ (ref. ^42^). Notably, once they do occur, EADs need to propagate to determine the onset of arrhythmia. It was long thought that phase 2 EADs could not propagate, until 2001 when Yan et al.^43^ documented phase 2 EADs triggering arrhythmias in a wedge preparation of drug-induced LQTS. In tissue areas concomitantly subjected to EADs and steep repolarization gradients, sustained electrotonic diffusion of currents from refractory cells toward resting cells may overcome the source-sink mechanism, promoting the development of arrhythmia-triggering PVCs^44^.

As an additional source for the trigger of arrhythmias in TS1, previous studies in human iPSCs^12^ and mouse models^8^ also showed the presence of DADs in TS1 cells. Although we did not observe such a phenomenon in our TS1 cardiomyocytes, this is not in contrast to the occurrence of EADs, because it is known that higher intracellular Ca^2+^ induces Ca^2+^ oscillations that may manifest as EADs and/or DADs^45^.

Regarding the substrate, EAM in our TS1 model demonstrated the presence of a different scenario that promotes reentry, departing from the previously mentioned leading hypothesis of LQTS, suggesting that arrhythmogenic mechanisms in TS1 may not be the same as those in other forms of LQTS. Replicating the onset of documented spontaneous ventricular arrhythmias by controlled introduction of premature ventricular extrastimuli during sinus rhythm, we showed the development of an activation delay that preceded the dispersion of repolarization, suggesting a primary defect in cardiac activation. This phenomenon could not be reasonably explained by the infringement of the depolarization on the underlying substrate of heterogeneous repolarization, which represents the most validated hypothesis for the occurrence of reentrant arrhythmias in LQTS.

After the confirmation of a Ca^2+^ overload state in our model and based on previous evidence of CaMKII autophosphorylation in a rat model of TS1 (ref. ^8^), we investigated, in our model, the presence of alterations of Na_V_1.5, a well-known downstream target of CaMKII (ref. ^46^). In our porcine model of TS1, CaMKII autophosphorylation modulated the function of Na_V_1.5 in a complex fashion: reducing by 30% the peak I_Na_ current and doubling the late I_Na_ current. These data introduce the concept that the reduction of peak I_Na_ current, which may translate in vivo into remarkable slowing of impulse propagation, is an important unexpected player in the arrhythmogenic substrate of TS1. Also, these findings confirmed that augmentation of late I_Na_ current might at least partially contribute to prolonging the AP.

In our model, the aforementioned in vitro alterations translated in vivo into the spontaneous development of life-threatening ventricular arrhythmias. Corroborating the functional nature of the development of VF in TS1 is a large degree of variability of the consequences of S4 among TS1 animals, which led to the genesis of multiple, variably sized areas of tissue permissive for the institution of functional reentry, as determined by the co-occurrence and co-localization of activation delay and dispersion of repolarization. To better evaluate the dangerous liaison between conduction delay and dispersion of repolarization, we used global RVI^20^. This advanced electrophysiological metric allows for better detection for the spatiotemporal complexity of the arrhythmogenic substrate than the duration of the QT interval on the ECG and permits quantification of the propensity to develop reentrant arrhythmias^19,20^.

Leveraging on the availability of our TS1 model as a unique platform for drug testing and aiming to provide clinically useful information on the electrophysiological effect of various compounds proposed for the treatment of TS1, as well as to challenge the ability of global RVI to detect modifications of the abnormal arrhythmogenic substrate, we tested a range of clinically used and preclinical compounds. Although all tested compounds shortened the QTc interval, EAM-derived global RVI disclosed a range of unexpected electrophysiological effects: from benefit with verapamil and ICA-105574, over a neutral effect with dextromethorphan, to worsening with the sodium channel blockers ranolazine and mexiletine. Taken together, these data imply that high-density EAM, currently not indicated for patients with LQTS^47^, may help to understand the arrhythmogenic substrate during S4 and may provide additional information on the efficacy of anti-arrhythmic therapies.

In conclusion, we developed and characterized a novel knock-in porcine model of TS1, identifying arrhythmogenic mechanisms underlying life-threatening arrhythmias, sharply departing from classical electrophysiology of LQTS. Our data showed that arrhythmia genesis was secondary to the calcium overload state, which led to the development of a substrate characterized by slowing of impulse propagation, the key player of which is a CaMKII-mediated reduction of the peak Na^+^ current. At the same time, the slowly inactivating I_Ca_ and the secondary calcium overload could jointly engender EADs causative of reentry-triggering PVCs. From a translational perspective, our study proved that EAM could be a useful additional tool for comprehending the arrhythmic substrate in LQTS, both in baseline conditions and after pharmacological modulation.

## Methods

### Generation of TS1 knock-in Large White pigs

All animal procedures were conducted following local regulations for the care and use of laboratory animals after authorization by relevant authorities. Specifically, all procedures involving the use of animals in this study were approved by the Animal Welfare Committee of Avantea, carried out following Italian law (D.Lgs 26/2014) and European Union Directive 2010/63/EU regulating animal experimentation, after authorization by relevant authorities (Ministry of Health project no. 252/2017-PR).

Porcine adult fibroblasts (PAFs) were derived from a skin biopsy of a Large White × Landrace hybrid boar with a previous successful record of SCNT. Recipient sows used as surrogate mothers were also of Large White × Landrace hybrid breed.

TS1 knock-in Large White pigs were generated following the general standard operating procedures (cell culture, transfections, SCNT, recipient sow synchronization, surgical embryo transfer and post-implantation development) previously validated and published by Avantea’s research group^48,49^. Detailed methods are reported in the Supplementary Methods. TS1-specific materials and methods will be described in following sections.

### *CACNA1C* gene sequence analyses

The human *CACNA1C* gene comprises 47 exons characterized by several alternatively spliced regions that lead to channels with distinct functional properties. Relevant to TS are two different mutually exclusive splicing variants (exon 8/8A) that encode for two different CACNA1C protein isoforms: 14 (NM_001129840, exon8) and 17 (NM_001129843, exon8A). The porcine *CACNA1C* gene has not been fully characterized previously. We analyzed in silico the High-Throughput Genomic Sequences database of Sscrofa10.2 (https://www.ncbi.nlm.nih.gov/datasets/genome/GCF_000003025.5/) to identify genomic sequences containing the *CACNA1C* gene. We identified two registered sequences (CU462954 and NW 003610292) that permitted us to reconstruct the exon–intron organization of the *CACNA1C* gene completely and its alternative spliced cDNAs homologous to human isoforms 14 and 17. These results were validated by genomic and transcription analyses, performed in selected PAF lines.

### Genome editing and genotyping analyses

Target porcine *CACNA1C* genomic sequence (exon 8A–intron 9) was analyzed using the CRISPOR (http://crispor.tefor.net) platform. We identified the highest rating guide sequence (TCGGTCCTGCTTACCCGCTA-AGG) for the CRISPR–Cas9 system (*Streptococcus pyogenes*), inducing a specific double-strand break one nucleotide before the target one (c.1216G). Selected sgRNA was expressed by cloning the complementary oligos (*CACNA1C*cr1 FW and *CACNA1C*cr1 RV; Supplementary Table 1) into the pX330-U6-Chimeric_BB-CBh-hSpCas9 expression vector, following the protocol described by Cong et al.^50^. The pX330-U6-Chimeric_BB-CBh-hSpCas9 expression vector was a gift from Feng Zhang (Addgene plasmid no. 42230).

The resulting pX330-*CACNA1C*cr1 expression vector was purified (Plasmid Mini Kit, PC-20, Qiagen), and its sequence was verified by Sanger sequencing analyses (Eurofins Genomics).

The desired c.1216G>A point mutation of exon 8A (Extended Data Fig. 2) was inserted synthetizing the asymmetric (+34/−89) ssODN *CACNA1C* OligoCR1 donor (Supplementary Table 1; Ultramer, Integrated DNA Technologies), corresponding to the strand complementary to the target sequence. This single nucleotide change introduced a new *Bve*I (*Bsp*MI) restriction site (GCAGgt) into the exon 8–intron 9 splicing site, useful for positive selection of TS1 primary colonies by polymerase chain reaction restriction fragment length polymorphism (PCR-RFLP) screenings.

WT porcine primary fibroblasts (1 × 10^6^ cells, ID8177) were co-transfected (program V024, Nucleofector, Lonza) with a pX330-*CACNA1C*cr1 plasmid (2 μg) and with the ssODN *CACNA1C* OligoCR1 donor (0.4 nmol).

All the resulting colonies were lysed, and their purified genomic DNA was PCR analyzed (Supplementary Table 1; LA Taq, Takara) using the following conditions: 94 °C for 2 min, 35 cycles of 30 s at 94 °C, 30 s at 55 °C and 45 s at 72 °C, followed by 7-min extension at 72 °C. The resulting amplicons (817 bp) were treated with *Bve*I (Thermo Fisher Scientific). Only the RFLP-positive ones (Extended Data Fig. 2b; Ctr+ = 817 bp + 450 bp + 357 bp) were purified (ExoSAP, Thermo Fisher Scientific) for the following Sanger sequencing analyses (Eurofins Genomics). Amplicons from putative TS1 colonies were finally subcloned in *Escherichia coli* (DH5α, Topo TA Cloning Kit, Thermo Fisher Scientific) to isolate the sequence of each allele. The resulting purified plasmids were Sanger sequenced to identify colonies affected by unpredictable insertions and deletions (INDELs) and to validate ones carrying the desired mutation (*CACNA1C* WT/p.G406R) before using them as nuclear donors for the SCNT experiments. Genotyping analyses were performed to exclude further mutations in the coding sequence of the *CACNA1C* gene.

Potential off-target editing events associated with the guide RNA (gRNA) selected for exon 8A of the porcine *CACNA1C* gene were predicted using the CRISPOR tool implemented on the latest version of the porcine genome assembly (Sscrofa11.1). In a previous analysis done in 2016, we identified 10 possible off-target sites, and, in all of them, we confirmed identity with the reference sequence. More recently, in consideration of its improvements, we interrogated the CRISPOR tool once more, identifying 42 potential off-target sites associated with the selected gRNA. For each of these potential off-target sites, we confirmed the identity with the reference sequence, thereby excluding off-target events.

### RNA extraction from porcine tissues and retrotranscription

Tissues from the LV of both WT and TS1 pigs were homogenized with pestle in a mortar kept in liquid nitrogen. The powder was collected and resuspended in 1 ml of cold PBS and centrifuged at 1,750*g* for 5 min at 4 °C. Total RNA was extracted using an RNeasy Fibrous Tissue Mini Kit (Qiagen) following the manufacturer’s instructions. RNA concentration was measured using a NanoDrop (ND-1000) spectrophotometer, and a total amount of 1 μg of total RNA was used for retrotranscription reaction with an iScript cDNA Synthesis Kit (Bio-Rad) in line with the manufacturer’s instructions.

### Real-time PCR with labeled sequence-specific probes

Gene expression analysis was performed by real-time PCR using TaqMan probes (Thermo Fisher Scientific). The reaction was set up in MicroAmp optical 96-well reaction plates using a ViiA7 Real-Time PCR system (Applied Biosystems). Analysis of the reaction was performed with QuantStudio Real-Time PCR Software 6 and 7 version 1.3 (Applied Biosystems) that generated automatically. Custom Plus TaqMan RNA assays were designed to span exon–exon boundaries for the following sequences:

- assay for swine *CACNA1C* exon 8 in exons 7–8 boundary
- assay for swine *CACNA1C* exon 8A in exons 7–8A boundary
- assay for swine total *CACNA1C* in exons 4–5 boundary
- assay for swine *TBP* (TATA box binding protein) in exons 17–18 boundary

*CACNA1C* mutually exclusive exons (8 and 8A) and constitutive exon 5 expression data were normalized on the endogenous control gene *TBP*. To determine the total expression of *CACNA1C*, the expression of the constitutive exon 5 was analyzed. Exon 8 and exon 8A assays were aimed at quantitative determination of transcripts including either exon 8 or exon 8A.

### In vivo experiments

#### Ethical considerations and animal handling

All animal procedures were conducted following local regulations for the care and use of laboratory animals after authorization by relevant authorities. Specifically, all animal protocols were approved by the Centro Nacional de Investigaciones Cardiovasculares (CNIC) in-house ethical committee, the Universidad Autónoma de Madrid and the Comunidad de Madrid (PROEX 41/17) and conform to European Union Directive 2010/63/EU.

#### Anesthesia and analgesia protocol

For in vivo studies, TS1 pigs of both genders weighing between 60 kg and 80 kg were used for in vivo experiments, and age-matched and sex-matched WT littermates served as controls. After a 12-h fasting period, animals received an intramuscular sedative injection of 2 mg kg^−1^ azaperone (Stresnil) and 5 mg kg^−1^ tiletamine/zolazepam hydrochloride (Zoletil) mix. For lengthy procedures, such as CMR and electrophysiologic study (EPS), endotracheal intubation was performed, and general anesthesia was maintained with isoflurane inhalation (MAC 1.5%) in a synchronized intermittent mandatory ventilation mode, fixing 16 and 20 breaths per minute with tidal volumes between 6 ml kg^−1^ and 8 ml kg^−1^. Veterinarians continuously monitored hemodynamic parameters, including body temperature, heart rate, oxygen saturation and arterial blood pressure, throughout the procedures.

#### 12-lead ECG recording

We recorded the surface ECG at 1 kHz for at least 5 min (Mortara Instruments) (paper speed 25 mm s^−1^ and voltage settings 10 mm mV^−1^) in both TS1 and WT animals. The ECG parameters of interest (PR interval, QRS interval, QT interval and RR interval) were measured using manual calipers at a 25 mm s^−1^ sweep speed. The QT interval duration was measured at a stable heart rate between 50 and 100 beats per minute in lead DII or V5 and corrected (QTc) using the Bazett formula^51,52^.

#### Implantable loop recorder

Using the sedation protocol described and under sterile conditions, the left parasternal region was infiltrated with local anesthesia (1% bupivacaine, B. Braun), and an incision was performed to insert an implantable loop recorder (Medtronic Reveal LINQ or St. Jude Medical CONFIRM Rx) in the intramuscular space. The wound was closed with self-absorbable sutures, and monitoring parameters were activated to record relevant bradyarrhythmia or tachyarrhythmia. After the insertion, the device was interrogated to ensure that optimal sensing was obtained. During follow-up, the devices were interrogated periodically and whenever sudden unexpected death occurred.

#### CMR imaging

CMR studies were performed as described previously^53^. In brief, the CMR study protocol included (1) a segmented cine steady-state free-precession sequence for the assessment of the anatomy, the dimensions, volumetry and global and regional LV and RV function and (2) a T2 mapping sequence, a native T1 mapping and post-contrast T1 mapping sequence and late gadolinium enhancement (LGE) sequence for tissue characterization.

CMR studies were analyzed using dedicated post-processing software (Circle cvi42, Circle Cardiovascular Imaging), as described previously^53^. Ventricular measurements were indexed to body surface area, calculated using the Kelley formula^54^.

#### Histology

Histological samples taken from different sites (atria, RV, interventricular septum and LV) in pigs aged 3–12 months were stained with hematoxylin and eosin and picrosirius red to exclude the presence of inflammatory infiltrates or fibrosis.

#### Cardiac catheterization and pacing

Femoral venous and arterial accesses were obtained with local anesthesia (1% bupivacaine). Under fluoroscopic guidance, a 7-Fr tetrapolar catheter (MarinR Uni, Medtronic) was placed in the RV free wall for pacing. The proximal electrode was positioned in the inferior vena cava and served as an indifferent unipolar electrode. A 6-Fr decapolar catheter (Inquiry, St. Jude Medical) was placed in the right atrium (RA) for pacing. All electrophysiological signals were filtered at 0.5–500 Hz for unipolar signals and 30–250 Hz for bipolar signals. For those experiments requiring epicardial mapping, access was obtained via subxiphoid puncture into the pericardial space^55^.

#### Simultaneous LV and RV endocardial mapping

Assessment of the patterns of activation and recovery with a simultaneous acquisition at 84 endocardial sites (64 in the LV endocardium and 20 in the RV endocardium) was performed with the NavX EnSite Precision system (Abbott Laboratories). Access to the LV was obtained with a retroaortic approach to deploy a 64-electrode basket of 60-mm diameter (Constellation, Boston Scientific) in the LV endocardium. In the RV, a Pentarray catheter (Biosense Webster) with 20 electrodes was deployed in the septal region.

Simultaneous biventricular mapping (LV and RV) was performed during the following pacing protocols:

1. Right atrial pacing at 600-ms and 400-ms cycle length to assess the intrinsic ventricular activation and repolarization.
2. Right ventricular pacing with extra stimulation during intrinsic sinus rhythm with one extra stimulus (S2), two extrastimuli (S3) and three extrastimuli (S4) all close to the ventricular refractory period ((VERP) +30 ms) to simulate spontaneous closely coupled ventricular premature complexes.

LAT was annotated in an automatic fashion with the use of −dV/dt of the unipolar EGM signal during the surface ECG QRS complex occurrence. LRT was annotated in an automatic fashion with the use of +dV/dt of the unipolar EGM signal at the time of the surface ECG T-wave occurrence^56^. For LRT annotation, the mapping window was moved to span the entire T-wave duration independently of its morphology (positive or negative). A fiducial activation was used as a stable timing reference. The QRS onset of the surface ECG was used as time 0 ms to allow comparison between different animals^57^. All electrograms (RV and LV) were collected simultaneously during the same beat. Signals were manually reviewed, and those with noise in the recording or equivocal annotations were discarded. Three-dimensional (3D) points were projected into the surface with an external point filtering set at 7 mm. The NavX output files provide the 3D coordinates of points located on the LV and RV endocardial surfaces, together with the polygonal data structure of the associated surface triangulation and the LAT and LRT values at the same points. Annotations were then exported and collated for analysis with custom software written in MATLAB R2019a (MathWorks), computing the activation–recovery interval (ARI) = LRT − LAT value at each surface point^58^. For each of these three quantities (LAT, LRT and ARI), we computed the 3D map of the distribution of the values over the LV and RV endocardial surfaces by using the MATLAB patch() function.

#### Endo-epicardial high-density EAM

Assessment of the patterns of activation (LAT), repolarization (LRT) and ARI during extrastimulation from the RV with a large number of mapping points was performed with RHYTHMIA HDx Mapping System 3.0. An IntellaMap Orion catheter (Boston Scientific) with 64 printed electrodes distributed equally in eight rows in a steerable basket catheter was used as a roving catheter to acquire sequentially the EAM points in all chambers. The fill threshold for projecting data points into the surface mapped was 2 mm. To ensure that all animals were studied under the same pacing protocol, a first extrastimulus (S2) was delivered as close as possible to the refractory period (VERP +30 ms) during spontaneous sinus rhythm to obtain an S2 map. This was followed by adding a second extrastimulus (S3) coupled as close as possible to the VERP (+30 ms) and the acquisition of S3 maps. This was done with a third extrastimulus (S4) delivered as close as possible to the VERP (+30 ms) to obtain an S4 map. S4 maps were obtained for all animals. S2 and S3 maps were obtained only for a subset of animals to ensure reproducibility of findings of simultaneous mapping with NavX as compared to Rhythmia. The three surfaces (LV endocardium, RV endocardium and epicardium (EPI)) were mapped sequentially for each condition.

Annotation of LAT was performed as described above (−ΔV/Δt of the unipolar signal at the time of the QRS). Because an automatic tool is not currently available for the RHYTHMIA HDx Mapping System, our group, in collaboration with the Research and Development team of Boston Scientific Germany, developed a technique for the annotation of repolarization. In brief, the annotated unipolar signals were inverted using proprietary software of Boston Scientific Germany. Inverted unipolar signals were then re-imported into the proprietary software of Boston Scientific, and the mapping window was moved to span the entire T-wave duration independently of its morphology (positive or negative) and adjusted as needed. The automatically annotated points for both LAT and LRT maps were manually reviewed with a gain of 1 mV mm^−1^ and at a sweep speed of 200 mm s^−1^. Corrections to annotations were limited to isolated outliers to reduce arbitrary measures and ensure reproducibility. A fiducial timing reference for each map was used to ensure that all three chambers mapped were referenced to the same timing annotation. All annotations were then referenced to the RV pacing spike to ensure comparability between animals (time 0 ms). Once the maps were finished, the export tool in the RHYTHMIA system was used. A custom MATLAB code was used to calculate the ARI values based on LRT − LAT at each location^58^.

#### Spatiotemporal gradient calculation

We calculated the spatial gradient of each parameter (LAT, LRT and ARI) on the triangulated mesh of each surface (LV, RV and EPI). We computed, at each vertex of the surface p, the absolute value of the parameter difference quotient over each edge E_pq_ meeting at p (LAT in the following example but analogously for the LRT and ARI distributions):

D_pq_ = |LAT(p) − LAT(q)| / |p−q |. We then define the LAT gradient GradLAT(p) at vertex p as the maximum of these difference quotients: GradLAT(p) = max_q_ D_pq_.

#### Conduction velocity estimation

CV was computed using the triangulation method previously described^59^. This method allows CV estimation from a set of points on a surface, without imposing major constraints on their spacing or distribution^59^. The approach is well suited to the clinical environment, where collected data typically possess these properties^59^.

In brief, using rules of trigonometry, the coordinates of three points (A, B and C) are used in association with their activation times to estimate the average conduction speed and direction within the enclosed triangle, assuming that the wavefront is approximated as locally planar^59^. The angles α and β describe the angle of incidence with respect to the two edges of the triangle meeting at A (a and b). First, the angle θ between the edges a and b meeting at vertex A was computed using equation (1).

1 $$\theta ={\rm{arcos}}\left(\frac{{\left|a\right|}^{2}+{\left|b\right|}^{2}-{\left|c\right|}^{2}}{2\left|a\right|\left|b\right|}\right)$$

Then, the angle α was calculated from equation (2).

2 $$\tan {\rm{\alpha }}\frac{{t}_{b}\left|a\right|-{t}_{a}\left|b\right|\,\cos \theta }{{t}_{a}\left|b\right|\sin \theta }$$

After that, the speed *v* can be computed as described in equation (3).

3 $$\upsilon =\frac{\left|a\right|\cos \alpha }{{t}_{a}}$$

where:

4 $${t}_{a}={LAT}\,\left(B\right)-{LAT}\left(A\right)$$

5 $${t}_{b}={LAT}\,\left(C\right)-{LAT}(A)$$

As recommended^59^, to reduce the impact of measurement errors, we impose constraints on the distance d between vertices (3 ≤ d ≤ 20 mm) and on the difference in activation times (≥3 ms) between vertices.

#### RVI calculation

The RVI has been proposed and validated to predict regions with increased susceptibility to reentrant ventricular tachycardia initiation^19^.

It is defined as the interval between repolarization time (RT) at a site proximal to a line of functional conduction block and activation time (AT) at an adjacent site. The lower the RVI, the more vulnerable the tissue^19,20^.

We calculated the RVI corrected for cycle length as previously described^20^. In brief, RVI was calculated as the minimum difference between LRT at a given site P and LAT at neighboring sites D comprised within a 20-mm radius, after having subtracted the median LRT to all LRTs:

6 $${{RVI}}_{P}=\min ({{LRT}}_{P}-{{LAT}}_{D})$$

The global RVI (RVI_G,D_) was measured as the 10th percentile of the RVI distribution, which represents a robust estimate of the global minimum RVI, as previously described^20^.

#### Programmed electrical stimulation for VF induction

After completing the EAM as described above, we performed programmed electrical stimulation to test the inducibility of VF or tachycardia, following the conventional method used in clinical settings^60^. The protocol consisted of delivery of one, two and three extrastimuli (S2, S3 and S4, respectively) on (1) eight beats of sinus rhythm; (2) eight beats at paced cycle length of 600 ms and (3) eight beats at paced cycle length of 400 ms. Extrastimuli were delivered decrementing cycle length until reaching ventricular refractoriness or a minimum coupling interval of 180 ms.

#### In vivo pharmacological testing

Ranolazine (CVT 303 dihydrochloride) was dissolved with 10% dimethyl sulfoxide, 40% polyethylene glycol 300, 5% Tween 80 and 45% saline solution and administered intravenously (i.v.) as per published protocol (2.5 mg kg^−1^ bolus i.v. followed by 0.135 mg kg^−1^ min^−1^ infusion)^61^. Mexiletine (Mexitil, Boehringer Ingelheim España) was dissolved in 5% dextrose solution and i.v. administered as per published protocol (10 mg kg^−1^)^62^. Verapamil (isoptin 5 mg per 2 ml, Mylan Italia) was dissolved in 0.9% saline solution and i.v. administered as per previously published protocol (0.4 mg kg^−1^)^63^. Metoprolol (seloken 5 mg per 5 ml, Recordati) was dissolved in 0.9% saline solution and i.v. administered as detailed in the work of Van Den Berg et al. (0.3 mg kg^−1^)^64^. Dextromethorphan (dextromethorphan hydrobromide monohydrate, 1 mg ml^−1^, Merck España) was dissolved in 0.9% saline solution and i.v. administered as previously published (0.5–1 mg kg^−1^)^65^. ICA-105574 was synthesized by Giovanni Lentini (Department of Pharmacology, University of Bari) following a recently reported procedure of Zangerl-Plessl et al.^66^ with only one modification: the use of HTBU (CAS no.: 94790-37-1) in lieu of HATU (CAS no.: 148893-10-1) as a coupling reagent. ICA-105574 was dissolved with 30% N,N-dimethylacetamide, 50% polyethylene glycol 400 and 20% water for injection up to 8 mg ml^−1^. ICA-105574 was i.v. administered (10 mg kg^−1^) as detailed in the work of Asayama et al.^29^.

All drugs were administered under continuous electrocardiographic monitoring of the main parameters (heart rate, PR interval, QRS interval and QT interval) as well as monitoring of clinical parameters (oxygen saturation, blood pressure and temperature). At the end of the infusion, endo-epicardial high-density sequential EAM (RHYTHMIA HDx) was repeated according to the same protocol described above.

### In vitro electrophysiological studies

#### Cell isolation

We obtained ventricular myocytes from Large White pigs of both sexes aged 4–6 weeks (weight range, 7–10 kg), using a Langendorff heart perfusion system. The animals were sedated by intramuscular injection (ketamine 20 mg kg^−1^, xilacine 2 mg kg^−1^ and midazolam 0.5 mg kg^−1^). Upon sedation, animals received additional fentanil, 0.005 mg kg^−1^, and sodium heparin, 300 units per kilogram, intravenously and were intubated. Anesthesia was maintained with 3% isoflurane through a non-recirculating anesthesia ventilation circuit. A sternotomy was then performed, and the heart was quickly excised and washed twice through the aorta with ice-cold, high-potassium cardioplegic solution (composition below). The left anterior descending coronary artery was then cannulated and further washed with cardioplegic solution. While in transport from the operating room to the Langendorff system, the heart was continuously perfused with the above ice-cold cardioplegic solution. Once at the Langendorff, the heart was perfused at 37 °C with a nominally Ca^2+^-free Tyrode’s solution (composition below) for 15 min at 7 ml min^−1^. After that, digestion solution (Tyrode’s + Liberase 0.25 mg ml^−1^ + 12.5 µM CaCl_2_ + trypsin 0.14 mg ml^−1^) was applied for 12 min until the heart showed signs of being digested. To stop digestion, the heart was removed from the system; the cannula was removed; and the heart was placed in a warm enzyme-free Tyrode’s + 12.5 µM Ca^2+^ + 0.05 g ml^−1^ BSA solution. The atria, the septum, the RV and all tissues that were not well digested were discarded. The remaining LV wall was minced to pieces with a size of 4–5 mm. The preparation was then filtered through a mesh of pore size 100 µm to obtain the piglet’s LV myocytes. Myocyte-containing pellets were resuspended in a BSA-free solution. Ca^2+^ was gradually increased to physiological values in four steps (200 microM, 400 microM, 600 microM and 1.8 mM). Only elongated cells with clear cross-striations and without granulation were used for experiments.

##### Composition of solutions

Cardioplegic solution (in mM): 110 NaCl, 25.4 KCl, 1.2 KH_2_PO_4_, 10 HEPES, 1 MgCl_2_, 5 Na pyruvate, 20 glucose and 10 taurine, pH 7.4 at room temperature. Nominally Ca^2+^-free Tyrodeʼs (in mM): 130 NaCl, 5.4 KCl, 1.2 KH_2_PO_4_, 10 HEPES, 1 MgCl_2_, 5 Na pyruvate, 20 glucose and 10 taurine, pH 7.4 at room temperature.

### Single-cell electrophysiology

Data were collected using an Axopatch 200B amplifier and pCLAMP software 10.4. Digitization was accomplished with a Digidata 1550B (Molecular Devices). Data were sampled at 10 kHz and filtered at 2 kHz. I-V relationships were obtained with Clampfit 10.6 software (Molecular Devices). Baseline offsets were adjusted before analysis, and corrections of leak were applied, under the assumption that leaks are linear at all voltage ranges. Ionic currents are presented in pA/pF, normalized to cell capacitance. For peak I_Na_ and late I_Na_ measurements, we only patched cells of capacitance 30–60 pF and used pipette tip resistances of 1–3 MΩ. For all other experiments, we did not impose such restrictions in cell capacitance, and electrode tip resistances were in the order of 1.5–4.5 MΩ.

#### APs

APs were measured in current-clamped cells at 36 °C. The external solution was a physiological Tyrode’s saline of composition (in mM) 140 NaCl, 4 KCl, 1.8 CaCl_2_, 1 MgCl_2_, 10 HEPES and 5 glucose, pH 7.4 with NaOH at room temperature. The internal solution for AP recordings contained (in mM): 120 K-aspartate, 20 KCl, 4 Na_2_ATP, 0.4 Na_2_GTP, 10 HEPES, 10 glucose, 4.4205 MgCl_2_ (free Mg^2+^ calculated at 1 mM with MaxChelator software https://somapp.ucdmc.ucdavis.edu/pharmacology/bers/maxchelator/webmaxc/webmaxcS.htm), pH 7.2 with KOH at room temperature. For a subset of cells, 50 µM Rhod-2 was additionally present in the internal solution (see the ‘Confocal Ca^2+^ imaging’ subsection below).

#### Ba^2+^ currents

I_Ba_ was recorded in the whole-cell voltage-clamp configuration at 36 °C. Seals were established and ruptured with cells resting in Tyrode’s solution. Once a constant access resistance had been verified (average, 9 MΩ; range, 4.5–12.2 MΩ), the bath solution was replaced by a recording solution composed of (mM) 134 NaCl, 5 CsCl, 10 HEPES, 5 glucose, 5 BaCl_2_ and 1 MgCl_2_. The internal solution contained (in mM): 125 CsCl, 4 MgATP, 0.3 Na_2_GTP, 10 EGTA, 10 HEPES, 1 MgCl_2_ and total 0.1 CaCl_2_, pH 7.4 with CsOH at room temperature. For the peak I-V curve, from a holding potential of −50 mV, 1-s test pulses were applied to −50 mV to +50 mV in 10-mV increments. Activation and steady-state availability were recorded from a holding potential of −50 mV by means of a P1-P2 protocol. Five-second test pulses were applied between −50 mV and +30 mV in 10-mV increments, and this was followed by a 1-s pulse to 0 mV.

#### Ca^2+^ currents

I_Ca_ was recorded similarly to peak I_Ba_, except that Ba^2+^ in the external solution was replaced by 1.8 mM Ca^2+^. Then, 10 mM sucrose replaced EGTA in the internal solution, and no added calcium was used. Series resistance was, on average, 8.5 MΩ (range, 4.4–12.9 MΩ).

#### Peak I_Na_ in the absence of intracellular Ca^2+^

I_Na_ recordings were carried out at room temperature (22–23.5 °C). The internal solution contained (in mM): 5 NaCl, 135 CsF, 10 EGTA, 5 MgATP and 5 HEPES, pH 7.2 with CsOH at room temperature. Before the electrophysiological recordings, cells rested in Tyrode’s solution. Once a constant low access resistance (average, 7.25 MΩ; range, 3.5–13 MΩ) and good control of the cellular voltage had both been verified, the bath solution was replaced by (in mM) 5 NaCl, 1 MgCl_2_, 1.8 CaCl_2_, 0.1 CdCl_2_, 11 glucose, 132.5 CsCl and 20 HEPES, pH 7.35 with CsOH at room temperature. A 0-mV reversal potential of fast I_Na_ acted as a quality control in the exchange between Tyrodeʼs and recording solution. The I_Na_ activation protocol was carried from a holding potential of −120 mV, using 200-ms test pulses from −120 mV to 30 mV in 5-mV increments.

#### Late I_Na_ in the absence of intracellular Ca^2+^

Late I_Na_ recordings were measured at room temperature (22–23.5 °C) and after application of 10 µM TTX. The internal solution contained (in mM) 5 NaCl, 135 CsF, 10 EGTA, 5 MgATP and 5 HEPES, pH 7.2 with CsOH at room temperature. Before measurements, cells rested in Tyrode’s solution. Once cell access at a constant low access resistance had been verified, the external solution was replaced by (in mM): 140 NaCl, 0.1 CdCl_2_, 11 glucose, 4 CsCl, 20 HEPES, 1 MgCl_2_ and 1.8 CaCl_2_, pH 7.35 with CsOH at room temperature. Late I_Na_ was measured as an average of eight test pulses during 800 ms from −120 mV to −30 mV. R(access) was, on average, 7.5 MΩ (range, 2.7-10.5 MΩ).

#### Fast I_Na_ current in physiological Ca^2+^

Experiments were made to preserve the physiological causal relationship between SR Ca^2+^ release and CaMKII activation by such release. Experiments were conducted at room temperature (22–23.5 °C) in physiological internal and external solutions for AP measurements (see above) at room temperature. Cells with an access resistance ranging between 7 ΩM and 11 ΩM (average, 9 ΩM) were included in the analysis. In voltage-clamp mode, from a holding potential of −100 mV, cells were paced at 1 Hz for 20 s (test potential, 0 mV and 200 ms) to mobilize intracellular calcium. A final step (1 Hz) was given at −40 mV for 20 ms to record peak I_Na_.

CaMKII inhibition was attained by 1-h pre-incubation with either 1 µM N-[2-[N-(4-chlorocinnamyl)N-methylaminomethyl]phenyl]-N(2-hydroxyethyl)-4-methoxybenzenesulfonamide (KN-93) or 100 nM myristoylated autocamtide-2-related inhibitory peptide (AIP). KN-93 or standard AIP was included in the pipette solution. KN-93 was also added to the external solution.

#### Late I_Na_ current in physiological Ca^2+^

Late I_Na_ measurements were conducted at room temperature (22–23.5 °C) with the following internal solution (in mM): 5 NaCl, 135 CsCl_2_, 10 sucrose, 5 MgATP and 5 HEPES, pH 7.2 with CsOH at room temperature. Gigaseals were obtained and ruptured in physiological Tyrodeʼs solution, after which the external solution was replaced by (in mM) 140 NaCl, 11 glucose, 4 CsCl, 20 HEPES, 1 MgCl_2_ and 1.8 CaCl_2_, pH 7.35 with CsOH at room temperature. After the solution replacement, cells were paced for 2–3 min (1 Hz, from −80 mV to 0 mV) to mobilize calcium, either in saline or CaMKII inhibition. After the pacing protocol, 100 µM CdCl_2_ was added to the external solution, and the late I_Na_ was measured as an average of eight test pulses during 800 ms from −120 mV to −30 mV.

CaMKII inhibition was attained as detailed in the subsection ‘Fast I_Na_ current in physiological Ca^2+^’.

#### NCX current

I_NCX_ was recorded following the protocol from Wei et al.^67^ at 36 °C as 5 mM Ni^2+^-sensitive currents. The internal solution contained (in mM): 65 CsCl, 21 EGTA, 20 NaCl, 20 tetraethyl ammonium chloride, 10 HEPES, 5 Na2ATP, 6 total CaCl_2_ (free Ca^2+^ estimated at 51 nM), 4 total MgCl_2_ (free Mg^2+^ estimated at 1.25) and 0.05 ryanodine, pH 7.2 adjusted with CsOH. Gigaseals were obtained with quiescent myocytes resting in Tyrode’s solution. Once the seal had been ruptured and a verification of stable access had been obtained, the external solution was replaced by (in mM): 145 NaCl, 1 MgCl_2_, 5 HEPES, 2 CaCl_2_, 5 CsCl and 10 glucose, pH 7.4, adjusted with NaOH. Ouabain (0.02 mM) and nifedipine (0.01 mM) were added fresh before experiments, as well as 5 mM NiCl_2_ (when needed). NCX currents were measured from a holding potential of −40 mV in 10-s sweeps. For each sweep, a 100-ms step depolarization to +75 mV was followed by a voltage ramp from +75 mV to −115 mV at 100 mV s^−1^, and voltage was maintained at −115 mV for 100 further milliseconds. R(series) was compensated by 70% at a 20-µs lag.

#### Inward-rectifier potassium current

I_K1_ was recorded at 36 °C, as 1 mM Ba^2+^-sensitive current, using the physiological Tyrodeʼs and the internal solution used for AP recordings. R(access) was, on average, 6.4 MΩ (range, 3.5–10.5 MΩ). From a holding potential of −80 mV, 1-s test pulses were applied in the range of −120 mV to 20 mV in 10-mV steps. The I-V was constructed as an end-pulse current.

#### Rapid delayed rectifier current

I_Kr_ was recorded at 36 °C using the whole-cell ruptured patch-clamp technique in the voltage-clamp configuration using borosilicate glass pipettes with a resistance of 2–4 MΩ and filled with a solution containing (in mM) 119 K-aspartate, 15 KCl, 5 MgCl_2_, 4 K_2_ATP, 5 HEPES, 10 glucose and 5 EGTA, pH 7.2 with KOH. The external saline contained (in mM): 132 NaCl, 4 KCl, 1.8 CaCl_2_, 1.2 MgCl_2_, 5 HEPES and 5 glucose, pH 7.4 with NaOH, plus 5 μM nisoldipine. R(access) was 8.8 MΩ on average (range, 4.7–13.6 MΩ). I_Kr_ (defined as a 5 μM E4031-sensitive current) was measured by applying 500-ms pulses from a holding potential of −50 mV to potentials between −50 mV and +30 mV in 10-mV steps. R(access) was compensated to 60–70% with a 20-μs lag.

#### Slow delayed rectifier current

I_Ks_ was recorded in whole-cell voltage-clamp configuration at 36 °C. The internal solution was similar to the AP internal recording solution except for the addition of 5 µM K2EGTA from a K2EGTA stock of pH 7.2. The external saline was Tyrodeʼs solution + 10 μM nisoldipine (to block I_Ca_). The I_Ks_ I-V relationship was calculated as the end-pulse, HMR-1556-sensitive current (100 nM added externally) and was measured using 100-ms pre-pulses to +20 mV followed by 5-s pulses to potentials between −50 mV and 50 mV in 10-mV steps, from a holding potential of −50 mV. R(access) was, on average, 6.65 MΩ (range, 3.5–12.7 MΩ).

#### Confocal Ca^2+^ imaging

In a subset of cells, Ca^2+^ was imaged simultaneously with the electrophysiological recordings. For these experiments, myocytes were dialyzed with 50 µM Rhod-2, which was added to the internal solution. Linescans were recorded using a Zeiss LSM 880 confocal microscope equipped with a ×40/1.4 numerical aperture (NA) oil immersion objective and a zoom of ×3 (pixel size, 138 nm). Time resolution was 1.23 ms per line with an attenuation of the laser power (1 mW) to 0.5%. Rhod-2 was excited at 561 nm, and the emission filters were set between 566 nm and 645 nm, at a pinhole of 1 Airy unit. Cells were scanned through their long axis, avoiding as much as possible the nuclear areas. We assessed both the amplitude (Δ*F*/*F* diastolic) and kinetics of Ca^2+^ transients, including their time to peak, full duration at half magnitude (FDHM) and time to 90% decay from peak signal.

### Protein extraction and Immunoblotting

LV biopsies were excised from the piglets’ hearts after cannulation of the left anterior descending coronary artery (before the procedure for the isolation of cardiomyocytes; see below), washed in ice-cold cardioplegic solution to remove excess of blood and immediately flash frozen in liquid nitrogen. Total tissue homogenates were obtained by pulverization using a mortar and pestle in the presence of liquid nitrogen. Further homogenization of the dry pellet proceeded in a buffer containing 30 mM KH_2_PO_4_ (pH 7.0), 40 mM NaF, 5 mM EDTA, 300 mM sucrose, 0.5 mM DTT and 2× protease inhibitor (Sigma-Aldrich). After one cycle of sonication, the samples were solubilized in one volume of 100 mM Tris-HCl (pH 7.4) and 6% SDS for 1 h at room temperature. The samples were then centrifuged at 21,000*g* for 10 min at 4 °C, and the supernatant was stored. Protein quantification was performed with a Pierce BSA Protein Assay Kit (Thermo Fisher Scientific). Protein samples were suspended in SDS-PAGE loading buffer (30 μg of proteins + β-mercaptoethanol + 4× Laemmli sample buffer) and heated at 92 °C for 10 min. Proteins were separated in a 4–15% SDS-PAGE pre-cast gradient gel (Mini-PROTEAN TGX Stain-Free, Bio-Rad) and transferred to a PVDF membrane by a semi-dry transfer system (Trans-Blot Turbo Transfer System, Bio-Rad). The membranes were blocked with 5% milk in TBS-T buffer (20 mM Tris, 137 mM NaCl and 0.1% Tween 20 at pH 7.4). Primary antibodies were diluted in 3% BSA in TBS-T buffer, and membranes were incubated overnight at 4 °C. Used primary antibodies were: anti-Na_V_1.5 (Cell Signaling Technology, 14421S, 1:1,000), anti-Ca_V_1.2 (Abcam, ab58552, 1:200), anti-RYR2 (Invitrogen, MA3-916, 1:1,000), anti-K_V_7.1 (Abcam, ab84819, 1:1,000), anti-K_V_ 11.1 (hERG) (Cell Signaling Technology, 12889S, 1:1,000), anti-phospho Thr287-CaMKII (Invitrogen, PA537833, 1:1,000) and anti-CaMKII (Invitrogen, PA522168, 1:1,000). Membranes were incubated with suitable HRP secondary antibodies diluted 1:5,000 in 3% BSA in TBS-T buffer (anti-mouse IgG HRP conjugate, Promega, W402B, and anti-rabbit IgG HRP conjugate, Promega, W401B) for 1 h at room temperature, and the bands were detected by ChemiDoc MP Image System (Bio-Rad). Band intensity was quantified by ImageLab software version 6.1 (Bio-Rad), normalized on the stain-free acquisition of the relative gel and analyzed using Microsoft Excel version 16.77.1.

### Data analysis and statistics

Single-cell data were analyzed using Microsoft Excel version 16.77.1 (ionic currents), Clampfit version 10.6.0.13 (ionic currents and APs), MATLAB R2019a for exponential fitting of current decays or in-home coded algorithms (Ca^2+^ transients and late-systolic Ca^2+^ sparks) using Iterative Data Language (version 8.1, Harris Geospatial). Detection of late-systolic calcium sparks was made using the Fiji plug-in SparkMaster (https://sites.google.com/site/sparkmasterhome/). Continuous data were presented as mean ± s.d. or median and interquartile range (IQR), as appropriate. Data on ionic currents are presented as mean ± s.e.m., per convention. Data were compared using a nested design considering the number of cells (*n*) and animals (*N*), using nested Student’s *t*-test, nested one-way ANOVA or repeated-measures two-way ANOVA (both ANOVAs used Šidák post tests), as required. Categorical data were reported as proportions and percentages and compared using the Fisher exact test. Two-tailed *P* values were calculated with the statistical significance threshold set at *P* < 0.05. Details on the number of cells or animals in individual experiments, along with the statistical test used, are reported in the captions of individual figures or in the figures themselves. Measurements were taken from distinct samples. Data were analyzed using RStudio version 4.1.1 and GraphPad Prism version 8 (GraphPad Software).

### Reporting summary

Further information on research design is available in the Nature Portfolio Reporting Summary linked to this article.

## Supplementary information

## Source data

## Data Availability

The data that support the findings in this study are included in the main article and Supplementary Information. Source data are provided with this paper.

## Code availability

The custom-made MATLAB software used for Fig. 2 and Extended Data Fig. 4 are provided in the Source Data. The software used for the analysis of RHYTHMIA data is the property of Boston Scientific and is covered by a data use agreement signed between Boston Scientific and our institution that prevents us from sharing.
