# Supplementary material for: Unexpected impairment of INa underpins reentrant arrhythmias in a knock-in swine model of Timothy syndrome
Source: Nat Cardiovasc Res. 2023 Dec 11;2(12):1291–309. doi: 10.1038/s44161-023-00393-w (PMC11041658; doi:10.1038/s44161-023-00393-w)

nature cardiovascular research

Technical Report

https://doi.org/10.1038/s44161-023-00393-w

Unexpected impairment of I_{Na} underpins reentrant arrhythmias in a knock-in swine model of Timothy syndrome

In the format provided by the authors and unedited

Index of Contents

Supplementary Table 1. Sequences of oligonucleotides used to generate and to characterize the TS1 cloned piglets....2

Supplementary Table 2. 12-lead electrocardiogram (ECG) in TS1 pigs vs. wild-type (WT) controls....3

Supplementary Table 3. Local Activation Time (LAT), Local Recovery Time (LRT), Activation Recovery Interval (ARI) and dispersion of LRT (LRT range) recorded with simultaneous biventricular endocardial electroanatomical mapping (EnSite NavX Precision) during atrial pacing at different pacing rates (100 bpm and 150 bpm)....4

Supplementary Table 4. Local Activation Time (LAT), Local Recovery Time (LRT), Activation Recovery Interval (ARI) and dispersion of LRT (LRT range) recorded with simultaneous biventricular electroanatomical mapping (EnSite NavX Precision) during a programmed electrical stimulation protocol that introduced one to three premature ventricular extrastimuli (S2, S3, S4) on sinus rhythm. 5

Supplementary Table 5. Main electrophysiological parameters and advanced mapping metrics (see main text for details) recorded with ultra-high density sequential endo-epicardial biventricular electroanatomical mapping (RHYTHMIA HDx) during a programmed electrical stimulation protocol that introduced three premature ventricular extrastimuli on sinus rhythm. Values shown correspond to the 3rd extrastimulus (S4)....6

Supplementary Table 6. Changes in the QTc interval at the 12-lead electrocardiogram (ECG) and in the principal electrophysiological metrics in TS1 pigs after administration of different compounds recorded with ultra-high density sequential endo-epicardial biventricular electroanatomical mapping (RHYTHMIA HDx) during a programmed electrical stimulation protocol that introduced three premature ventricular extrastimuli on sinus rhythm. Values shown correspond to the 3rd extrastimulus (S4)....7

Supplementary Table 7. Characterization of Action Potential in WT and TS1 cardiomyocytes at different pacing frequencies....8

SUPPLEMENTARY METHODS 9

SUPPLEMENTARY REFERENCES 11

Supplementary Figure 1. Comparison of 4-6 weeks-old (“young”) and 10-11 months-old (“older”) isolated swine ventricular myocytes....12

SUPPLEMENTARY TABLES

Supplementary Table 1. Sequences of oligonucleotides used to generate and to characterize the TS1 cloned piglets.

Name	Sequence (5’-3’)	Application
CACNA1Ccr1 FW	CACCGTCGGTCCTGCTTACCCGCTA	pX330-CACNA1cr1 cloning
CACNA1Ccr1 RV	AAACTAGCGGGTAAGCAGGACCGAC	
CACNA1C-9	ATCTGGTTCTTGGTTGTGTTGAGC	PCR amplification of Exon8A-Intron9 region from CACNA1C gene (817 bp)
CACNA1C-10	TGTCTGTTTGGCTTGTTTGC	
CACNA1C OligoCR1	TTTGTACTTAACTTGGTTCTCGGTGTCCTTTAGCAGgtaagcaggaccgaggaaaaaggtcttgatttttccatttatctcatttactctttctgctcttcctggcttttattctttttctga	ssODN donor used to introduce the c.1216G>A point mutation

Supplementary Table 2. 12-lead electrocardiogram (ECG) in TS1 pigs *vs*. wild-type (WT) controls.

	WT (N=27)	TS (N=28)	p-value
Male gender (N [%])	18 (67)	17 (61)	0.646
Age (months), median [IQR]	10.6 [9.5-11.4]	10.9 [9.8-11.2]	0.381
Weight (kg), median [IQR]	54 [50-60]	48 [45-51]	<0.001
Temperature (C°), median [IQR]	37.3 [36.9-37.7]	37.4 [36.9-37.6]	0.839
PR (ms), median [IQR]	150 [129-169]	160 [150-172]	0.262
QRS (ms), median [IQR]	80 [75-81]	89 [80-99]	0.006
RR (ms), median [IQR]	774 [680-850]	820 [735-935]	0.025
QT (ms), median [IQR]	440 [380-450]	550 [495-580]	<0.001
QTc (ms), median [IQR]	482 [469-500]	586 [558-615]	<0.001

Statistics: Mann-Whitney U test.

Supplementary Table 3. Local Activation Time (LAT), Local Recovery Time (LRT), Activation Recovery Interval (ARI) and dispersion of LRT (LRT range) recorded with simultaneous biventricular endocardial electroanatomical mapping (EnSite NavX Precision) during atrial pacing at different pacing rates (100 bpm and 150 bpm).

100 bpm			
	Wild-type (N=10)	TS1 (N=10)	p-value
LAT (ms), mean±SD	14±4.0	19±7	0.061
LRT (ms), mean±SD	327±16	360±9	<0.001
ARI (ms), mean±SD	313±17	341±9	<0.001
LRT range (ms), mean±SD	53±22	66±16	0.351
150 bpm			
	Wild-type (N=10)	TS1 (N=10)	p-value
LAT (ms), mean±SD	16±5	23±8	0.033
LRT (ms), mean±SD	246±10	263±9	0.001
ARI (ms), mean±SD	230±12	241±8	0.037
LRT range (ms), mean±SD	49±19	58±19	0.294

Statistics: Nested t-test.

Supplementary Table 4. Local Activation Time (LAT), Local Recovery Time (LRT), Activation Recovery Interval (ARI) and dispersion of LRT (LRT range) recorded with simultaneous biventricular electroanatomical mapping (EnSite NavX Precision) during a programmed electrical stimulation protocol that introduced one to three premature ventricular extrastimuli (S2, S3, S4) on sinus rhythm.

S2 (1st extrastimulus)	Wild-type (N=8)	TS1 (N=10)	p-value
Coupling interval (ms), mean±SD	354±41	426±47	0.004
LAT (ms), mean±SD	46±10	59±17	0.182
LRT (ms), mean±SD	312±30	373±30	0.003
ARI (ms), mean±SD	266±32	315±25	0.016
LRT range (ms), mean±SD	75±15	94±23	0.380
S3 (2nd extrastimulus)			
Coupling interval (ms), mean±SD	276±61	286±51	0.717
LAT (ms), mean±SD	63±13	94±16	<0.001
LRT (ms), mean±SD	279±26	334±20	0.001
ARI (ms), mean±SD	216±27	240±26	0.262
LRT range (ms), mean±SD	98±20	128±32	0.072
S4 (3rd extrastimulus)			
Coupling interval (ms), mean±SD	229±68	238±41	0.724
LAT (ms), mean±SD	75±14	106±18	<0.001
LRT (ms), mean±SD	258±34	303±16	0.026
ARI (ms), mean±SD	184±41	197±31	0.913
LRT range (ms), mean±SD	99±21	136±37	0.017

Statistical Analysis: two-way ANOVA (effect of number of premature stimuli and effect of genotype), with Šidák correction for multiple comparisons.

Supplementary Table 5. Main electrophysiological parameters and advanced mapping metrics (see main text for details) recorded with ultra-high density sequential endo-epicardial biventricular electroanatomical mapping (RHYTHMIA HDx) during a programmed electrical stimulation protocol that introduced three premature ventricular extrastimuli on sinus rhythm. Values shown correspond to the 3rd extrastimulus (S4).

	Wild-type (N=7)	TS1 (N=9)	p-value
LAT (ms), mean±SD	83.6±13.7	152.5±44.5	0.002
LRT (ms), mean±SD	303.6±21.7	378.1±54.0	0.004
ARI (ms), mean±SD	219.7±22.5	223.6±38.3	0.812
LRT range (ms), mean±SD	91.9±20.1	180.4±67.4	0.005
LAT gradient (ms/mm), mean±SD	1.9±0.4	2.9±0.8	0.008
LRT gradient (ms/mm), mean±SD	2.3±0.6	4.1±1.5	0.010
Maximum LAT gradient (ms/mm), mean±SD	6.2±1.1	9.9±2.8	0.005
Maximum LRT gradient (ms/mm), mean±SD	7.2±1.9	13.3±5.3	0.012
Surface with Conduction Velocity <0.5 m/s (%), mean±SD	5.2±2.4	11.2±5.0	0.012
Surface with Conduction Velocity <0.2 m/s (%), mean±SD	0.3±0.2	1.9±1.4	0.010
$RVI_{G,D}$* (ms), mean±SD	-135.6±21.2	-240.3±77.9	0.004

Statistics: Nested t-test.
*As proposed by Orini and colleagues[1].

Supplementary Table 6. Changes in the QTc interval at the 12-lead electrocardiogram (ECG) and in the principal electrophysiological metrics in TS1 pigs after administration of different compounds recorded with ultra-high density sequential endo-epicardial biventricular electroanatomical mapping (RHYTHMIA HDx) during a programmed electrical stimulation protocol that introduced three premature ventricular extrastimuli on sinus rhythm. Values shown correspond to the 3rd extrastimulus (S4).

	Mexiletine (N=3)	Ranolazine (N=3)	Verapamil* (N=3)	Verapamil + Metoprolol* (N=3)	DXM (N=3)	ICA-105574 (N=4)
Δ QTc (%), ms	-62 (-11%)	-91 (-14%)	-135 (-21%)	-118 (-19%)	-69 (-11%)	-149 (-24%)
Δ LAT (%), ms	+57 (+63%)	+ 87 (+105%)	-13 (-12%)	+4 (+4%)	+3 (+3%)	-46 (-28%)
Δ LRT (%), ms	+50 (+15%)	+94 (+27%)	-36 (-10%)	-14 (-4%)	-8 (-2%)	-87 (-21%)
Δ Max. LRT gradient (%), ms	+3.5 (+48%)	+5.2 (+73%)	-2.0 (-24%)	-0.5 (-6%)	-2.1 (-26%)	-11.4 (-69%)
Δ $RVI_{G,D}$ (%), ms	-81 (+56%)	-112 (+83%)	+15 (-9%)	-8 (+5%)	-3 (+2%)	+88 (-32%)

DXM = Dextromethorphan.
* For these experiments, all post-drug data are referenced to the baseline experiments prior to verapamil administration.

Supplementary Table 7. Characterization of Action Potential in WT and TS1 cardiomyocytes at different pacing frequencies.

	Frequency (Hz)	WT (N animals/cells 10-14/17-38)*	TS1 (N animals/cells 5-7/11-14)*	p-value
Max. dV/dt (mV/ms) mean±SD	*0.5*	325.4±31.0	270.8±61.7	0.012
	1	312.4±35.2	264.0±25.9	0.001
	2	301.2±38.8	242.7±68.5	0.042
AP Amplitude (mV) mean±SD	*0.5*	152.4±8.0	154.2±7.3	0.483
	1	152.4±6.9	152.4±17.4	0.999
	2	151.6±2.7	154.8±5.2	0.120
APD 20 (ms) mean±SD	*0.5*	20.2±13.4	30.8±33.1	0.265
	1	19.5±20.6	14.5±7.3	0.282
	2	14.0±6.6	8.6±4.1	0.020
APD 50 (ms) mean±SD	*0.5*	263.1±65.9	582.8±183.7	<0.001
	1	210.3±76.4	379.2±148.7	<0.001
	2	156.6±48.0	229.3±84.3	0.029
APD 90 (ms) mean±SD	*0.5*	384.7±88.9	838.2±332.1	<0.001
	1	338.6±102.2	582.4±163.8	<0.001
	2	259.2±64.6	371.3±73.8	0.001
RMP (mV) mean±SD	*0.5*	-97.0±5.9	-97.7±4.4	0.656
	1	-96.0±5.5	-98.2±3.9	0.123
	2	-96.5±5.0	-99.3±4.8	0.166

* Same as Figure 6C

SUPPLEMENTARY METHODS

Chemicals

All chemicals and reagents were from Sigma (Milan, Italy), unless otherwise stated.

Cell culture and transfection

Primary cultures of fibroblasts were recovered from adult male ear biopsy (pig adult fibroblasts, PAF). The ear biopsy was cut in small pieces with a scalpel blade, then the pieces of tissue were distributed on the surface of gelatin-coated (0.1% gelatin from porcine skin, in water) dishes with 1.5 mL of DMEM/TCM 199 (1:1, D+M) with 20% of FBS. PAF were allowed to growth until 50% of confluence with changes of medium every 3 days, then the pieces of tissue were removed, and the cells subcultured once they reached confluence in D+M, with 10% of FBS 5 ng/mL of bFGF. Growth conditions were at 38°C in an atmosphere composed of 90% N_2, 5% O_2, and 5% CO_2. Exponentially growing cultures were cryopreserved in D+M with 20% FCS and 10% DMSO and stored in liquid nitrogen.

Transfection

The day before transfection, PAFs were tripsinized, counted, and plated into 60 mm dish to have about $1x10^6$ cells at 80% confluency after 24 h. On the day of transfection cells were tripsinized, counted, resuspended in 100 µL of Nucleofector solution (Basic Nucleofector Kit, Prim.Fibroblasts; Lonza, Basel, Switzerland), mixed with pX330-CACNA1cr1 plasmid (2 μg) and with the ssODN CACNA1C OligoCR1 donor (0.4 nmol), transferred into the nucleofection cuvettes, and transfected with V-24 program (Nucleofector, Amaxa). After nucleofection cells were plated in 60-mm culture dishes with fresh medium; 24 h later cells were trypsinised and replated in ten 150 mm culture dishes for 8 days. Resulting well growing primary fibroblast colonies were picked up and expanded for following molecular analyses and for their cryopreservation (D+M; 20% FCS; 10% DMSO). Cells were cultured in D+M with 10% FCS and 5 ng/mL of bFGF in 5% CO_2 and 5% O_2 in humidified air at 38°C.

Preparation of nuclear donor cells and SCNT

One day before nuclear transfer the donor cells were induced into quiescence by serum starvation (0.5% FCS). The cells were prepared by trypsinization 30 min before nuclear transfer, washed and resuspended in SOF[2] supplemented with 25 mM HEPES (H-SOF). Ovaries with corpora lutea were collected at slaughterhouse and transported to the laboratory at 31–33°C. Oocytes were aspirated from follicles larger than 3 mm in diameter, washed, and transferred to maturation. The maturation medium was DMEM-F12 supplemented with 10% (v/v) fetal calf serum, 110 μg/mL sodium pyruvate, 75 μg/mL ascorbic acid, 100 μg/mL glutamine, 5 μg/mL myoinositol, 0.4 mM cystine, 0.6 mM cysteamine, ITS liquid media supplement (insulin, transferrin, selenite, Sigma, 1 μL/mL), gonadotropins (0.05 IU/mL FSH, and 0.05 IU/mL LH; Pergovet 75, Serono), 100 ng/mL long IGF1 (recombinant insulin growth factor I analog), 50 ng/mL long EGF (recombinant epidermal growth factor analog), and 5 ng/mL bFGF (human recombinant). Oocytes were cultured at 38.5°C in 5% CO_2 in humidified air. After 42 h of maturation oocytes were denuded of cumulus cells by vortexing in the presence of hyaluronidase in HSOF and returned to maturation medium. Only oocytes with extruded polar body were selected. NT-embryos were reconstructed following a zona-free method [3-5]. The zona pellucida of oocytes with extruded polar body was digested with 0.5%

pronase in PBS, the oocytes were washed in H-SOF with 10% FCS and returned to maturation medium. All manipulations were performed in H-SOF with 10% FCS. Zona-free oocytes were exposed to cytochalasin B (5 μg/mL) and Hoechst (5 μg/mL) for 5 min prior to enucleation. Metaphase chromosomes were removed under very short exposure to UV light with a blunt enucleation pipette. After enucleation zona-free cytoplasts were individually washed for few seconds in 300 μg/mL phytohemagglutinin P in PBS and then quickly dropped over a single donor cell[6] settled at the bottom of a microdrop of the diluted donor cell suspension. Formed cell couples were washed in 0.3 M mannitol (Ca^{2+}-free, 100 μM Mg^{2+}) solution and fused by double DC-pulse of 1.2 kV/cm applied for 30 μsec at 46 to 48 h after onset of maturation and returned to maturation medium. NT embryos were activated 1–2 h postfusion at 48–50 h of maturation by double DC-pulses of 1.2 kV/cm for 30 μsec applied in 0.3 M mannitol solution, containing 1 mM Ca^{2+} [7] and 100 μM Mg^{2+}, followed by 4-h culture in maturation medium with 5 μg/mL cytochalasin B. NT embryos were cultured in SOF supplemented with essential and nonessential amino acids and with 4 mg/mL BSA (SOFaa) in a modification of the Well-of-the-Well system (WOW)[8]. During embryo culture half of the medium was renewed on day 3 (D3) and on day 5 (D5) with fresh SOFaa.

Recipent sows synchronization, surgical embryo transfer and post implantation development

Estrus was synchronized by feeding 12 mg of alternogest (Regumate, Intervet, Peschiera Borromeo, Italy) per sow for 15 days and injecting 0.15 mg of PgF2a (Dalmazin, Fatro, Ozzano Emilia, Italy) at the 15th day of regumate treatment and 1000 IU of hCG (Chorulon, Intervet) 96 h after the last altrenogest treatment. The SCNT embryos, on D5 of development were transplanted to the uterus of the sows by midventral laparatomy 4 days after ovulation. Pregnancy was examined at day 29, 36, 50, and 62 by ultrasound. Farrowing was induced by and injection of 2 mL of Dalmazin (Fatro) at D114 of gestation.

SUPPLEMENTARY REFERENCES

1. Orini, M., Taggart, P., Hayward, M. & Lambiase, P. D. Optimization of the Global Re-entry Vulnerability Index to Minimise Cycle Length Dependency and Prediction of Ventricular Arrhythmias during Human Epicardial Sock Mapping. *Comput. Cardiol. (2010).* **44**, 1–4 (2017).
2. Tervit HR, Whittingham DG, Rowson LE. Successful culture in vitro of sheep and cattle ova. *J Reprod Fertil*. **30**, 493-7 (1972).
3. Lagutina I, Lazzari G, Duchi R, Colleoni S, Ponderato N, Turini P, et al. Somatic cell nuclear transfer in horses: effect of oocyte morphology, embryo reconstruction method and donor cell type. *Reproduction*. **130**, 559-67 (2005).
4. Lagutina I, Lazzari G, Galli C. Birth of cloned pigs from zona-free nuclear transfer blastocysts developed in vitro before transfer. *Cloning Stem Cells*. **8**, 283-93 (2006).
5. Oback B, Wiersema AT, Gaynor P, Laible G, Tucker FC, Oliver JE, et al. Cloned cattle derived from a novel zona-free embryo reconstruction system. *Cloning Stem Cells*. **5**, 3-12 (2003).
6. Vajta G, Lewis IM, Trounson AO, Purup S, Maddox-Hyttel P, Schmidt M, et al. Handmade somatic cell cloning in cattle: analysis of factors contributing to high efficiency in vitro. *Biol Reprod*. **68**, 571-8 (2003).
7. Cheong HT, Park KW, Im GS, Lai L, Sun QY, Day BN, et al. Effect of elevated Ca(2+) concentration in fusion/activation medium on the fusion and development of porcine fetal fibroblast nuclear transfer embryos. *Mol Reprod Dev*. **61**, 488-92 (2002).
8. Vajta G, Peura TT, Holm P, Páldi A, Greve T, Trounson AO, et al. New method for culture of zona-included or zona-free embryos: the Well of the Well (WOW) system. *Mol Reprod Dev*. **55**, 256-64 (2000).

Supplementary Figure 1. Comparison of 4-6 weeks-old ("young") and 10-11 months-old ("older") isolated swine ventricular myocytes.

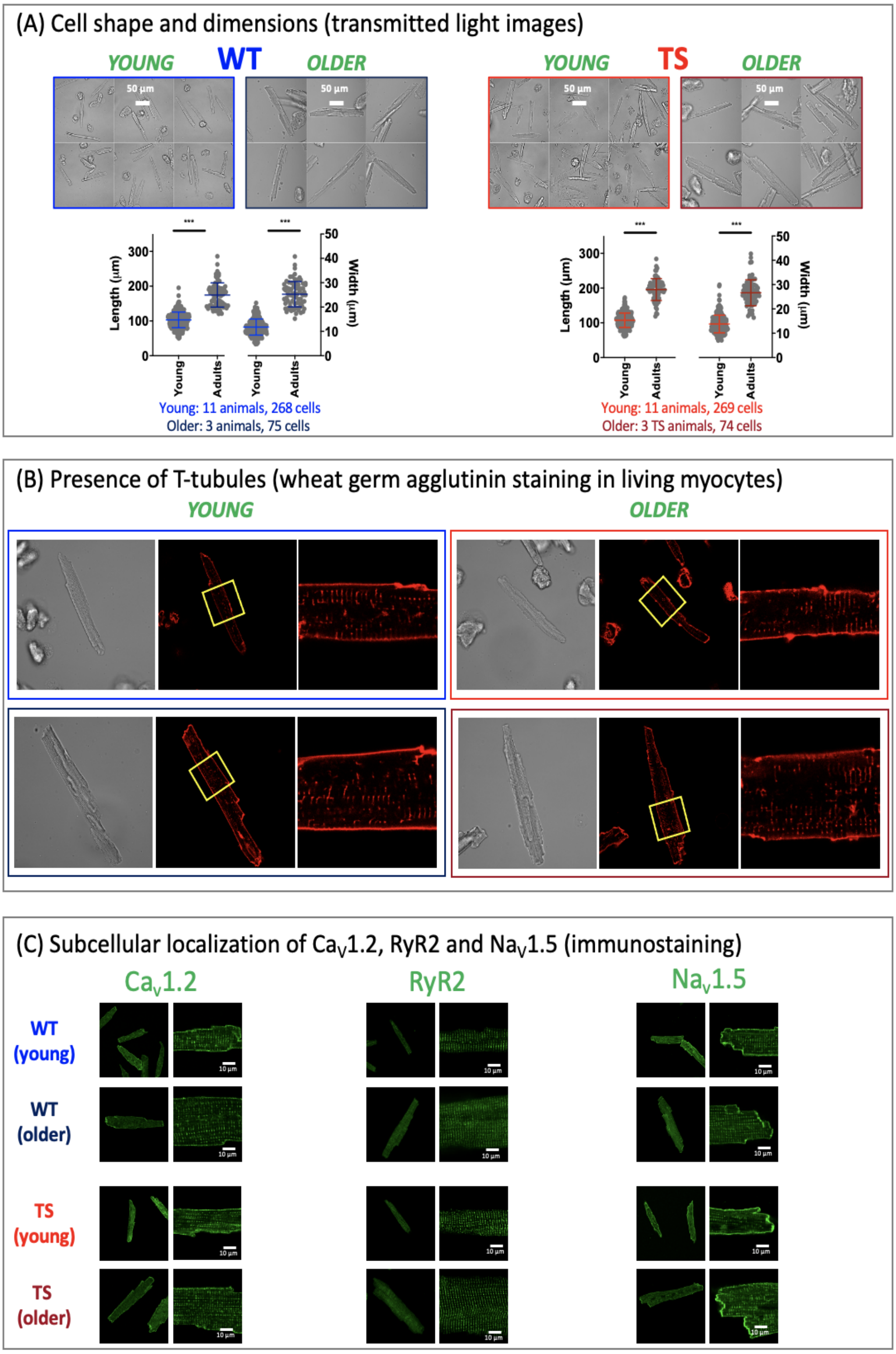

Supplement: Supplementary file 1 — Supplementary Fig. 1, Supplementary Methods and Supplementary References. [file 44161_2023_393_MOESM1_ESM.pdf]
