# Supplementary material for: Unexpected impairment of INa underpins reentrant arrhythmias in a knock-in swine model of Timothy syndrome
Source: Nat Cardiovasc Res. 2023 Dec 11;2(12):1291–309. doi: 10.1038/s44161-023-00393-w (PMC11041658; doi:10.1038/s44161-023-00393-w)
Supplement: Supplementary file 2 — Reporting Summary [file 44161_2023_393_MOESM2_ESM.pdf]

nature portfolio

Corresponding author(s): Professor Silvia G. Priori
silvia.priori@icsmaugeri.it

Last updated by author(s): Nov 14, 2023

Reporting Summary

Nature Portfolio wishes to improve the reproducibility of the work that we publish. This form provides structure for consistency and transparency in reporting. For further information on Nature Portfolio policies, see our Editorial Policies and the Editorial Policy Checklist.

Statistics

For all statistical analyses, confirm that the following items are present in the figure legend, table legend, main text, or Methods section.

n/a	Confirmed	
☐	☒	The exact sample size (*n*) for each experimental group/condition, given as a discrete number and unit of measurement
☐	☒	A statement on whether measurements were taken from distinct samples or whether the same sample was measured repeatedly
☐	☒	The statistical test(s) used AND whether they are one- or two-sided<br>*Only common tests should be described solely by name; describe more complex techniques in the Methods section.*
☒	☐	A description of all covariates tested
☐	☒	A description of any assumptions or corrections, such as tests of normality and adjustment for multiple comparisons
☐	☒	A full description of the statistical parameters including central tendency (e.g. means) or other basic estimates (e.g. regression coefficient) AND variation (e.g. standard deviation) or associated estimates of uncertainty (e.g. confidence intervals)
☐	☒	For null hypothesis testing, the test statistic (e.g. *F*, *t*, *r*) with confidence intervals, effect sizes, degrees of freedom and *P* value noted<br>*Give P values as exact values whenever suitable.*
☒	☐	For Bayesian analysis, information on the choice of priors and Markov chain Monte Carlo settings
☐	☒	For hierarchical and complex designs, identification of the appropriate level for tests and full reporting of outcomes
☒	☐	Estimates of effect sizes (e.g. Cohen's *d*, Pearson's *r*), indicating how they were calculated

Our web collection on statistics for biologists contains articles on many of the points above.

Software and code

Policy information about availability of computer code

Data collection

IN VITRO EXPERIMENTS
• Real Time PCR with labeled sequence-specific probes:
Analysis of the reaction was performed with QuantStudio Real-Time PCR Software 6 and 7 v1.3 (Applied Biosystems) that generated automatically.
• IMMUNOBLOTTING
Band intensity was quantified by ImageLab software v6.1 (Biorad) and analyzed using Microsoft Excel v16.77.1.
• SINGLE CELL ELECTROPHYSIOLOGY AND CA2+ IMAGING
Single-cell electrophysiology. Data were collected using an Axopatch 200B amplifier and pClamp software 10.4. Digitization was accomplished with a Digidata 1550B (Molecular Devices). Data were sampled at 10 kHz and filtered at 2 kHz.
Free Mg2+ was calculated at 1 mM with Maxchelator software (https://somapp.ucdmc.ucdavis.edu/pharmacology/bers/maxchelator/webmaxc/webmaxcS.htm).
Confocal Ca2+ imaging: In a subset of cells, Ca2+ was imaged simultaneously to the electrophysiological recordings. For these experiments, myocytes were dialyzed 50 µM Rhod-2, which was added to the internal solution. Linescans were recorded using a Zeiss LSM 880 confocal microscope equipped with a 40x/1.4N.A oil immersion objective and a zoom of 3x (pixel size 138 nm).
IN VIVO EXPERIMENTS
ELECTROANATOMICAL MAPPING STUDIES
• Custom script written in Matlab R2019a (The MathWorks, Natick, MA, USA) was used for exporting and collating annotated data from NavX Ensite Precision system (Abbott Laboratories, St Paul, MN, USA), as well as for computing ARI for each point (Haws, C. W. & Lux, R. L. Correlation between in vivo transmembrane action potential durations and activation-recovery intervals from electrograms. Effects of interventions that alter repolarization time. Circulation 81, 281–288 (1990)). Custom script written in Matlab was used for the 3D map of the distribution of the values over the LV and RV.

• Custom script written in Matlab R2019awas used for inversion of the annotated unipolar signals for the analysis of LRT using Rhythmia HDx mapping system 3.0 (Boston Scientific, Marlborough, Massachusetts, USA). Custom script written in Matlab was used for computing ARI for each point (Haws, C. W. & Lux, R. L. Correlation between in vivo transmembrane action potential durations and activation-recovery intervals from electrograms. Effects of interventions that alter repolarization time. Circulation 81, 281–288 (1990)). Custom script written in Matlab was used for the calculation of spatiotemporal gradients (LAT gradient, LRT gradient, ARI gradient), conduction velocity (Cantwell, C. D. et al. Techniques for automated local activation time annotation and conduction velocity estimation in cardiac mapping. Comput. Biol. Med. 65, 229–242 (2015)), and reentry vulnerability index (Orini, M., Taggart, P., Hayward, M. & Lambiase, P. D. Optimization of the Global Re- entry Vulnerability Index to Minimise Cycle Length Dependency and Prediction of Ventricular Arrhythmias during Human Epicardial Sock Mapping. Comput. Cardiol. (2010). 44, 1–4 (2017)).

Data analysis

• Analysis of calcium transients and preprocessing of images for detection of late-systolic calcium sparks was done using custom scripts written using Iterative Data Language (IDL version 8.1, Harris Geospatial), which are available upon request. Detection of late-systolic calcium sparks was made using the Fiji plug-in SparkMaster (https://sites.google.com/site/sparkmasterhome/).
• Bi-exponential fitting to ICa and IBa decay was performed using a custom script written in the Matlab language (MATLAB R2019a, The MathWorks, Natick, MA, USA), which is available upon request. Mono-exponential fitting to INa decay was performed using a custom script written in the Matlab language ((MATLAB R2019a, The MathWorks, Natick, MA, USA), which is available upon request.
• Iti integration during SR Ca2+ content measurements were performed using custom scripts written in the IDL language (IDL version 8.1, Harris Geospatial), which are available upon request.
• I-V relationships were obtained with Clampfit 10.6.0.13 software (Molecular Devices). Baseline and leak correction of ionic currents have been applied as needed.
• Circle cvi42. Circle Cardiovascular Imaging Inc., Calgary, Canada. URL https://www.circlecvi.com (proprietary software).
• Analysis of electrophysiological parameters was done using proprietary Boston Scientific software.
• GraphPad PRISM Version 8. GraphPad Software, Inc. La Jolla, CA, USA. URL https://www.graphpad.com (proprietary software)
• RStudio version 4.1.1, R Core Team (2021). R: A language and environment for statistical computing. R Foundation for Statistical Computing, Vienna, Austria. URL https://www.R-project.org/ (open source software).
• A custom analysis script relying on R and Rstudio and MATLAB R2019a (The MathWorks, Natick, MA, USA). The script is available upon request.

For manuscripts utilizing custom algorithms or software that are central to the research but not yet described in published literature, software must be made available to editors and reviewers. We strongly encourage code deposition in a community repository (e.g. GitHub). See the Nature Portfolio guidelines for submitting code & software for further information.

Data

Policy information about availability of data

All manuscripts must include a data availability statement. This statement should provide the following information, where applicable:

- Accession codes, unique identifiers, or web links for publicly available datasets
- A description of any restrictions on data availability
- For clinical datasets or third party data, please ensure that the statement adheres to our policy

The data that support the findings in this study are included in the main article and associated files. Source data are provided with this paper.
Web links for publicly available datasets have been provided when appropriate (e.g. Sscrofa10.2: https://www.ncbi.nlm.nih.gov/datasets/genome/GCF_000003025.5/).

Research involving human participants, their data, or biological material

Policy information about studies with human participants or human data. See also policy information about sex, gender (identity/presentation), and sexual orientation and race, ethnicity and racism.

Reporting on sex and gender	This work does not include studies on human subjects.
Reporting on race, ethnicity, or other socially relevant groupings	This work does not include studies on human subjects.
Population characteristics	This work does not include studies on human subjects.
Recruitment	This work does not include studies on human subjects.
Ethics oversight	This work does not include studies on human subjects.

Note that full information on the approval of the study protocol must also be provided in the manuscript.

Field-specific reporting

Please select the one below that is the best fit for your research. If you are not sure, read the appropriate sections before making your selection.

☒ Life sciences ☐ Behavioural & social sciences ☐ Ecological, evolutionary & environmental sciences

For a reference copy of the document with all sections, see nature.com/documents/nr-reporting-summary-flat.pdf

Life sciences study design

All studies must disclose on these points even when the disclosure is negative.

Sample size	The sample size was calculated using the KISS (Keep It Simple and Straightforward) principle, as informed by relevant previous studies on similar subjects (PMID: 25173890, 10525495, 17954406). To enhance the accuracy of the calculation, the "resource equation" was also considered, and a power analysis was performed to detect a 10% difference in the duration of ventricular repolarization between WT (Wild Type) and TS1 (Treatment Group 1) pigs, with a power of 95% and a significance level of 5% (Festing MF. On determining sample size in experiments involving laboratory animals. Lab Anim. 2018;52:341-350).<br>For each experiment and graph the exact experimental number of cells (n) and animals (N) are provided.
Data exclusions	No data were excluded from the analysis.
Replication	For in vivo studies, we used three or more animals per group in each experiment.<br>For in vitro studies, we generally used three or more animals per group. However, an unexpected increase in animal usage, driven by the need to respond to reviewers' requests (totaling 50 animals), occasionally resulted in fewer animals being available for certain experiments.<br>We were able to produce similar results in the independent experiments, as well as in replicates at each condition.
Randomization	No randomization was required in this study since, when drugs were used, comparisons were made in the same animal.
Blinding	Groups were defined by genotype and data collection was not blinded. Nonetheless, after collection, analyses of data were performed in a blinded fashion.

Reporting for specific materials, systems and methods

We require information from authors about some types of materials, experimental systems and methods used in many studies. Here, indicate whether each material, system or method listed is relevant to your study. If you are not sure if a list item applies to your research, read the appropriate section before selecting a response.

Materials & experimental systems

n/a	Involved in the study	
☐	☒	Antibodies
☒	☐	Eukaryotic cell lines
☒	☐	Palaeontology and archaeology
☐	☒	Animals and other organisms
☒	☐	Clinical data
☒	☐	Dual use research of concern
☒	☐	Plants

Methods

n/a	Involved in the study	
☒	☐	ChIP-seq
☒	☐	Flow cytometry
☒	☐	MRI-based neuroimaging

Antibodies

Antibodies used	• Anti-CaMKII delta Rabbit Polyclonal Antibody; Supplier: Thermo Fisher Scientific; Catalog number: PA5-22168; Lot. Number: WK3434338C; Working dilution: 1:1000.<br>• Anti-Phospho-CaMKII beta/gamma/delta (Thr287) Rabbit Polyclonal Antibody; Supplier: Thermo Fisher Scientific; Catalog number: PA5-37833; Lot. Number: XB3500973; Working dilution: 1:1000.<br>• Anti NaV1.5 rabbit monoclonal antibody; Supplier: Cell Signaling; Catalog number: 14421; Lot. Number: 1; Clone number: D9J7S; Working dilution: 1:1000.<br>• Anti-CACNA1C rabbit polyclonal antibody; Supplier: AbCam; Catalog number: ab58552; Lot. Number: GR33214682-1; Working dilution: 1:200.<br>• Anti-Ryanodine Receptor mouse monoclonal antibody; Supplier: Thermo Fisher Scientific; Catalog number: MA3-916; Lot. Number: XB341933; Clone number: C3-33; Working dilution: 1:1000.<br>• Anti-KCNQ1 mouse monoclonal antibody; Supplier: AbCam; Catalog number: ab84819; Lot. Number: GR3322071-5; Clone number: N37A/10; Working dilution: 1:1000.<br>• Anti-hERG1a rabbit monoclonal antibody; Supplier: Cell Signaling; Catalog number: 12889; Lot. Number: 1; Clone number: D1Y2J; Working dilution: 1:1000.<br>• Anti-Mouse IgG HRP Conjugate; Supplier: Promega; Catalog number: W402B; Lot. Number: 0000457053; Working dilution: 1:5000.<br>• Anti-Rabbit IgG HRP Conjugate; Supplier: Promega; Catalog number: W401B; Lot. Number: 0000407624; Working dilution: 1:5000.
Validation	• Anti-CaMKII delta Rabbit Polyclonal Antibody: https://www.thermofisher.com/antibody/product/CaMKII-delta-Antibody-Polyclonal/PA5-22168<br>This antibody has been predicted by the manufacturer website to react with pig's samples at 100%. We tested it for WB on cardiac protein extract from pig in the present manuscript.<br>• Anti-Phospho-CaMKII beta/gamma/delta (Thr287) Rabbit Polyclonal Antibody: https://www.thermofisher.com/antibody/product/Phospho-CaMKII-beta-gamma-delta-Thr287-Antibody-Polyclonal/PA5-37833<br>"This Antibody was verified by Cell treatment to ensure that the antibody binds to the antigen stated." We tested it for WB on cardiac protein extract from pig in the present manuscript.

• Anti NaV1.5 rabbit monoclonal antibody: https://www.cellsignal.com/products/primary-antibodies/nav1-5-d9j7s-rabbit-mab/14421
We tested it for WB on cardiac protein extract from pig in the present manuscript.
• Anti-CACNA1C rabbit polyclonal antibody: https://www.abcam.com/products/primary-antibodies/cacna1c-antibody-ab58552.html
We tested it for WB on cardiac protein extract from pig in the present manuscript.
• Anti-Ryanodine Receptor mouse monoclonal antibody: https://www.thermofisher.com/antibody/product/Ryanodine-Receptor-Antibody-clone-C3-33-Monoclonal/MA3-916
"This Antibody was verified by Relative expression to ensure that the antibody binds to the antigen stated." This antibody was already used on pig's ventricular myocytes in immunocytochemistry (PMID: 12456488). We tested it for WB on cardiac protein extract from pig in the present manuscript.
• Anti-KCNQ1 mouse monoclonal antibody: https://www.abcam.com/products/primary-antibodies/kcnq1-antibody-n37a10-ab84819.html
We tested it for WB on cardiac protein extract from pig in the present manuscript.
• Anti-hERG1a rabbit monoclonal antibody: https://www.cellsignal.com/products/primary-antibodies/herg1a-d1y2j-rabbit-mab/12889
We tested it for WB on cardiac protein extract from pig in the present manuscript.

Animals and other research organisms

Policy information about studies involving animals; ARRIVE guidelines recommended for reporting animal research, and Sex and Gender in Research

Laboratory animals	Animal experiments were performed in Large White x Landrace 572 hybrid breed pigs with or without the TS mutation (p.Gly406Arg on CACNA1C) of both sexes. The age for in vitro studies was 4-6 weeks. For in vivo studies, animals weighing between 60 and 80 kg were used, corresponding to an age between 7 and 11 months.
Wild animals	No wild animals were used.
Reporting on sex	Animals of both genders were used in the study, as also patients with TS are of both genders. No association of gender with outcomes exists for patients with TS.
Field-collected samples	No samples collected from the field were used.
Ethics oversight	Regarding model creation, all procedures involving the use of animals in this study were approved by the Animal Welfare Committee of Avantea, carried out following the Italian Law (D.Lgs 26/2014) and European Union Directive 2010/63/EU regulating animal experimentation after authorization by relevant authorities (Ministry of Health project n° 252/2017-PR).<br>Regarding in vivo experiments, all animal protocols were approved by CNIC's in-house ethical committee, the Universidad Autónoma de Madrid and the Comunidad de Madrid (PROEX 41/17) and conform to the European Union Directive 2010/63/EU.

Note that full information on the approval of the study protocol must also be provided in the manuscript.
