# Supplementary figures and images for: Unexpected impairment of INa underpins reentrant arrhythmias in a knock-in swine model of Timothy syndrome
Source: Nat Cardiovasc Res. 2023 Dec 11;2(12):1291–309. doi: 10.1038/s44161-023-00393-w (PMC11041658; doi:10.1038/s44161-023-00393-w)

Source Data Figure 1 for Extended Data Figure 10a. Uncropped Western Blots for Nav1.5.

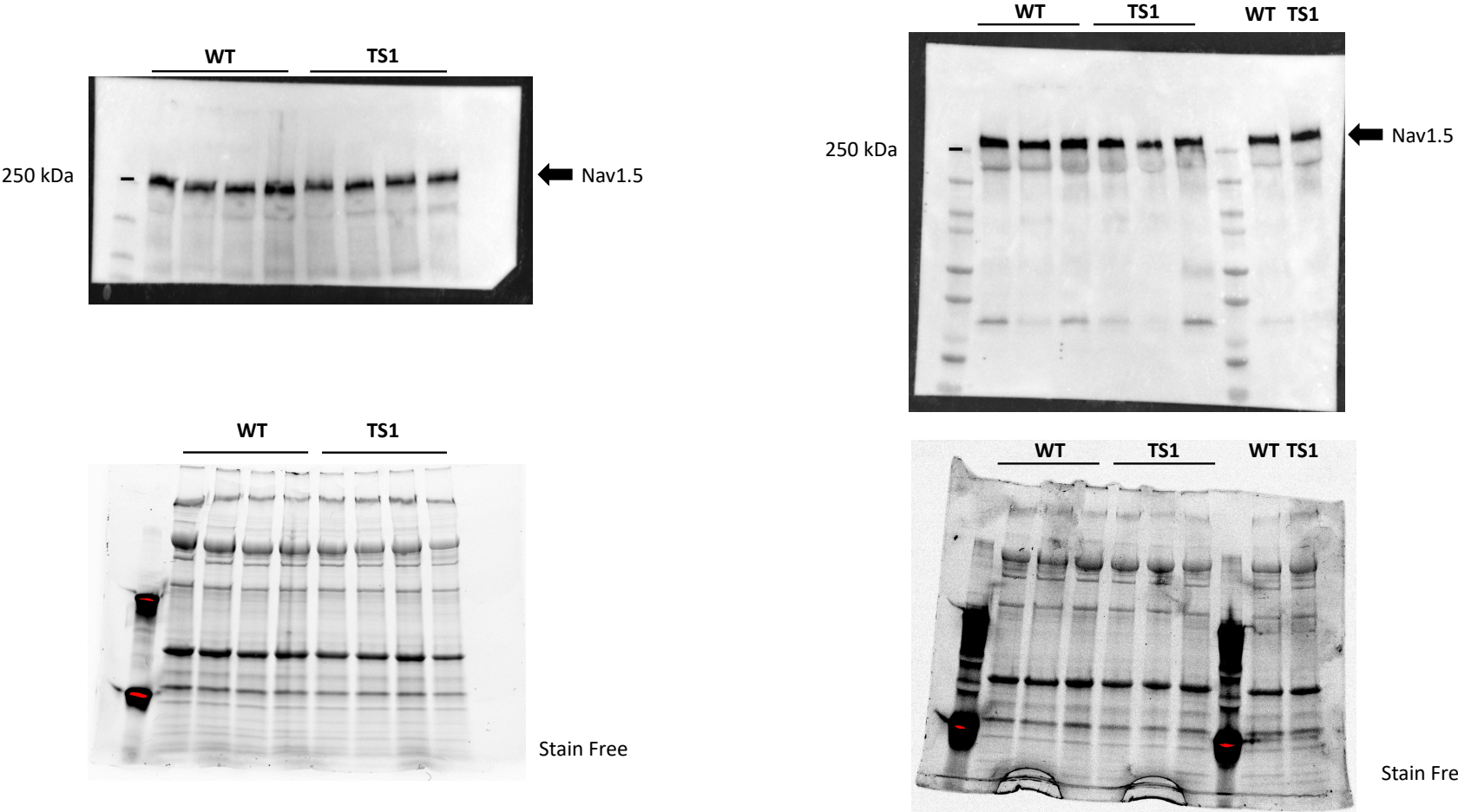

Supplement: Supplementary file 22 — Uncropped western blot for NaV1.5. [file 44161_2023_393_MOESM22_ESM.pdf]

Source Data Figure 2 for Extended Data Figure 10b. Uncropped Western Blots for CaV1.2.

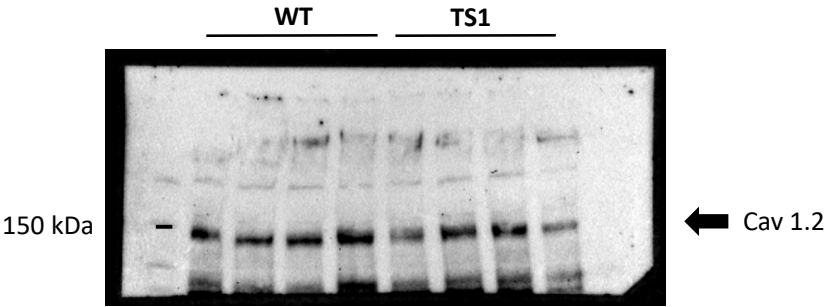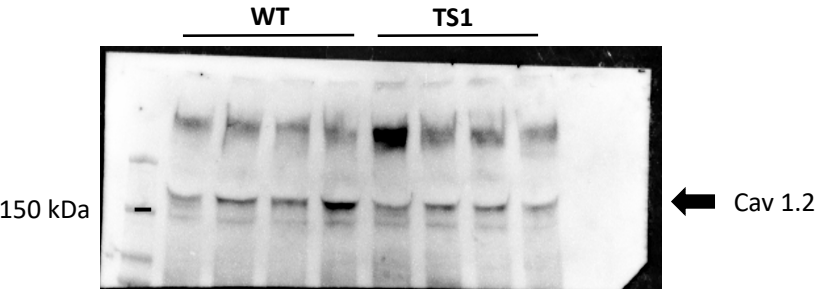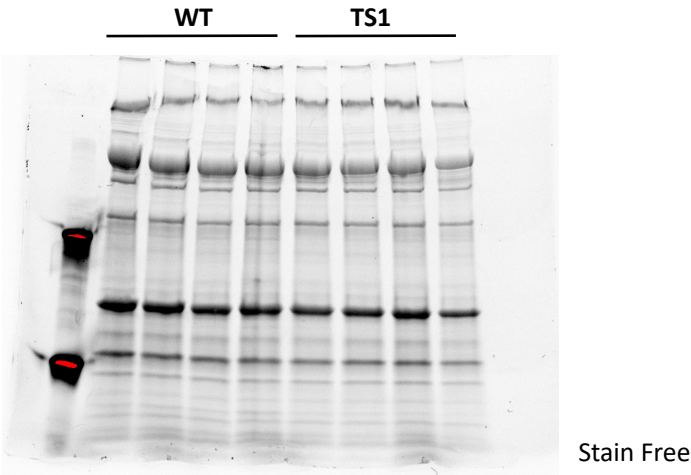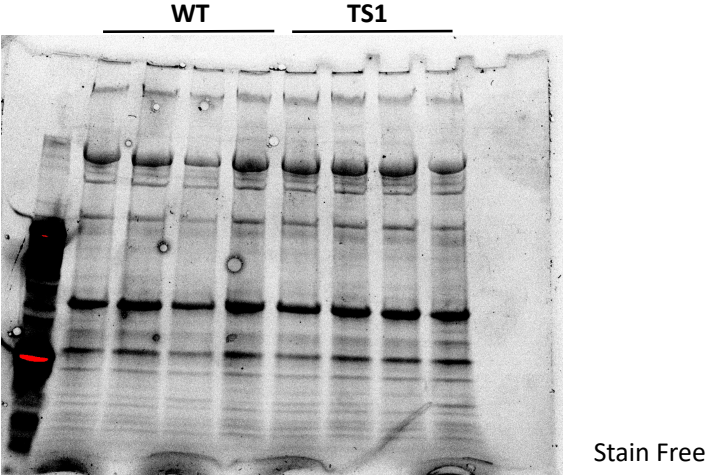

Supplement: Supplementary file 23 — Uncropped western blot for CaV1.5. [file 44161_2023_393_MOESM23_ESM.pdf]

Source Data Figure 3 for Extended Data Figure 10c. Uncropped Western Blots for RyR2.

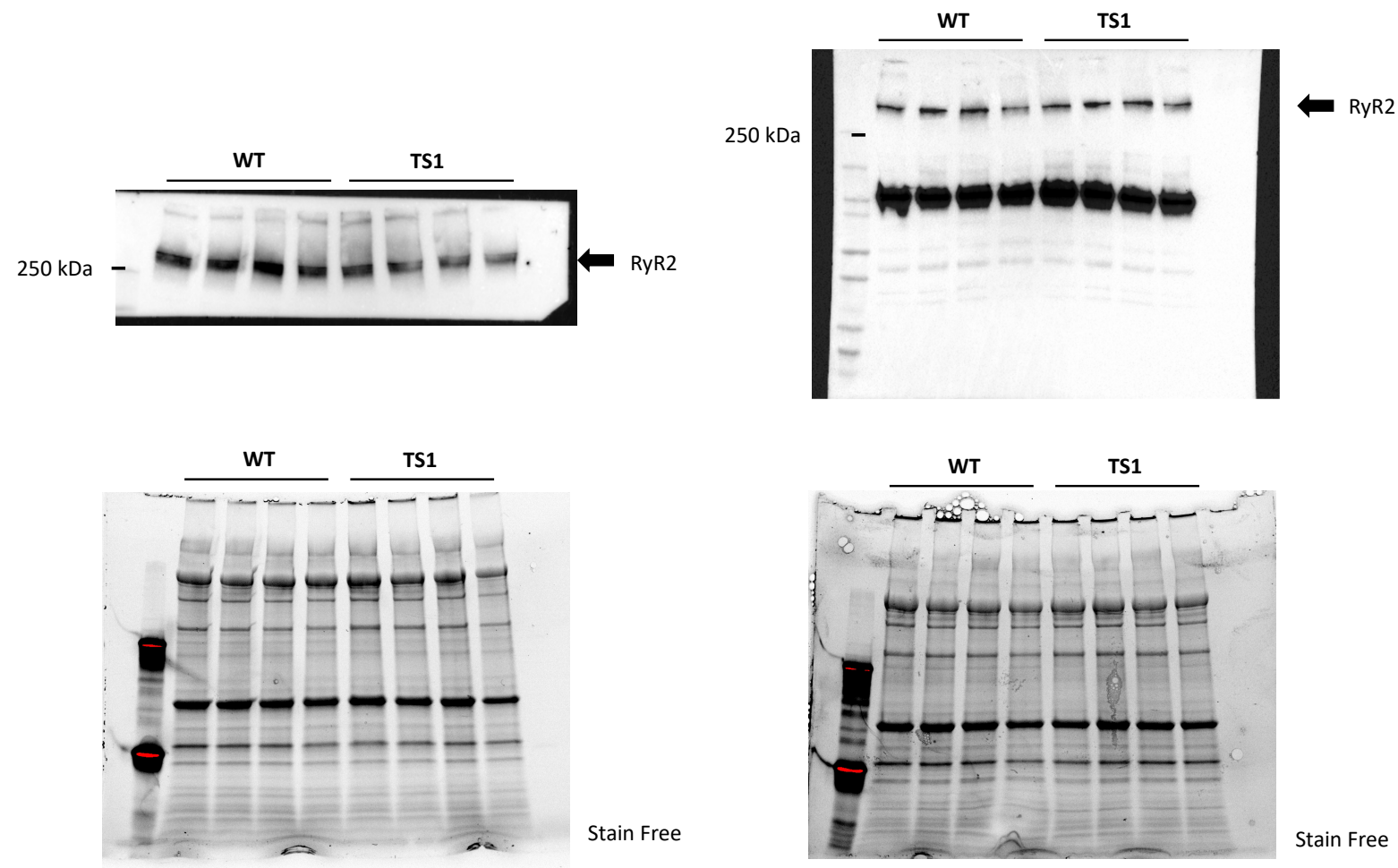

Supplement: Supplementary file 24 — Uncropped western blot for RYR2. [file 44161_2023_393_MOESM24_ESM.pdf]

Source Data Figure 4 for Extended Data Figure 10d. Uncropped Western Blots for Kv7.1.

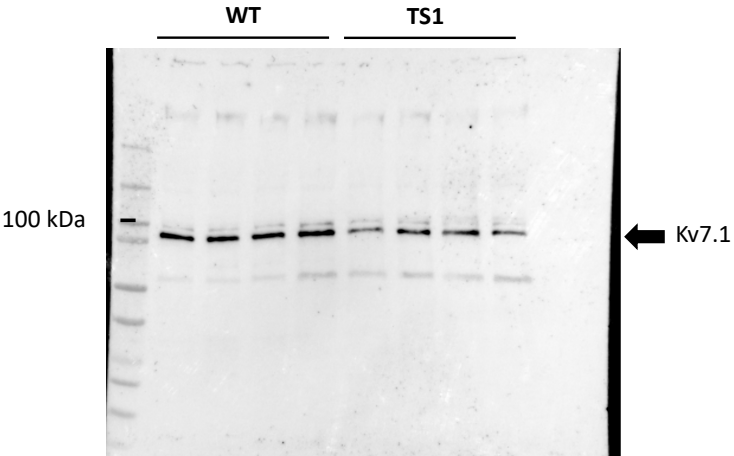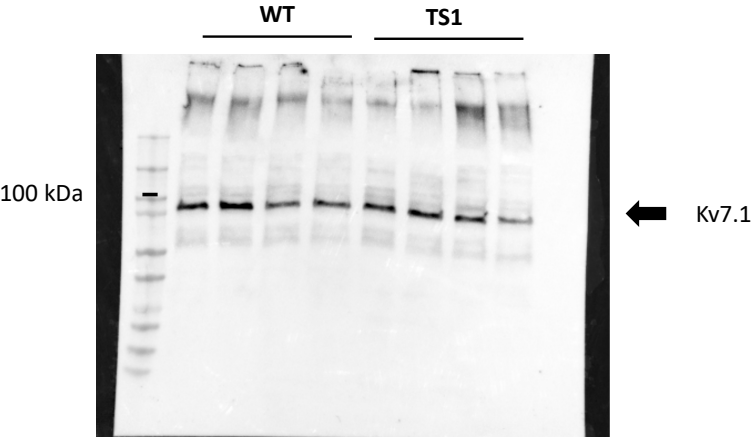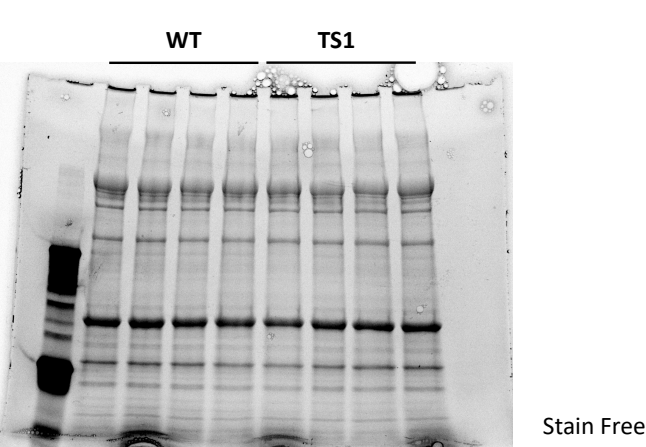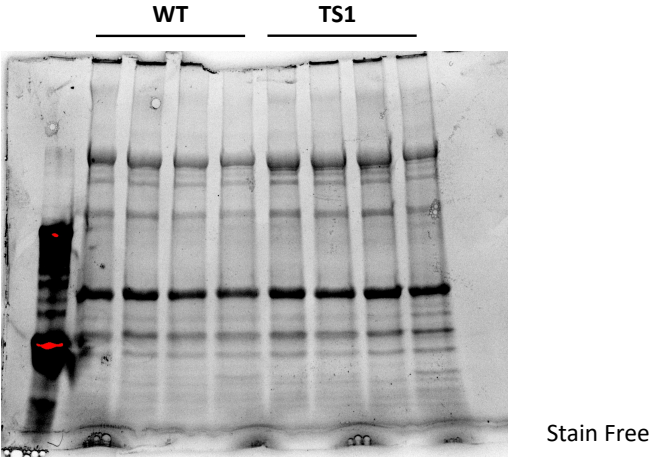

Supplement: Supplementary file 25 — Uncropped western blot for KV7.1. [file 44161_2023_393_MOESM25_ESM.pdf]

Source Data Figure 5 for Extended Data Figure 10e. Uncropped Western Blots for Kv11.1.

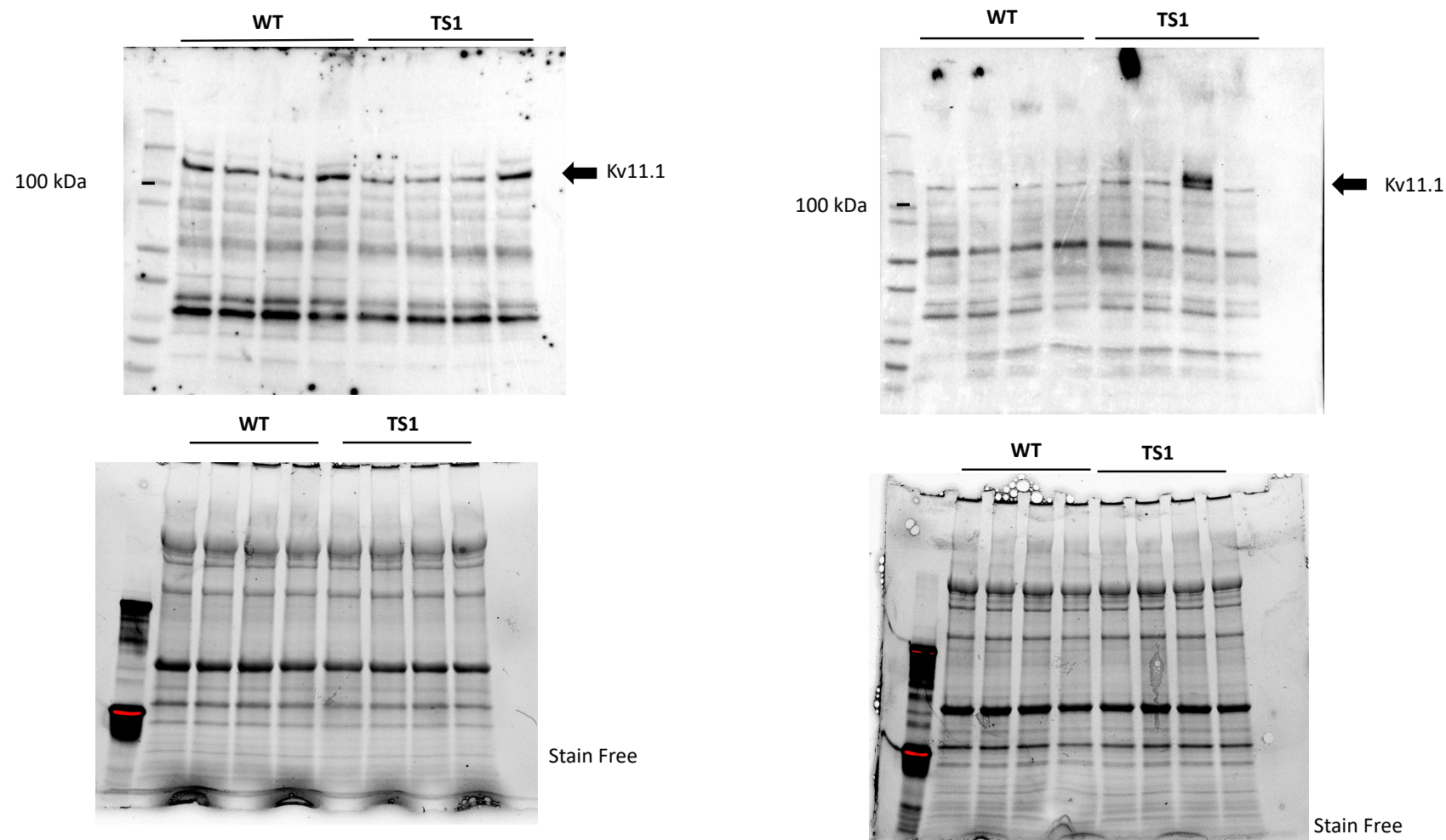

Supplement: Supplementary file 26 — Uncropped western blot for KV11.1. [file 44161_2023_393_MOESM26_ESM.pdf]
