# Supplementary material for: Unexpected impairment of INa underpins reentrant arrhythmias in a knock-in swine model of Timothy syndrome
Source: Nat Cardiovasc Res. 2023 Dec 11;2(12):1291–309. doi: 10.1038/s44161-023-00393-w (PMC11041658; doi:10.1038/s44161-023-00393-w)

Source Data Figure 6 for Extended Data Figure 10f. Uncropped Western Blots for Phospho Thr286 CaMKII (membrane 1).

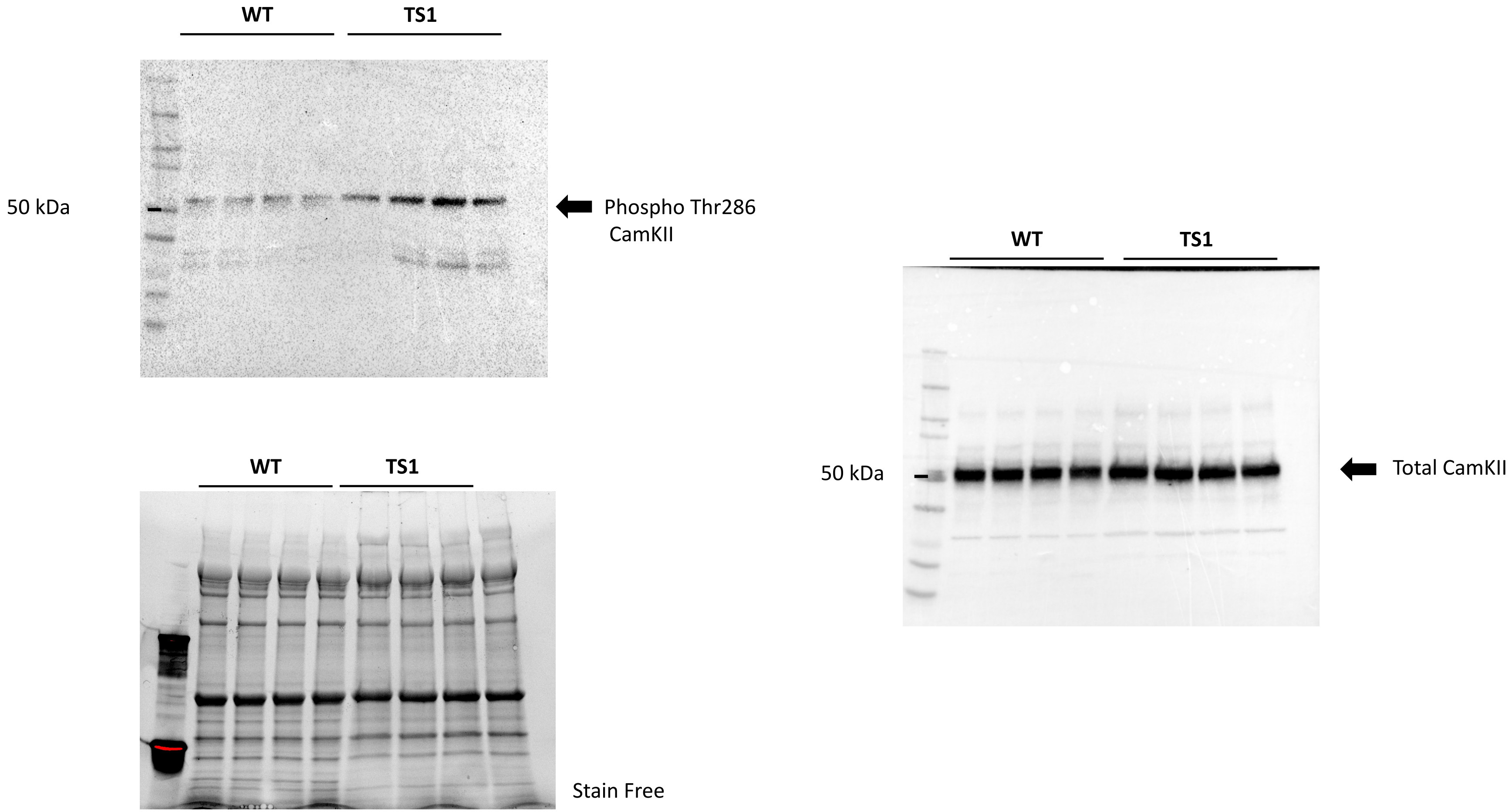

Supplement: Supplementary file 27 — Uncropped western blot for phospho Thr286 CaMKII (membrane 1). [file 44161_2023_393_MOESM27_ESM.pdf]
